# Microhabitat change drives diversification in pholcid spiders

**DOI:** 10.1186/s12862-018-1244-8

**Published:** 2018-09-19

**Authors:** Jonas Eberle, Dimitar Dimitrov, Alejandro Valdez-Mondragón, Bernhard A. Huber

**Affiliations:** 1 Alexander Koenig Research Museum of Zoology, Adenauerallee 160, 53113 Bonn, Germany; 2 Center for Macroecology, Evolution and Climate, Natural History Museum of Denmark, University of Copenhagen, Copenhagen, Denmark; 3 Natural History Museum, University of Oslo, PO Box 1172 Blindern, NO-0318 Oslo, Norway; 4 Department of Natural History, University Museum of Bergen, University of Bergen, PO Box 7800, NO-5020 Bergen, Norway; 5 Instituto de Biologia UNAM, sede Tlaxcala. Contiguo FES-Zaragoza Campus III, Ex Fábrica San Manuel de Morcom s/n, San Miguel Contla, Municipio de Santa Cruz Tlaxcala, C.P, 90640 Tlaxcala, Mexico

**Keywords:** Microhabitat, Diversification rates, Speciation, Leaf dwelling, Pholcidae, Phylogeny

## Abstract

**Background:**

Microhabitat changes are thought to be among the main drivers of diversification. However, this conclusion is mostly based on studies on vertebrates. Here, we investigate the influence of microhabitat on diversification rates in pholcid spiders (Araneae, Pholcidae). Diversification analyses were conducted in the framework of the largest molecular phylogeny of pholcid spiders to date based on three nuclear and three mitochondrial loci from 600 species representing more than 85% of the currently described pholcid genera.

**Results:**

Assessments of ancestral microhabitat revealed frequent evolutionary change. In particular, within the largest subfamily Pholcinae, numerous changes from near-ground habitats towards leaves and back were found. In general, taxa occupying leaves and large sheltered spaces had higher diversification rates than ground-dwelling taxa. Shifts in speciation rate were found in leaf- and space-dwelling taxa.

**Conclusions:**

Our analyses result in one of the most comprehensive phylogenies available for a major spider family and provide a framework for any subsequent studies of pholcid spider biology. Diversification analyses strongly suggest that microhabitat is an important factor influencing diversification patterns in pholcid spiders.

**Electronic supplementary material:**

The online version of this article (10.1186/s12862-018-1244-8) contains supplementary material, which is available to authorized users.

## Background

Species numbers differ vastly among groups of organisms – a phenomenon observed at any taxonomic level. Differences in species richness of clades of different age are sometimes explained by the longer time that older clades had to accumulate species (e.g. [1, 2]). However, sister clades which are of the same age per definition often differ substantially in species richness. Therefore, net diversification rates (speciation minus extinction rates) must differ even among closely related groups. Indeed, it was recently suggested that diversification rates may explain most variation in species richness among organisms [3].

A range of factors that potentially affect rates of diversification are known. Climate and in particular changes of climatic niches among species are thought to be among the main causes of diversification rate differences [4–9]. On the macro-ecological level, invasions into new adaptive zones play a major role and have promoted some of the largest radiations. So is the diversity of many phytophagous insect lineages likely triggered by the rise of angiosperms in the Cretaceous [10–12]. Further factors that may affect rates of diversification are differences in body size and size dimorphism [13, 14], sexual selection [15–18], diet [19], habitat [20, 21], and parasitism [21]. The total rate of species production is highest in tropical biomes – either caused by increased speciation rates [22] or simply by the vast number of species that are already present there [23]. Higher rates in the tropics may be caused by increased opportunities for the evolution of reproductive isolation, faster molecular evolution, or the increased importance of biotic interactions [24].

Recently, microhabitat has been suggested as one of the most important factors that drive variation in diversification rates among vertebrates [20, 25–27]. Its effect may even supersede that of climatic niche [8], often changing several times within evolutionary young taxa [28]. It has been proposed that traits like microhabitat that are involved in local-scale resource use (alpha niche) may be more important in explaining patterns of diversification than those related to the broad-scale distribution of species (beta niche), as suggested in analyses across vertebrates and oribatid mites [25, 29, 30]. This might be because alpha-niche traits primarily change over deeper time scales while beta-niche traits (e.g., climate preferences) frequently change on lower time scales, which was shown for amphibians, reptiles, and birds [6, 29, 31–33].

**Fig. 1:**
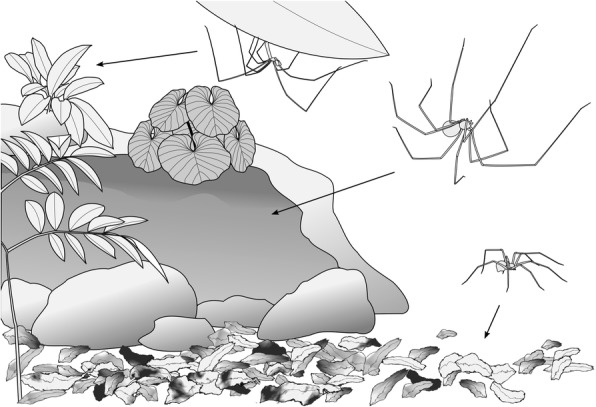
Microhabitats. Schematic drawing of the three main types of microhabitat (leaf, space, ground) that pholcid spiders inhabit, and of exemplary representatives

Web spiders are generally stationary and specimens are predominantly hand collected. Thus, in contrast to many other groups of invertebrates, information on the microhabitat of pholcid spiders (Araneae: Pholcidae) is available for a large percentage of species. This makes them ideal candidates for the investigation of the relationship between microhabitat and diversification rate. Three main types of microhabitat can be distinguished in pholcids (Fig. 1): (i) ground, i.e. leaf litter and under objects on the ground; (ii) space, i.e. sheltered spaces such as among tree buttresses, rocks, and logs; and (iii) leaf, i.e. the lower surface of live leaves [34–36]. Pholcid spiders, commonly known as daddy-longlegs spiders, have a worldwide distribution from ca 56° N to 42° S, from sea level to 3800 m, and from deserts to tropical forests [36–38]. These small to medium-sized spiders are well-known because of several synanthropic species but the vast majority of species is found in tropical forests where they are often among the most abundant and diverse web-building spiders [36, 39–42]. With currently more than 1600 described species, pholcids are among the most species-rich spider families [43]. Previous studies on pholcid phylogenetics [44–49] indicate that microhabitat might frequently have changed in the evolutionary history of the group, probably with numerous convergent origins of leaf dwelling. Putative sister groups often differ dramatically in species numbers, suggesting variation in net diversification rates.

In the present study we inferred the evolutionary history and plasticity of pholcid spiders’ microhabitats using a newly developed molecular phylogeny based on three nuclear and three mitochondrial DNA markers. Compared to previous studies, we extended the taxon sampling to include 600 species representing more than 85% of described pholcid genera. We also collected microhabitat information first hand for 88% of the examined species. Separate analyses of leg proportions as a proxy for microhabitat allowed a near-complete species coverage. We investigated the evolutionary plasticity of microhabitats by ancestral state reconstructions. Using current species numbers and estimates of extant diversity, we analyzed diversification rates in pholcids and tested the effect of microhabitat on diversification dynamics.

## Methods

### Sampling and molecular lab procedures

The taxon sampling for the phylogenetic analyses is based on previously published phylogenies of the group [9, 36, 39, 44–47, 50–53] and aims to include as many species as possible to reduce phylogenetic error [54, 55] and minimize biases of macroevolutionary inferences [56–58]. The final dataset included 635 pholcid terminals representing 600 pholcid species from all major lineages, covering more than 85% of the described genera and 38% of the described species. Of these, 391 species (423 specimens) were collected and sequenced as part of this study and data for additional 229 species were downloaded from GenBank (Additional file 1: Figure S1, Tables S1, S2). Thirty two outgroup species from GenBank were included based on Dimitrov et al. [45]. Previously missing loci were sequenced for 17 species.

Total genomic DNA was extracted from one to three legs, depending on the size of the specimen, and rarely from whole specimens using *Qiagen® DNeasy Blood & Tissue Kit*. The *Qiagen® Multiplex PCR Kit* was used to amplify partial sequences of three mitochondrial (12S rRNA, 16S rRNA, and cytochrome c oxidase subunit 1 [CO1]) and nuclear (18S rRNA, 28S rRNA, and histone 3 [H3]) loci each. 1.6 μl of each primer (Additional file 1, Table S3) and 1.2 to 2.5 μl undiluted DNA were used in 20 μl reaction mixes. The following protocols were used: hot start Taq activation: 15 min at 95 °C; 35 cycles á 35 s denaturation at 95 °C, 60 s annealing at 49 °C (12S, 16S) or 51 °C (18S, 28S, H3) and 60 s elongation at 72 °C; 10 min final elongation at 72 °C. A touch down program was applied for CO1, reducing the annealing temperature by 1° per cycle during the first 15 cycles, starting at 55 °C, and subsequent 25 cycles at 50 °C annealing temperature and 90 s elongation time. PCR products were purified using *Qiagen® QIAquick PCR purification* kits or 3 M sodium acetate precipitation. Samples were sent to Macrogen (Amsterdam, Netherlands) for forward and reverse Sanger sequencing and edited manually in Geneious v. 7.1.8 (Biomatters; available from [59]). Primers were cut from the sequences prior to multiple sequence alignment. Contaminations were identified by BLAST searches against the GenBank nucleotide data collection and by the help of preliminary gene trees. Since repeated amplification of single genomic DNA extracts for 28S yielded varying products depending on the PCR program used, suspicious assemblies of species in the corresponding gene trees were evaluated for potential paralog copies of the locus. If such assemblies split apart species from several morphologically well-supported species groups and were not recovered by other loci, the sequences with the conflicting signal were discarded.

### Phylogenetic inference

We applied the divide-and-conquer realignment technique implemented in SATé-II 2.2.7 [60] which improves multiple sequence alignment particularly when highly variable regions are included. In several iterations, the data are deconstructed to smaller subsets of related specimens (subproblems) which are subsequently merged. A phylogenetic tree based on all loci is simultaneously inferred guiding the alignment of each locus. The break strategy was set to ‘centroid’ to create subproblems with a maximum size of 100 taxa which were aligned with MAFFT-linsi v. 7.299b [61, 62] and subsequently merged with MUSCLE v. 3.7 [63, 64]. Searches for alignment guide trees were done with RAxML v. 8.2.9 [65] on the partitioned supermatrix. Five more iterations were done after SATé failed to find a tree/alignment pair with a higher likelihood score than in the previous iteration. Alignments were manually checked for reverse complement sequences, stop codons, and obvious errors in Aliview v. 1.18.1 [66].

In order to reduce the amount of missing data, we included 52 chimera taxa (Additional file 1, Table S4). Most of these (48) originated from specimens from the same sampling event (same vial). In four cases, specimens originated from geographically close localities and preliminary analyses indicated a very close relationship. Although some loci had large amounts of missing data, they were included in the analyses since their exclusion may reduce phylogenetic accuracy [67]. This applies in particular to conservative genes like 18S and 28S that might bear information on deeper nodes. In addition to the complete dataset, a dataset with reduced missing data was compiled that included specimens having at least 4 markers successfully sequenced. A third dataset was created by the exclusion of rogue taxa. Rogue taxa can affect phylogenetic inference by having an unstable position in the tree due to ambiguous or insufficient phylogenetic signal [68–70]. We ran multiple iterations of RogueNaRok [68] using the web service [71] until no more rogue taxa were found. In each iteration, rapid bootstrap supports [72] from 1000 iterations were maximized for a maximum likelihood tree inferred by RAxML v. 8.2.9 [65] based on reduced data from the previous iteration (GTRCAT model; data partitioned by loci). Optimal partition schemes and substitution models for subsequent thorough tree searches were inferred with PartitionFinder v. 2.1.1 [73–75] for all three datasets separately using a greedy search [75] and evaluating all available models of evolution.

Searches for the maximum likelihood tree were done multiple times [76, 77] with two different algorithms: RAxML v. 8.2.8 [65] and IQ-TREE v. 1.5.4 [76]. In RAxML, we conducted 100 replicates, each starting from a distinct parsimony tree using partitions based on PartitionFinder and using the GTRCAT model of sequence evolution. We refrained from estimating invariant sites since their inference may conflict with gamma categories inference [78]. We used 25 CAT-gamma categories which sufficiently cover sites with low variation, making an extra parameter superfluous [79]. IQ-TREE implements (partially) terrace-aware algorithm [77] which efficiently handles gappy alignments and may lead to trees with improved likelihood [77]. Models and partition schemes were again chosen based on PartitionFinder and 100 searches for the maximum likelihood tree were conducted, finally choosing the one with the highest likelihood.

Branch support was assessed with (i) 100 standard bootstrap replicates (SBS), (ii) 1000 rapid bootstrap replicates (RBS) [72], (iii) Shimodaira-Hasegawa-like approximate likelihood ratio test (SH-like aLRT) [74] supports, and (iv) quartet sampling [80]. Requirements of SBS (e.g., site independence) are rarely met by empirical data and may be particularly problematic with many missing data [80–82]. SBS, RBS, and SH-like aLRT supports are known to underestimate the true probability of a clade to be correct, although RBS seems to have a tendency to be less conservative [83]. SBS ≥ 80, RBS ≥ 95, and SH-like aLRT supports ≥80 roughly correspond to a 95% probability for the clade to be correct and are thus considered to present reasonably good support; SH-like aLRT supports < 50 are not representative for true clade support [83]. SH-like aLRT supports are fast to compute but only evaluate alternative topologies around the branch of interest [74, 84] and can thus be interpreted as local supports. Recently published measures of branch support based on quartet sampling [80] are less affected by missing data. Four statistics, i.e., quartet concordance (QC), quartet differential (QD), quartet uncertainty (QU), and quartet fidelity (QF) are calculated, which measure overall branch support (QC), the potential presence of alternative evolutionary histories (QD), data information content (QU), and individual taxa tendency to produce alternative topologies (similar to rogue taxa; QF). Its ability to distinguish between lack of information and conflicting signal as causes for low branch support offers more comprehensive and specific information on branch support.

All RAxML, IQ-TREE, PartitionFinder, and quartet sampling analyses were conducted on the computing cluster of the Zoological Research Museum A. Koenig.

### Molecular dating

Diversification analyses depend on the branching pattern inferred by time calibration of the phylogeny. We therefore applied three different dating approaches: non-parametric rate smoothing implemented in treePL v. 1.0 [85], Bayesian relaxed-clock dating using MCMCtree v. 4.9e [86], and RelTime, a fast ad hoc approach implemented in MEGA v. 7.0.20 [87–89]. All methods were applied to the best maximum likelihood (ML)-tree for the complete dataset without changing the topology.

Calibration points were adopted from Dimitrov et al. [45] without using the fossils for *Quamtana* and Nephilidae, since their identity or phylogenetic position has been contested [90, 91]. Stem ages were calibrated with minimum fossil ages (Additional file 1, Table S5). The Macaronesian clade of *Pholcus* was calibrated with a maximum age of 14 My [45, 92]. Fossil age uncertainty was implemented in MCMCtree using heavy tailed Cauchy distributions.

For treePL, a ‘prime’ analysis with smoothing = 1 was done to assess the best optimization method using ‘thorough’ estimation. Cross-validation was used to estimate the best fitting smoothing value (by 10 iterations in a range from 1000 to 0.000001). The smoothing value affects how strong rate variation among branches is penalized. Final analyses were done with ‘thorough’ optimization and increased number of penalized likelihood total optimization iterations (5, default = 2) and increased number of penalized likelihood simulated annealing iterations (10,000, default = 5000).

RelTime [87] is a very fast method which was originally intended for the estimation of relative divergence times in large phylogenies, but can also assess absolute times. It was shown to outperform other non-Bayesian methods when high rate increases in specific clades are present [87]. However, a recent study revealed shortcomings of RelTime in relaxing the clock among internal branches of specific datasets, arguably because it essentially does infer divergence times under a strict clock [93]. Results were thus checked for loss in variation of relative branch rates at deeper node ages. We estimated ‘all clocks’ under the GTR-Γ model, using all sites.

MCMCtree uses an approximation to speed up likelihood calculations and thus outperforms BEAST in terms of speed. We used the independent rate model to avoid unrealistic rates [94–96]. The birth-death tree prior was set to a uniform distribution of nodes (*BDparas* = 1 1 0). The locus rate prior *(rgene_gamma)* was set to a Dirichlet, hence posterior time estimates are insensitive to the rate prior [97]. We set a diffuse gamma distribution G(1, 7) with mean 0.14, which was derived from the average pairwise genetic distances between the six loci of two distant species (*Gertschiola macrostyla* (S434) and *Holocnemus caudatus* (S435)), assuming a divergence time of about 210 Mya [45]. The prior for *σ*^*2*^ was set to G(1, 10), indicating serious violation of the strict clock [98]. The Markov Chain Monte Carlo (MCMC) analysis was run on the ZFMK computing cluster for 2e^5^ generations, sampling every 20 generations after a burnin phase of 2e^4^ generations.

Data and trees were plotted with ggplot2 [99] and ggtree v. 1.9.2 [100], respectively. Efforts to infer divergence times with the widely used BEAST software [101] were unsuccessful due to lack of convergence of the MCMC chain.

### Diversification analyses

To reduce the bias introduced by unequal sampling of clades, diversification analyses (Additional file 1, Figure S2) were conducted based (1) on the number of currently described species and (2) on an estimate of actual species richness. For the latter, species numbers of 102 taxonomic entities (Additional file 1, Table S6; mostly species groups, genera, or groups of genera) were updated by adding undescribed species available in collections and by accounting for obviously misplaced species and then multiplied by 2 or 3 depending on their assignment to one of two categories: (i) taxa from temperate regions, with limited distribution, focused collection, low endemism, easy to find (multiplied by 2); (ii) taxa from tropical regions, with wide distribution, large sampling gaps, high local endemism, difficult to collect (multiplied by 3).

To evaluate the influence of microhabitat on diversification (speciation + extinction) rates, each species was assigned to one main habitat type, i.e., “ground”, “leaf”, or “space” (represented by 206, 174, and 178 specimens, respectively) based on direct field observations. Since this information was available for only 88% of the species, the usage of the metatarsus to tibia ratio of the first leg as a proxy of microhabitat was evaluated. The correlation of this ratio with microhabitat was tested using a phylogenetic generalized least-squares analysis [102] as implemented in the R [103] package ape v. 4.1 [104] in conjunction with nlme v. 3.1–128 [105]. Fits of Ornstein-Uhlenbeck [106, 107] and Brownian Motion [108, 109] models of trait evolution were evaluated. Ancestral microhabitats were estimated by maximum likelihood using the ape-function ace and the underlying expm-package [110] and by maximum parsimony (function MPR in ape). Blomberg’s K [111] and Pagel’s lambda [112] were calculated as measures of phylogenetic signal of the metatarsus to tibia ratio of the first leg. All diversification analyses were done on the dated trees inferred with different methods. Outgroups and duplicate species were pruned from the trees.

Dependence of diversification rates on microhabitat was assessed with the diversitree R-package [113]. Multiple State Speciation and Extinction (MuSSE) was used for direct inference of diversification rates in dependence of microhabitats. Species with missing data were pruned from the tree prior to the analyses and the sampling fraction was set according to the above estimates. Traits were assumed to be sampled representatively, i.e., proportions of unsampled species’ character states were set according to sampled species. The models include speciation and extinction rate parameters per character state and character state transversion rates. Increasingly general models were evaluated against a constrained base model (Table 1). Character state transversion rates were always constrained to be equal. The examined species originate from several different biomes, which might confound trait dependent diversification rate analyses if, e.g., species from tropical biomes had higher speciation rates. Therefore, we also tested the influence of biomes on speciation rates. Additionally, to exclude potential confounding effects of biome on diversification rates in different microhabitats, we conducted an analysis with tropical broadleaf forest species alone, which was possible because they constitute the majority of total species. Species’ biomes were inferred by overlaying all available species’ sampling points from the senior author’s database with the biomes map from Olson et al. [114] in QGIS v. 2.18.10 [115] using the NNJoin plugin v. 3.0.3 [116]. Each species was assigned to the biome that contained the majority of its sampling points. Twenty-three species with ambiguous biomes and ten synanthropic species were not considered in this analysis (Additional file 1, TableS2). A potentially confounding effect of unobserved (hidden) traits on diversification rates was evaluated with HiSSE [117]. Since HiSSE operates on binary trait data, leaf- and space-dwelling species were pooled and compared to ground living species. Models of increasing complexity were evaluated against a base model with equal turnover rates (speciation + extinction) and equal extinction fractions (extinction / speciation) and no hidden state (Additional file 1, Table S7).

**Table 1:** Models of diversification rates that were used with MuSSE. All models were evaluated for all dated trees (MCMCtree, treePL, and RelTime), choosing the best fitting one by the AIC value

model	speciation rates	extinction rates	setting
1	λ_123_	μ_123_	no difference in speciation or extinction rates between microhabitats
2a	λ_1_, λ_2_, λ_3_	μ_123_	speciation rates differ among microhabitats
2b	λ_1_, λ_23_	μ_123_	no difference in speciation rate of leaf and space, but difference to ground
3	λ_123_	μ_1_, μ_2_, μ_3_	extinction rates differ among microhabitats
4	λ_1_, λ_2_, λ_3_	μ_1_, μ_2_, μ_3_	speciation and extinction rates differ among microhabitats

Data for the calculation of the leg ratio were available for 91% of the species. Using the leg ratio as a proxy for habitat preference, speciation rates were estimated as a function of this ratio using QuaSSE [118]. Linear, sigmoidal, and hump shaped speciation functions were evaluated with constant extinction rate. All models were estimated with and without the drift parameter, which describes the directional component of character evolution due to selection or any other within-lineage process that has a directional tendency [118].

Additionally, shifts in speciation rates were inferred with BAMM v. 2.5.0 [14, 119–121], using the currently described and the estimated species numbers for the calculation of clade-specific sampling fractions. Because of the tree size, 50 speciation rate shifts were expected a priori; other prior values were set using BAMMtools v. 2.1.6 [122]. Rate shifts were allowed to occur in clades with a minimum size of two taxa. The Metropolis- coupled MCMC chain was run for 10 Mio generations, sampling every 1000 generations after a burnin of 20%. BAMMtools was used to visualize the results.

## Results

### Phylogenetic inference

**Fig. 2:**
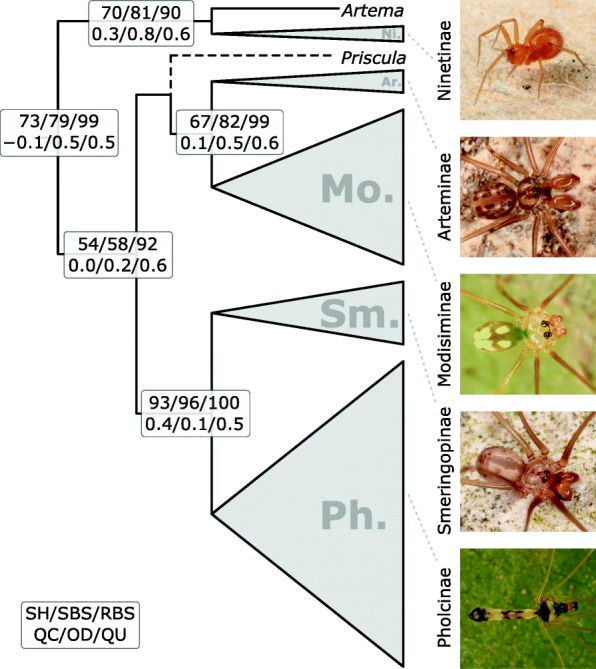
Pholcid subfamilies. Summary tree of pholcid subfamilies and their relationships based on the topologies inferred in all phylogenetic analyses. The genus *Priscula,* was sister of Modisiminae in some of the trees. Branch support values are SH-like aLRT supports (SH), standard (SBS) and rapid (RBS) bootstrap values, and quartet sampling measures (see inset)

Multiple sequence alignment resulted in a matrix of 3740 base pairs. PartitionFinder inferred an optimal scheme of one partition per locus, each with the GTR + Γ + I model of nucleotide substitution. For the complete dataset, RAxML found the tree with the highest likelihood. This tree was therefore used for subsequent analyses. Morphologically well-defined groups that were also used for species number estimations (Additional file 1: Table S6, Figure S3, S5), were largely recovered with good branch support. A detailed evaluation of systematic results and potential taxonomical consequences is beyond the scope of the present study and is the focus of a parallel paper [123]. Here, only subfamily relationships are presented (Fig. 2), which were concordant among analyses of the complete dataset with RAxML and IQ-TREE and the reduced datasets (minimum four loci and RogueNaRok). An exception was the genus *Priscula*, which took the sister position to Arteminae + Modisiminae in the RAxML inference of the complete dataset, while it was basal in Modisiminae in all other trees (see [123] for further details). The stability of subfamily relationships was mostly confirmed by reasonably high support values; however, the sister group relationship between [Arteminae + Modisiminae] and [Smeringopinae + Pholcinae] did not receive high support (Fig. 2, Additional file 1: Figure S3 – S8). A notable discordance for this node was observed between SH-like aLRT supports (SH), standard non-parametric bootstrap (SBS), and rapid bootstrap (RBS), with the latter being distinctly higher. Similar patterns were observed in several deeper nodes like for example in ancestral nodes of Pholcinae or Modisiminae. Quartet sampling supports were reasonable for Pholcinae + Smeringopinae but low for other deep nodes (Fig. 2, Additional file 1: Figures S3 – S8). Quartet differential (QD) scores and quartet uncertainty (QU) scores (Additional file 1: Figure S9) suggested potential alternative topologies (at least for some taxa) and generally not very informative data for subfamily relationships (50–60% of the quartet sampling replicates were uninformative).

Cross validation of the smoothing parameter in treePL favored very small values (10^− 6^), indicating strong rate heterogeneity across the tree. Absolute time estimates conspicuously differed among the applied methods (Additional file 1: Figures S10 – S13).

### Diversification analyses

**Fig. 3:**
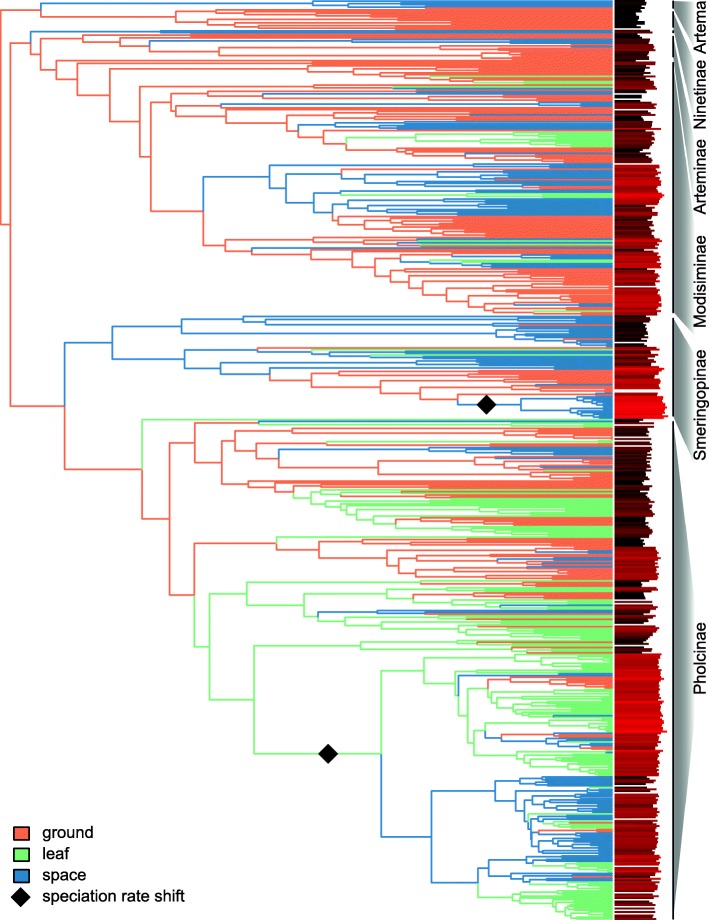
Ancestral microhabitat reconstruction. Time tree inferred with treePL with ancestral states inferred by maximum likelihood. Branch colors code the most likely ancestral microhabitat state. Bars next to tips illustrate the ratio of metatarsus to tibia of the first leg which was used as a proxy of microhabitat. Higher values are lighter red. Diamonds show speciation rate shifts of the best fitting scenario inferred with BAMM

Both maximum likelihood (ML) and maximum parsimony (MP) ancestral state reconstruction suggest frequent transitions of microhabitats in the evolutionary history of pholcids (92 based on maximum likelihood estimates of ancestral states for all dated trees; Fig. 3, Additional file 1: Figures S10 – S12). Despite ambiguity in the reconstruction of the ancestral microhabitat at the root of pholcid spiders, all methods rejected leaf dwelling as the ancestral state. A distinct and significant correlation was found between microhabitat and the ratio of metatarsus to tibia of the first leg. This finding was independent of time-calibration methods. Coefficient estimates for space living and leaf dwelling were similar and differed distinctly from coefficient estimates for ground living (Additional file 1: Table S8). Depending on whether the Akaike (AIC) or the Bayesian information criterion (BIC) was used for choosing the best fitting model of trait evolution, either the Ohrnstein–Uhlenbeck (OU) model or Brownian Motion was favored. However, the force stabilizing the ratio along the evolutionary history was always estimated to be small (OU model parameter α < 0.001) and thus the models always resembled Brownian motion. Nevertheless, a high phylogenetic signal of the ratio was inferred (Additional file 1: Table S9), suggesting a higher similarity of closely related species than expected under Brownian motion (i.e., phylogenetic niche conservatism [124]).

**Fig. 4:**
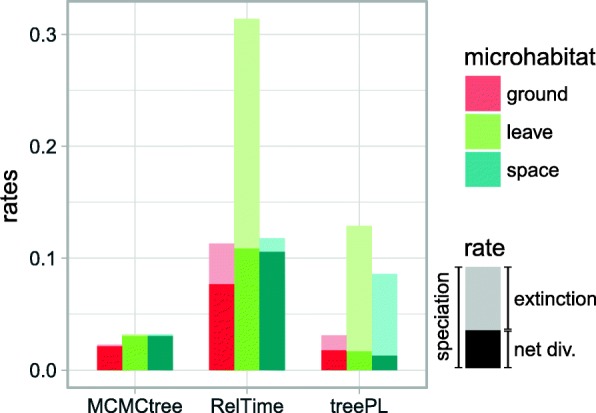
Microhabitat effect on diversification rates. MuSSE results for all dated trees. The rates are based on estimated species numbers to reduce bias by uneven taxonomic work published on different taxa (see main text)

Diversification rates were found to depend on microhabitat (*p* < 0.05; Fig. 4, Additional file 1: Tables S10–S12), irrespective of the underlying tree (i.e., treePL, MCMCtree, RelTime). Leaf dwelling species consistently showed higher speciation rates when compared to species from other microhabitats (sometimes equal to speciation rates in space living species). Rates based on estimated and currently described species numbers were largely concordant and did not alter main conclusions (Additional file 1: Figure S16, Tables S10–S12). Space living species had also increased speciation rates compared to ground living species, however this difference was less pronounced. Increased speciation rates were always accompanied by higher extinction rates. Nevertheless, net diversification (speciation minus extinction) was almost always increased in leaf dwellers and space living species (Fig. 4, Additional file 1: Figure S16). When using the ratio of metatarsus and tibia as a proxy for microhabitat, the results from QuaSSE also supported elevated speciation rate in species with higher leg-ratio related to “leaf” and “space” microhabitat habitat use (Additional file 1: Figure S18). Speciation and extinction rates among biomes also showed significant variation (Additional file 1: Figure S17). Diversification rates that were inferred for species from tropical broadleaf forest biome only, thus ruling out a confounding effect of biomes, were largely concordant with those based on all species (Additional file 1: Figure S17, Tables S11 – S12). HiSSE analyses, that test for the potential presence of other traits that influence diversification rates, were also largely concordant with findings from analyses that do not account for hidden traits, although net diversification rates in leave and space dwellers did not conspicuously supersede those of ground living species (Additional file 1: Figure S19, Tables S13 – S14). A major potential impact of a hidden trait compared to microhabitat was not found.

## Discussion

### Pholcid phylogeny

Phylogenetic relationships inferred in the present study largely confirm previous findings based on morphological and molecular data [36, 38–40, 44–47, 49, 53, 125–128]. Species groups that were previously identified based on morphological apomorphies were mostly recovered (Additional file 1: Table S6). However, several low support values and the presence of unstable taxa (whole clades or single rogue-species) lead to uncertainties, in particular in deeper relationships. Quartet sampling scores [80] suggest the presence of both data with low phylogenetic information content and conflicting signal (Additional file 1: Figure S9). The existence of paralog copies of the nuclear ribosomal array (including 18S and 28S rRNAs) may also act as a confounding factor. Paralogs of these gene arrays are also known from other arachnid groups [129–131], emphasizing the need for future phylogenomic scale datasets and approaches that explicitly address confounding factors and processes [132, 133]. A detailed systematic discussion will be published in a standalone article [123].

Due to the limited fossil evidence for the group and deviating estimates of divergence times across methods, estimates of lineage ages could not be proposed in the present study. A potential inability of RelTime to relax the molecular clock between internal branches [93] was not evident in our analysis.

### Evolutionary shifts of microhabitat

The present analyses with strongly increased species sampling corroborate indications from previous phylogenetic studies [45–47] that microhabitat frequently changes even among closely related species (Fig. 3, Additional file 1: Figures S10 – S12). Also the preference of the Brownian Motion model and low alpha parameter values of the Ohrnstein-Uhlenbeck model for microhabitat (PGLS regressions of microhabitat and leg ratio) indicate evolutionary instability of microhabitat use in pholcid spiders.

High phylogenetic signal of a trait might be interpreted as indication for the trait to change at deeper timescales [29]. Thus, the high phylogenetic signal in the leg-ratio approximation for microhabitat (Additional file 1: Table S9) might be interpreted as indication for less frequent change of microhabitat. Given the correlation of microhabitat and diversification rates, this would be in concordance with the idea that traits that differ at deep timescales may be more important for diversification [29]. However, regression analysis and plots of the distribution of leg-ratios clearly reveal increased values in leaf and space living taxa (Additional file 1: Figure S14, Table S8). Thus, the similarity in the ratio among leaf dwellers and space living species likely biases the phylogenetic signal calculations towards higher values since changes from space living to leaf dwelling and vice versa are not captured; i.e., the similarity of leaf dwellers and space living species artificially increase phylogenetic signal by increasing the probability of closely related species to resemble each other. Additionally, bimodalities were present in the ratio distributions of each microhabitat (Additional file 1: Figure S14). These were likely caused by different leg proportions among species with equal microhabitat preference in different phylogenetic lineages. Thus, similarity within a phylogenetic lineage is increased and phylogenetic signal further increases. The phylogenetic signal of the leg-ratio might thus overestimate phylogenetic signal of microhabitat preference.

### Increased diversification in leaf and space microhabitats

The present study suggests that microhabitat influences rates of diversification in pholcid spiders (Fig. 4). Despite the variance in absolute divergence times that we observed among methods (Additional file 1: Figure S13), relative estimates of diversification rates were largely concordant (Fig. 4, Additional file 1: Figure S16). Thus, their comparison among microhabitats is justified. In the context of microhabitat, accelerated diversification in pholcid spiders seems to be related to two factors: (i) frequent microhabitat change in a phylogenetic sense and (ii) leaf or space dwelling. Microhabitat change might facilitate the coexistence of many species on a local scale (e.g. by resource partitioning or intraguild predation escape [26, 134]) and thus explain its relation to diversification rates (Additional file 1: Figure S15). The causality of the observed relation between species numbers and microhabitat, however, remains subject to future studies. A leaf dwelling or space living lifestyle is associated with several factors that differentiate it from ground living conditions. Among those are prey availability and protection from predators which is also reflected in body color (leaf dwellers are pale whitish to green while ground dwellers tend to be brown). Leaf dwelling implies varying sizes and shapes of leaves that may require different webs [35] and consequently further differences in vibratory signal conduction, humidity, etc. Such factors might drive increased rates of speciation or reduce extinction [135, 136], e.g. by sexual selection, predator-prey interactions, or competition. Given the frequent change of microhabitat in the evolutionary history of pholcids, we do not expect that minor topological changes of the tree will alter the general conclusions of the present study.

Current methods to infer shifts in diversification rates [119, 137–139] are known to underestimate the number of speciation rate shifts on a phylogeny [119, 138]. The consistent inference of a speciation rate shift by BAMM in Pholcinae, where most shifts to leaf dwelling were observed, thus underlines the impact of leaf microhabitat on speciation rates. Inferences of ancestral microhabitat actually located a shift to leaf dwelling in close phylogenetic vicinity of the respective branch (Fig. 3, Additional file 1: Figures S10 – S12).

The inclusion of a world-wide sampling might confound estimates of speciation rates in microhabitats by potentially increased diversification rates in tropical areas [117]. Our data did not support higher diversification rates in tropical biomes since high speciation rates were for instance also found in the Mediterranean biome (Additional file 1: Figure S17). A confounding effect on the inference of diversification rates in microhabitats was excluded by analyzing species from tropical broadleaf forest only, from where the vast majority of species originated (Additional file 1: Figure S16). Exceedingly high extinction rates that were inferred for some biomes (Additional file 1: Figure S17) were likely caused by lacking statistical power since they were only covered by less than three species [58] and by the general difficulty of extinction rate inference from phylogenies of only extant taxa [58, 140–142].

## Conclusion

The present study reveals frequent evolutionary changes among pholcid spider microhabitats and explains the remarkable variation of the associated morphology (such as body size, leg proportions and color). While additional factors are likely to play a role in the diversification of pholcid spiders, the increase in net diversification rate in leaf dwelling but also in space living species emphasizes the importance of microhabitat for the evolution of high species richness. This is further supported by the observed shift in speciation rate in the subfamily Pholcinae that includes a large percentage of leaf dwelling taxa. In addition, our analysis of six molecular loci resulted in one of the most comprehensive phylogenies available for a major spider family and provide a framework for any subsequent studies of pholcid spider biology. Given the problems likely encountered due to multiple independently evolving nuclear ribosomal arrays in lycosid, jumping, and pholcid spiders, future phylogenetic studies should rely on genomic scale data, which allows to specifically address gene orthology. The general conclusions of the present study, however, are unlikely to be affected by minor topological changes in the presented phylogeny, and provide a strong argument favoring microhabitat as a major diversifying factor in pholcid spiders.

## Additional files

Additional file 1: (portable document format [.pdf]): Supplementary Figures S1 – S19 and supplementary Tables S1 – S14. (PDF 7729 kb)

Additional file 2: (comma separated value table [.csv]): All specimens’ leg-ratio and assigned microhabitat. The metatarsus to tibia ratio of the first leg and the assigned microhabitat (ground, leaf, or space) is given for all pholcid specimens included in the phylogeny. (CSV 10 kb)

## Abbreviations

12S: 16S, 18S, 28S: 12S, 16S, 18S, and 28S ribosomal RNA genes, respectively
AIC: Akaike information criterion
BIC: Bayesian information criterion
CO1: Cytochrome oxidase c subunit 1 gene
GTRCAT, GTR-Γ: General time reversible models of nucleotide substitution with CAT and gamma approximations of rate heterogeneity, respectively
H3: Histone 3 gene
ML: Maximum likelihood
MP: Maximum parsimony
PCR: Polymerase chain reaction
QC, QD, QF, QU: Support measures of the quartet sampling method (quartet concordance, quartet differential, quartet fidelity, and quartet uncertainty, respectively) [80]
RBS: Rapid bootstrap support [72]
SBS: Standard bootstrap support
SH-like aLRT: Shimodaira-Hasegawa-like approximate likelihood ratio test [74]
