# Supplementary material for: Microhabitat change drives diversification in pholcid spiders
Source: BMC Evol Biol. 2018 Sep 19;18:141. doi: 10.1186/s12862-018-1244-8 (PMC6145181; doi:10.1186/s12862-018-1244-8)
Supplement: Supplementary file 1 — (portable document format [.pdf]): Supplementary Figures S1 – S19 and supplementary Tables S1 – S14. (PDF 7729 kb) [file 12862_2018_1244_MOESM1_ESM.pdf]

1 Supplementary Figures

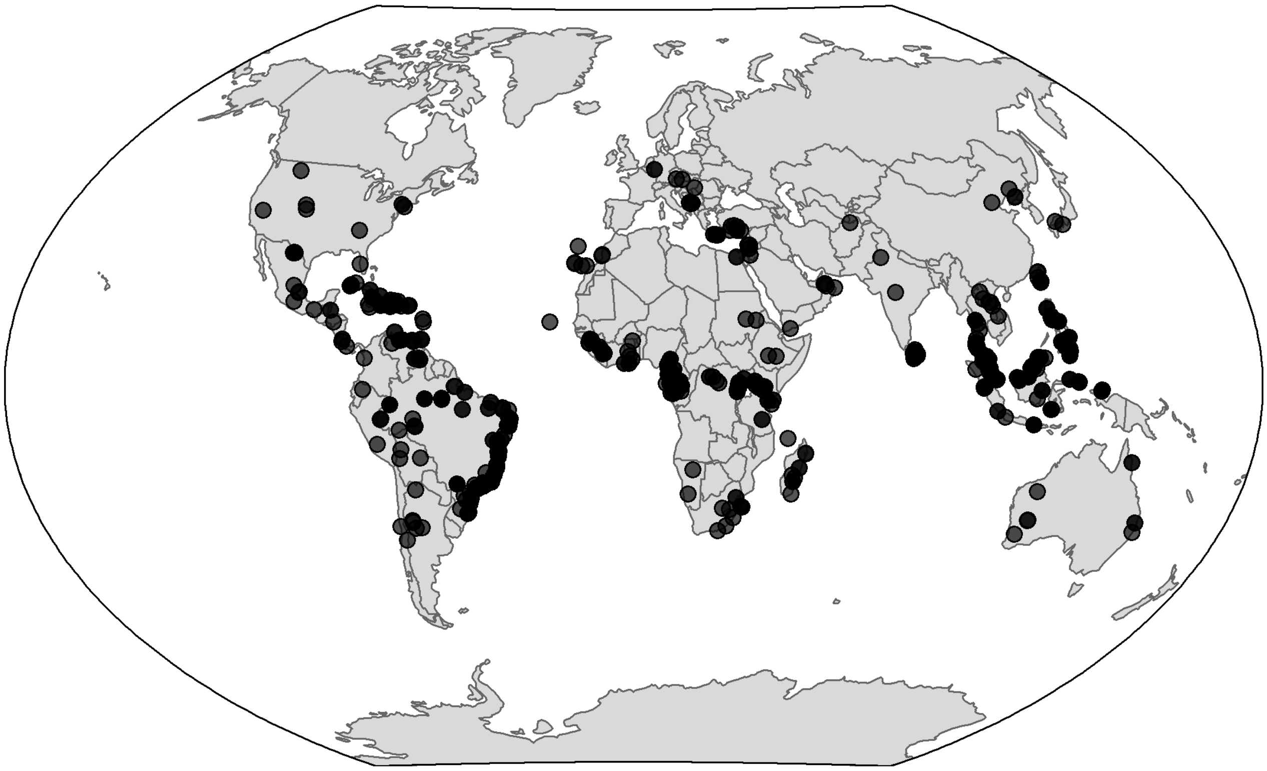

Figure S1: Map of sampling locations of all specimens included in the phylogenetic analyses.

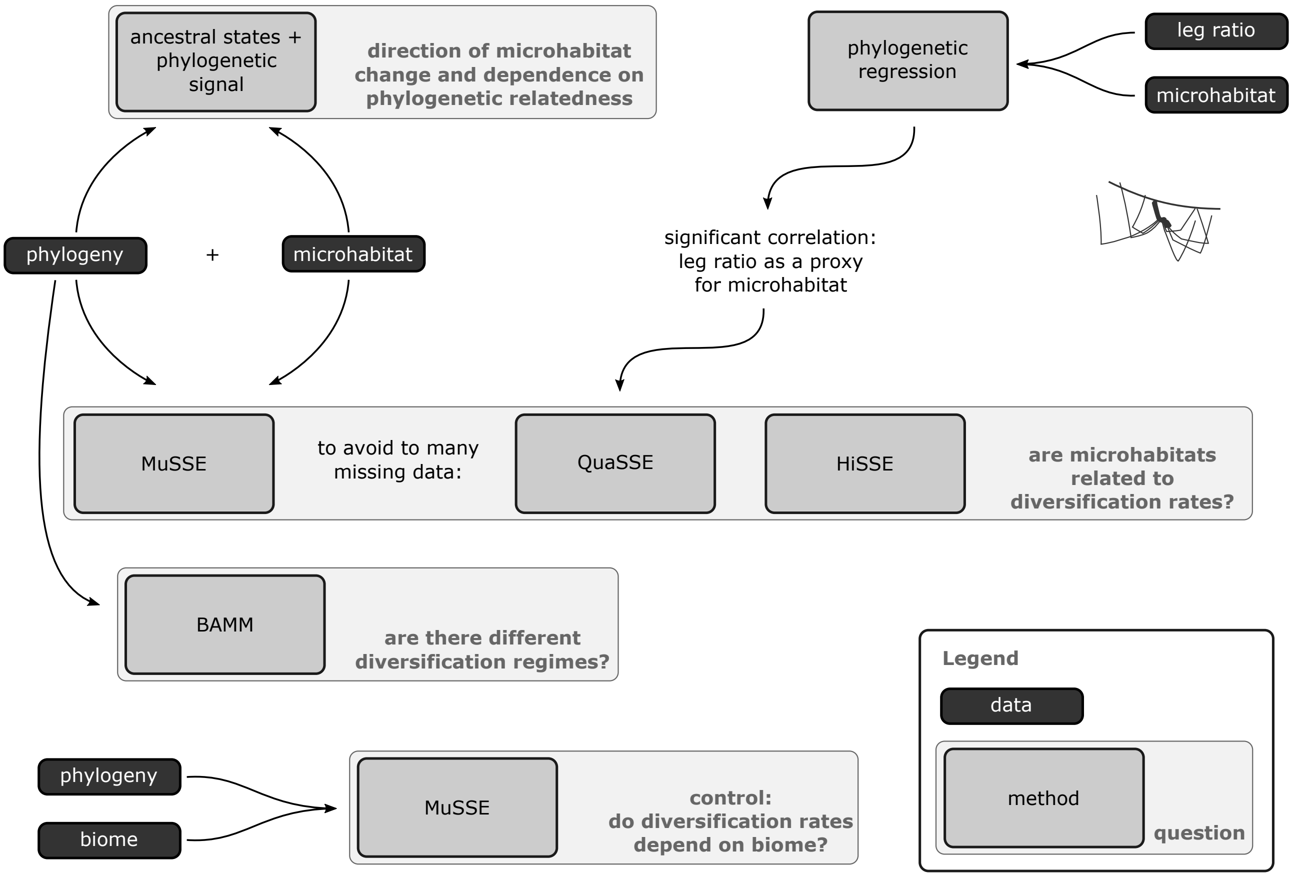

Figure S2: Workflow and overview of diversification analyses.

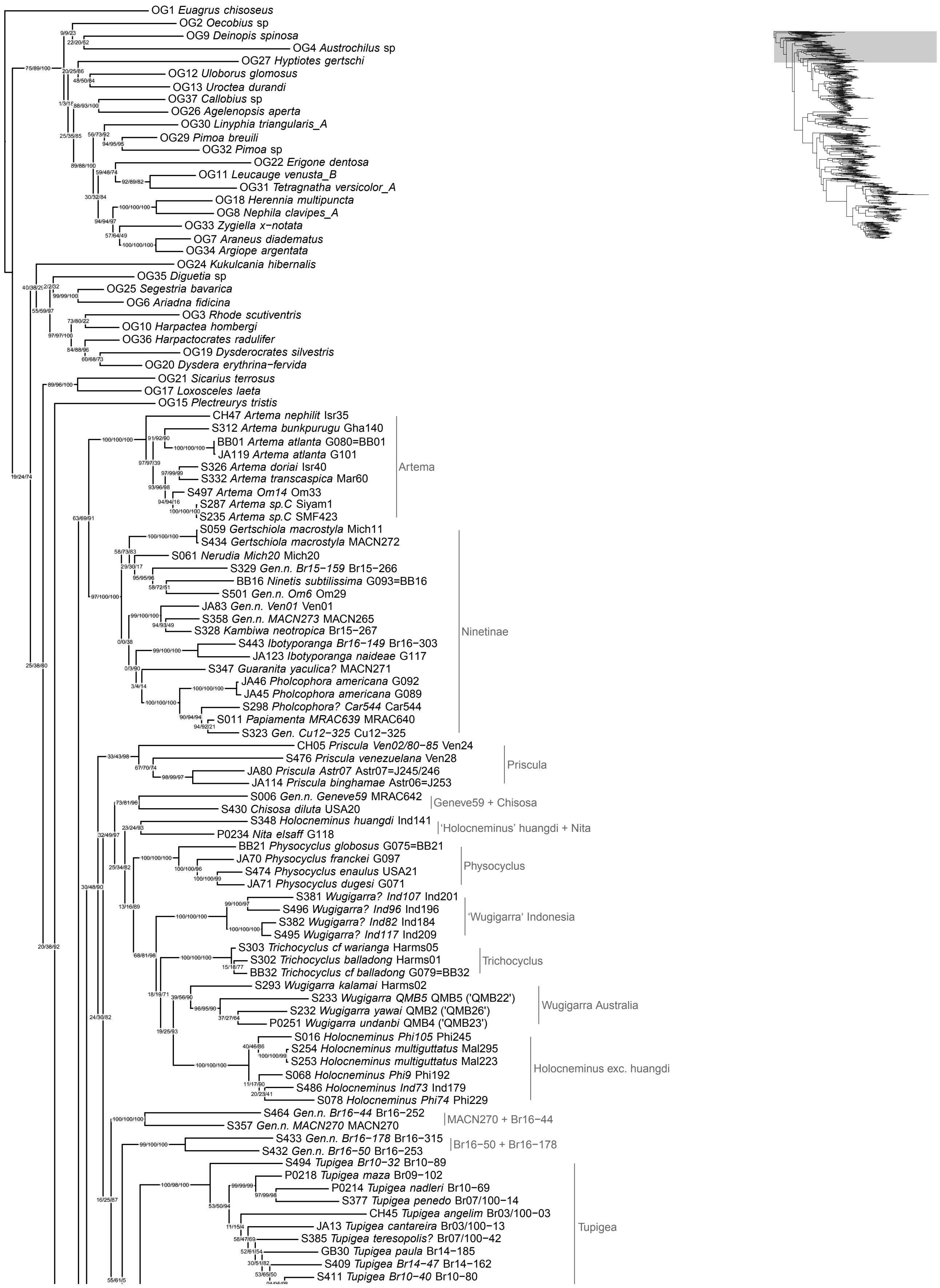

Figure S3 (a). Caption on page 9.

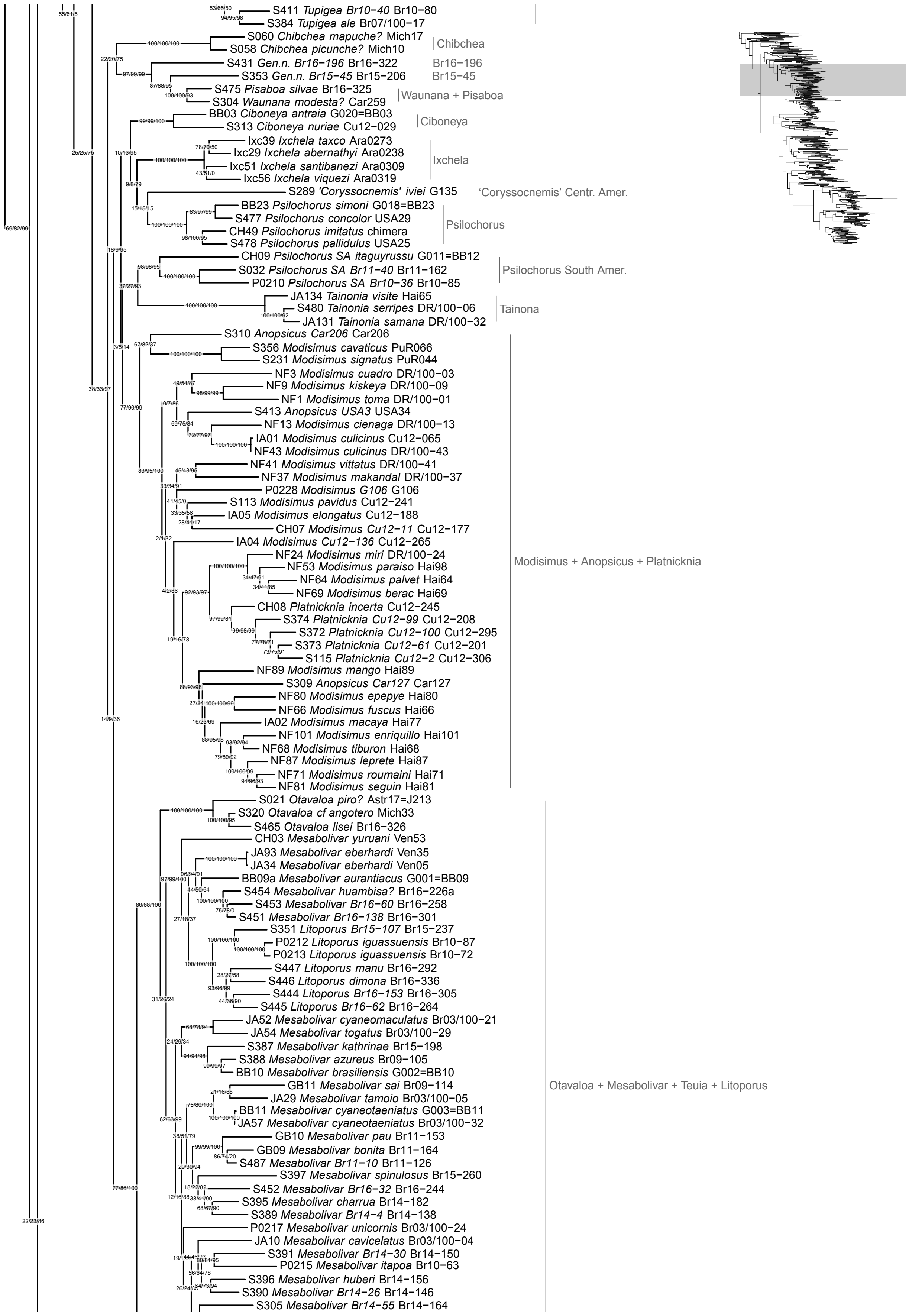

Figure S3 (b). Caption on page 9.

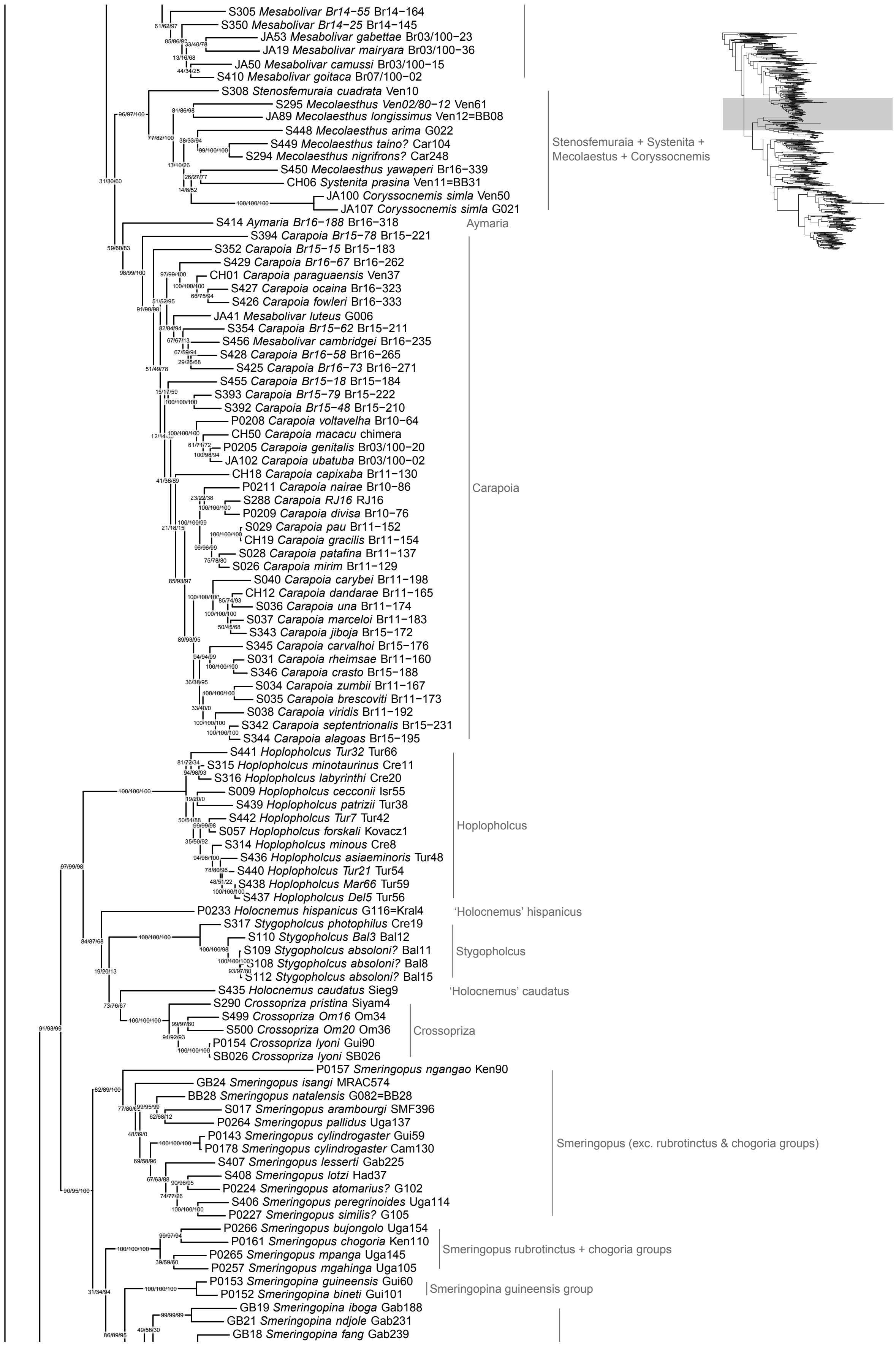

Figure S3 (c). Caption on page 9.

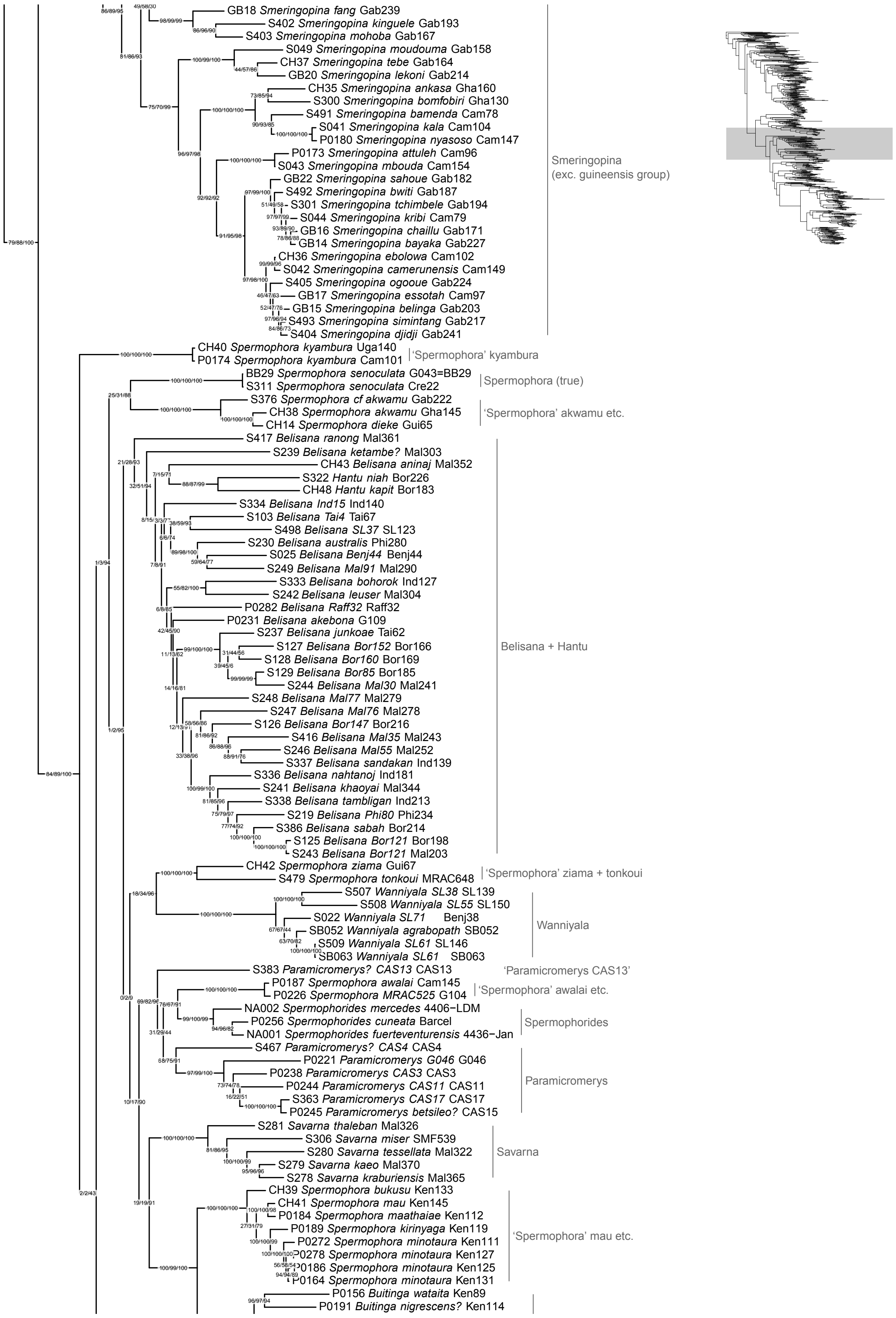

Figure S3 (d). Caption on page 9.

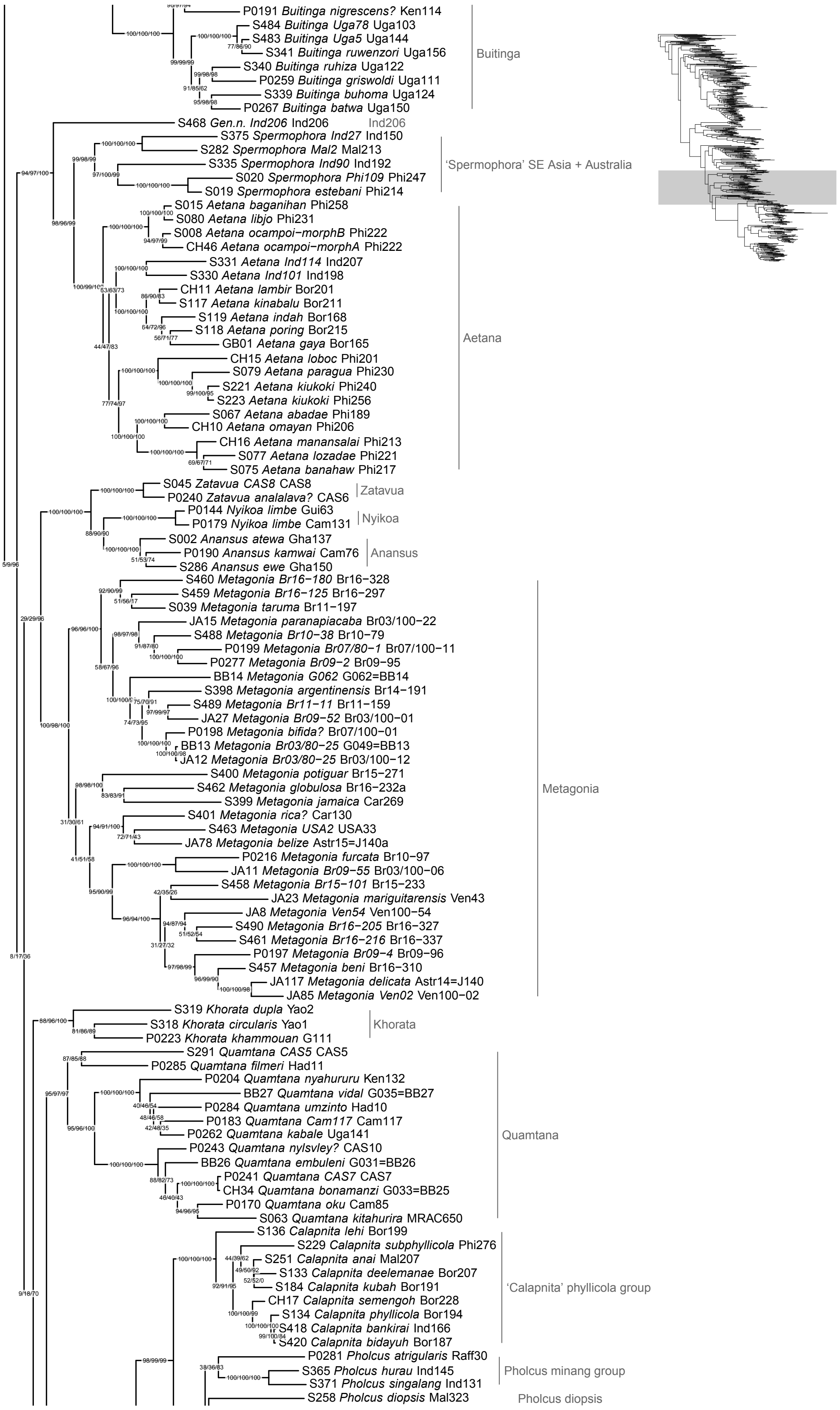

Figure S3 (e). Caption on page 9.

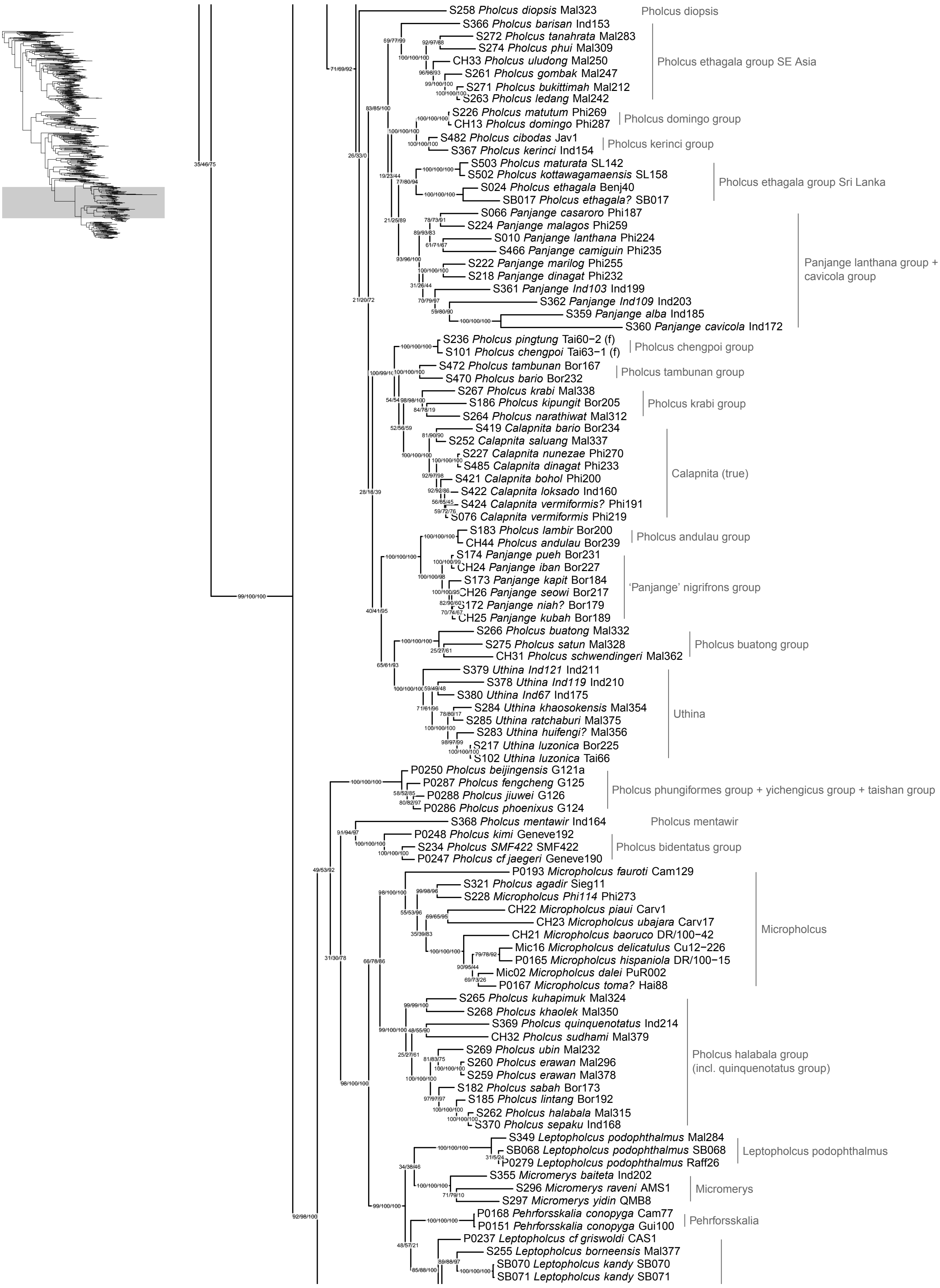

Figure S3 (f). Caption on page 9.

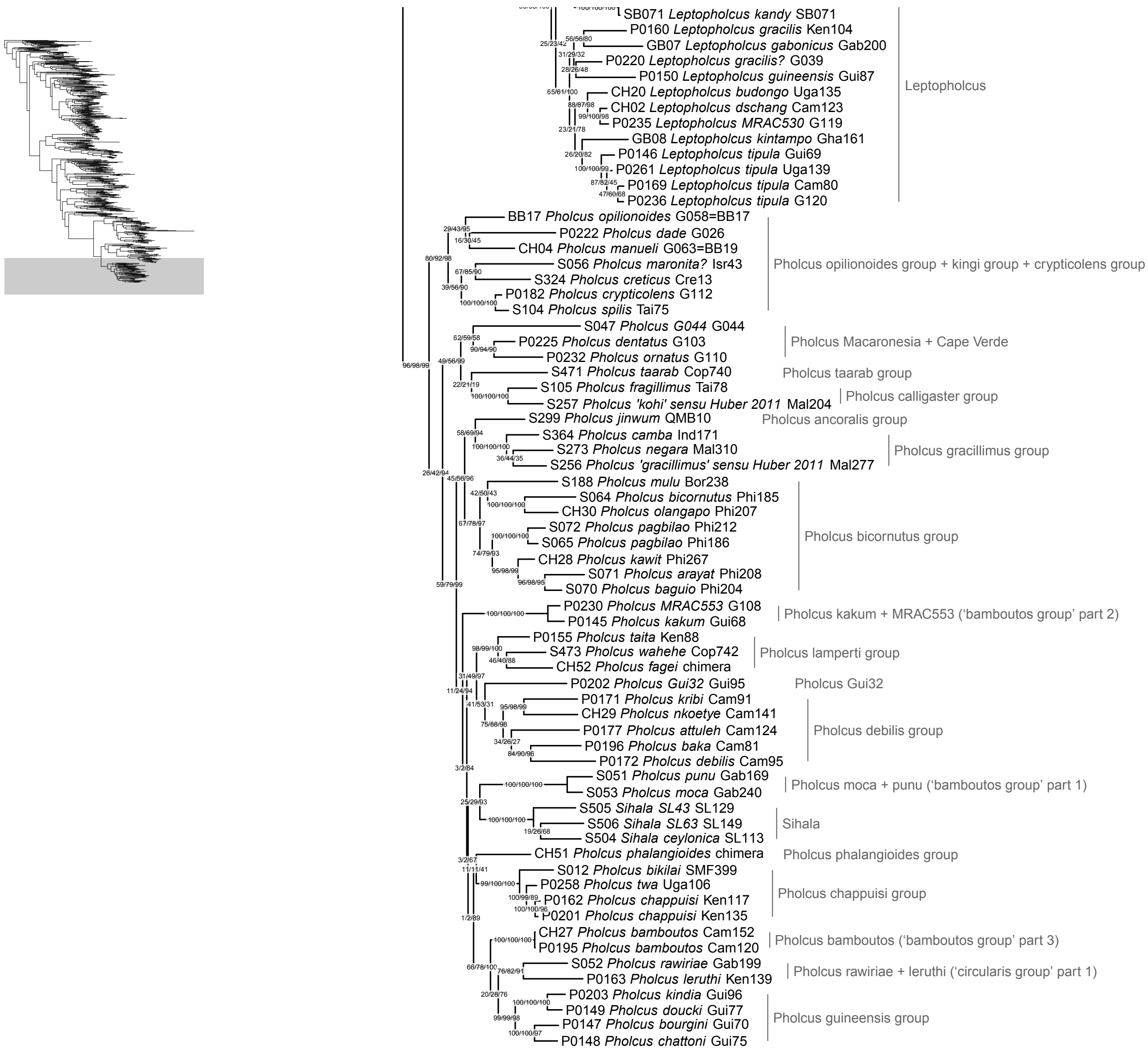

Figure S3 (g). Best maximum likelihood tree found by RAxML for the complete sampling. Branch support values are standard bootstrap, rapid bootstrap and SH-like aLRT values, respectively, separated by a slash. Tip labels are composed of the specimens' code, species name, and collection vial number.

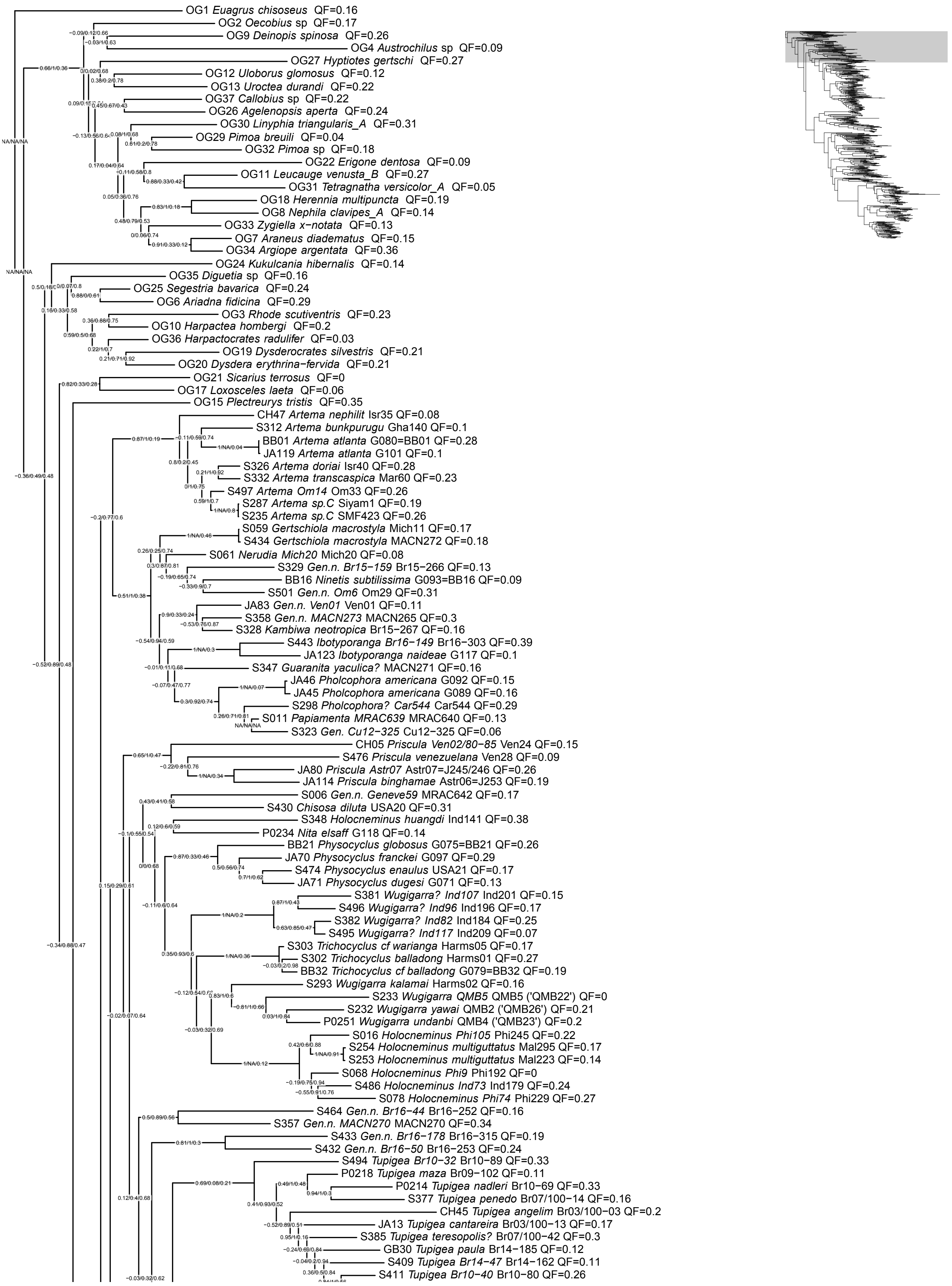

Figure S4 (a). Caption on page 16.

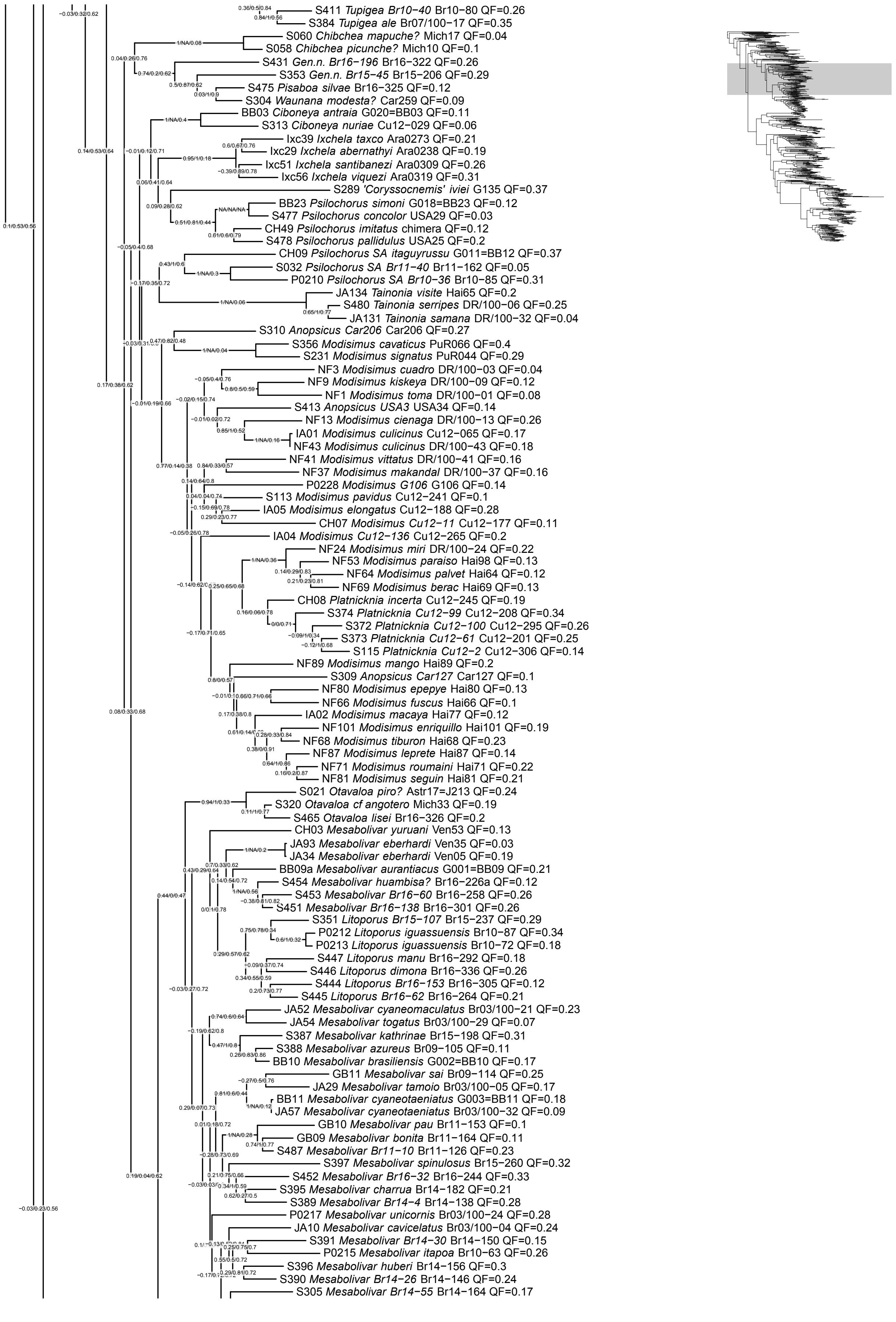

Figure S4 (b). Caption on page 16.

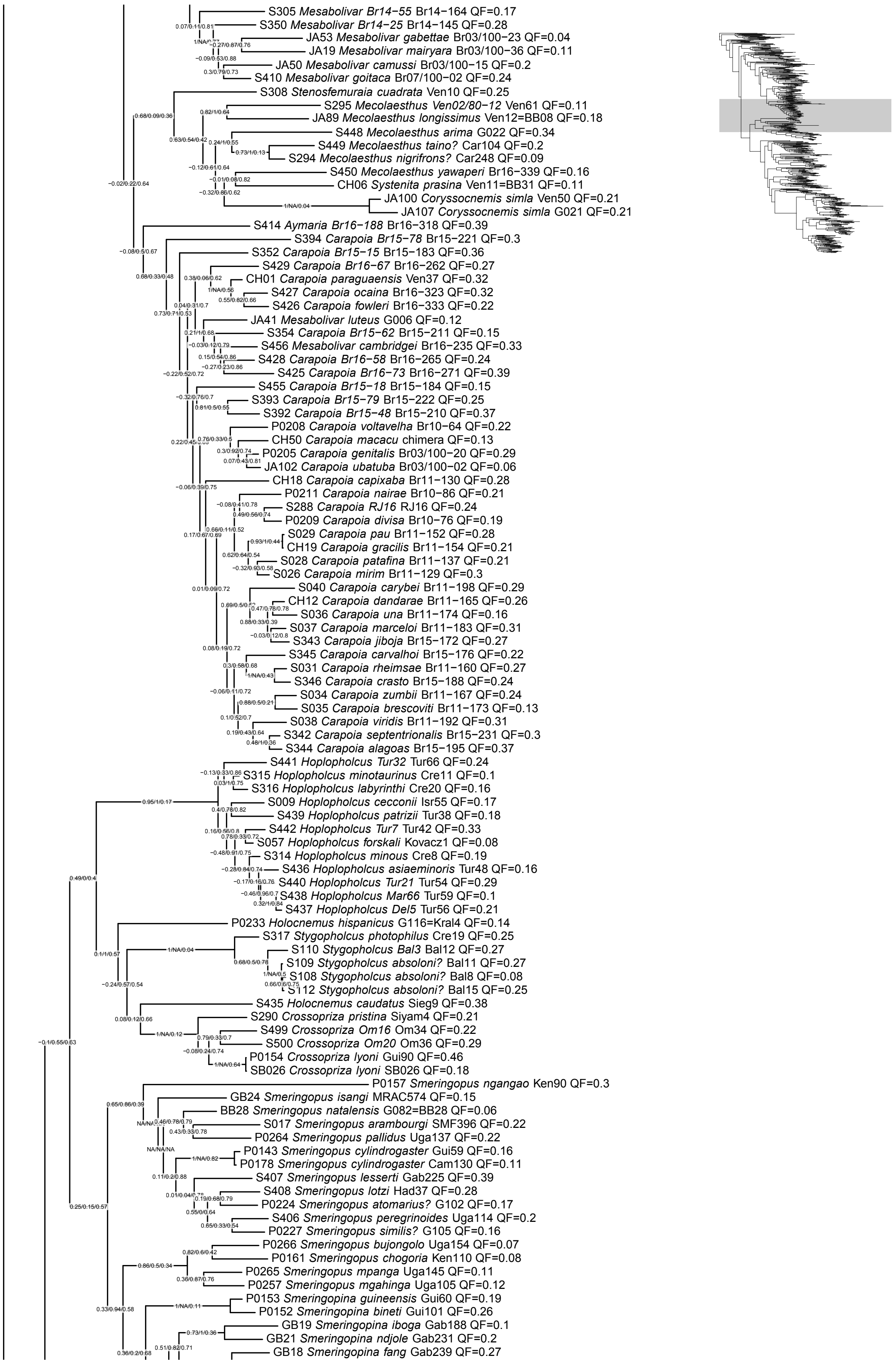

Figure S4 (c). Caption on page 16.

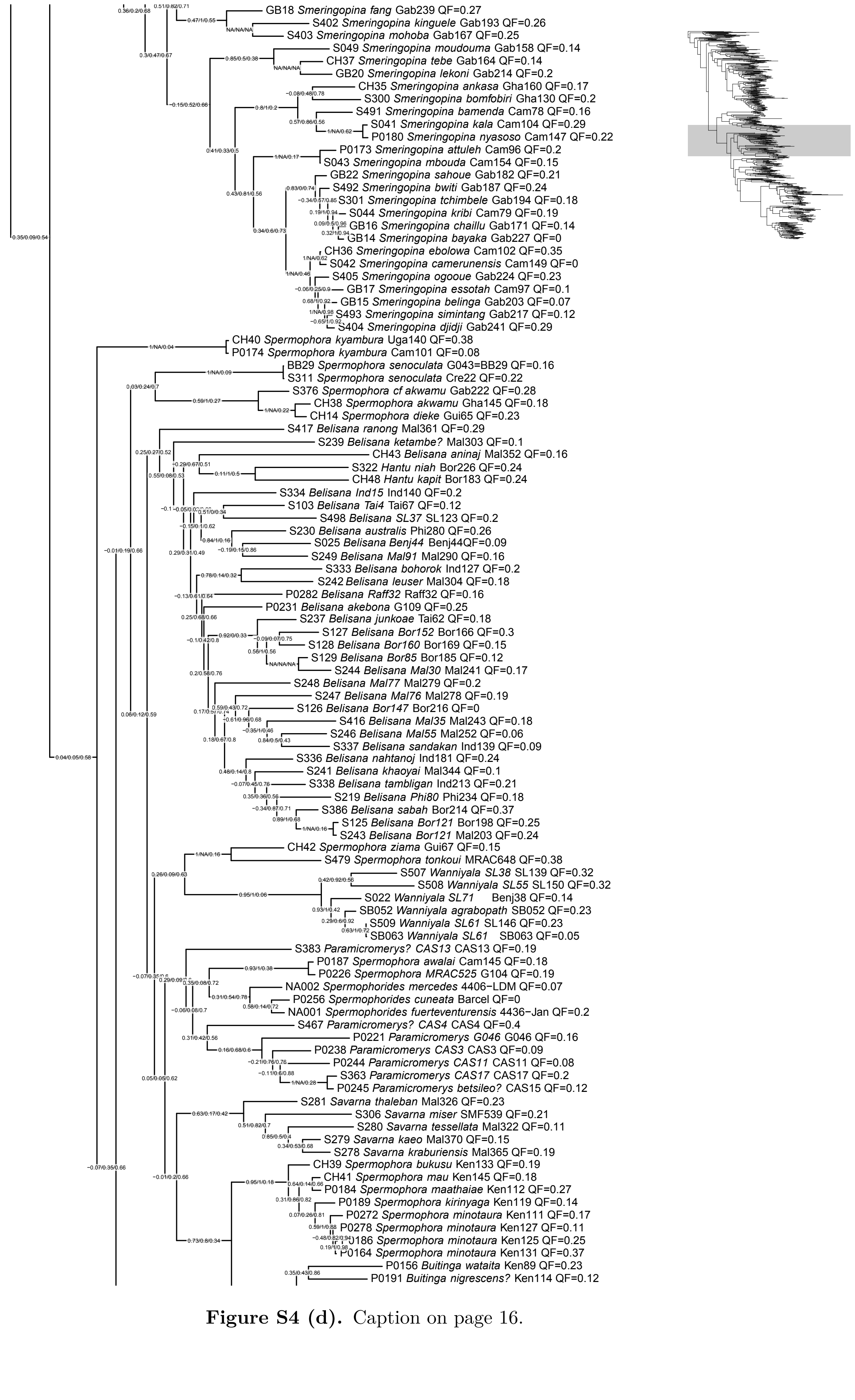

Figure S4 (d). Caption on page 16.

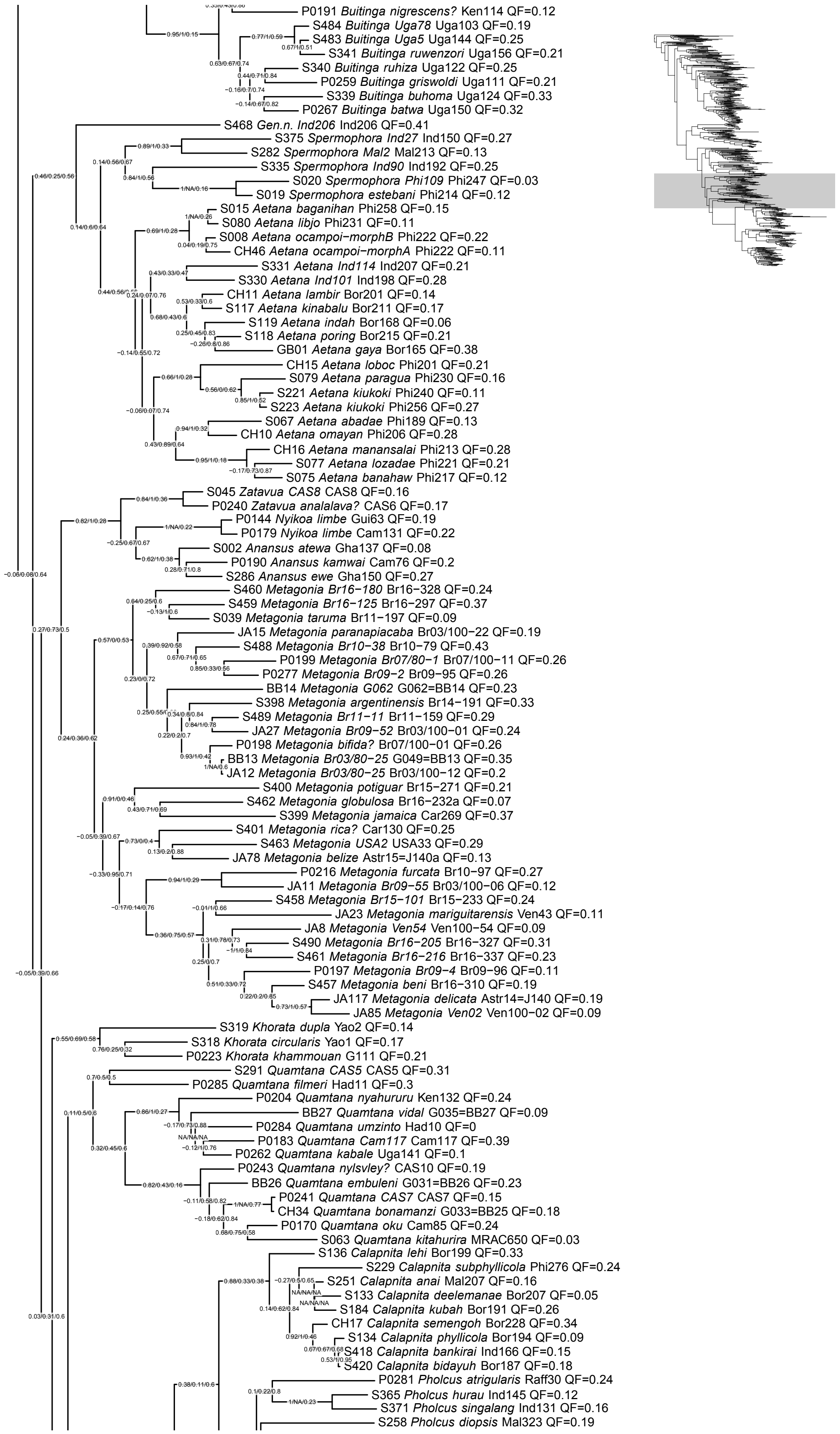

Figure S4 (e). Caption on page 16.

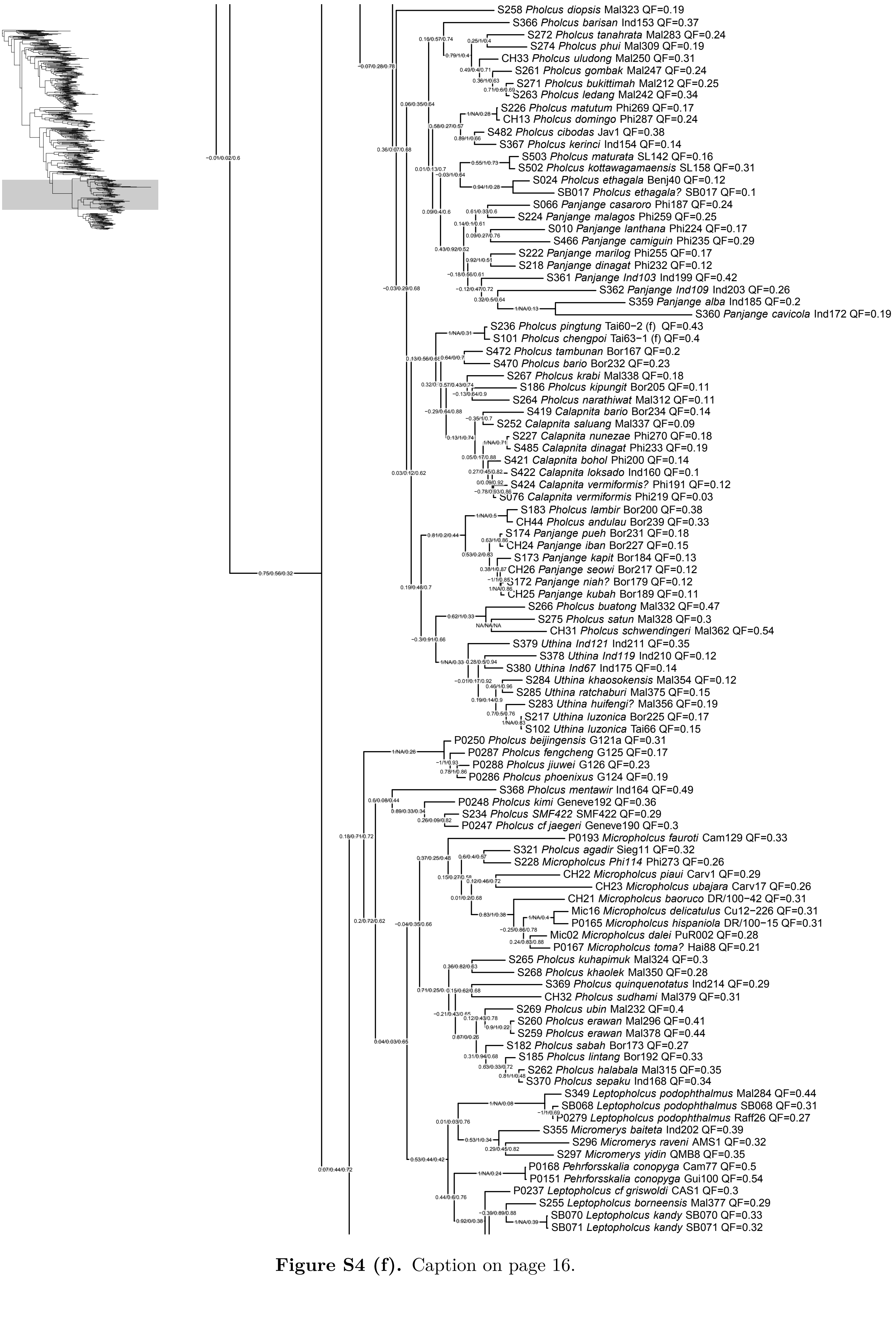

Figure S4 (f). Caption on page 16.

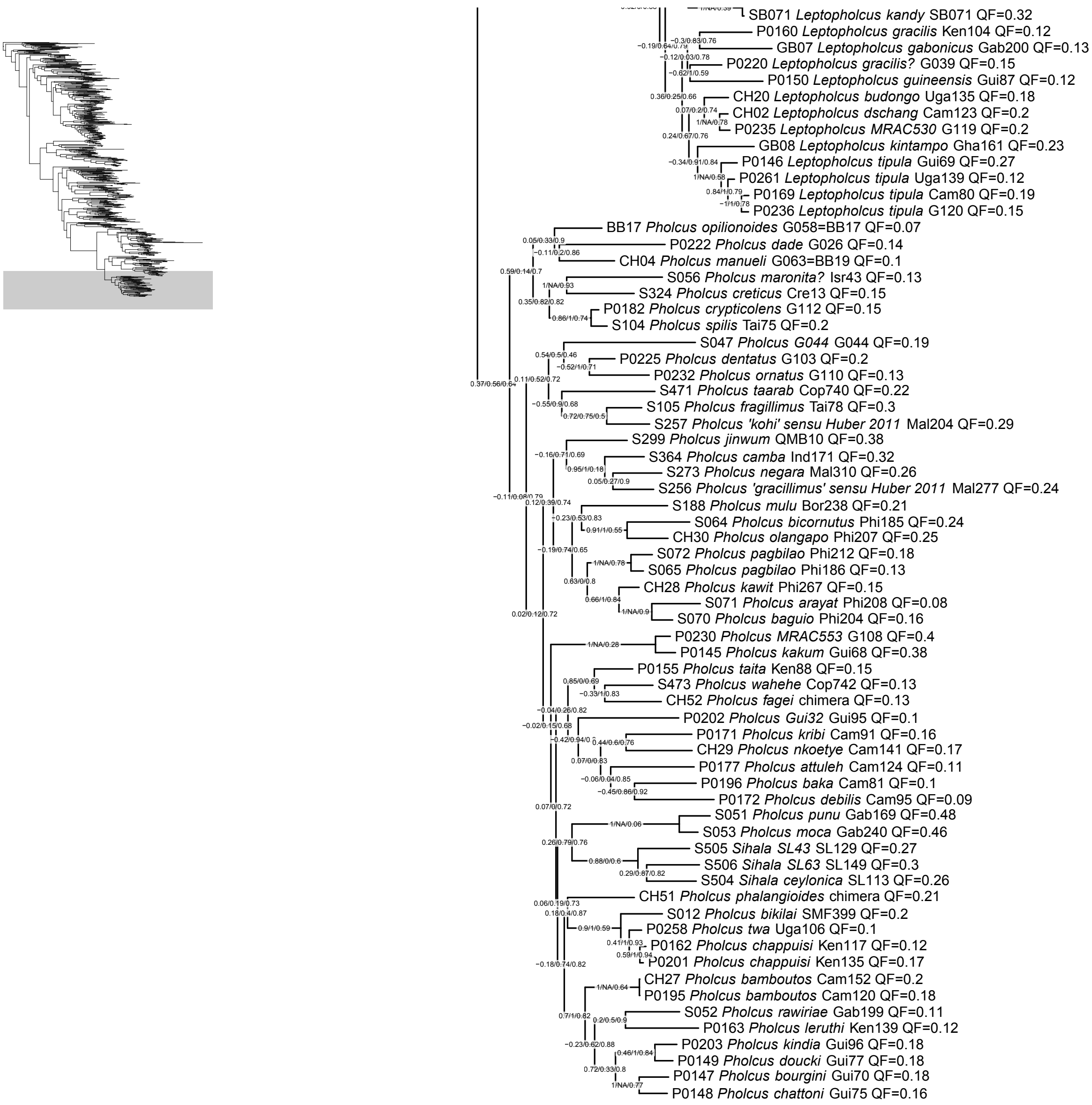

Figure S4 (g). Best maximum likelihood tree found by RAxML for the complete sampling. Branch support values are quartetsampling scores (QC/QD/QU). QF scores are given at tips. Tip labels are composed of the specimens' code, species name, and collection vial number.

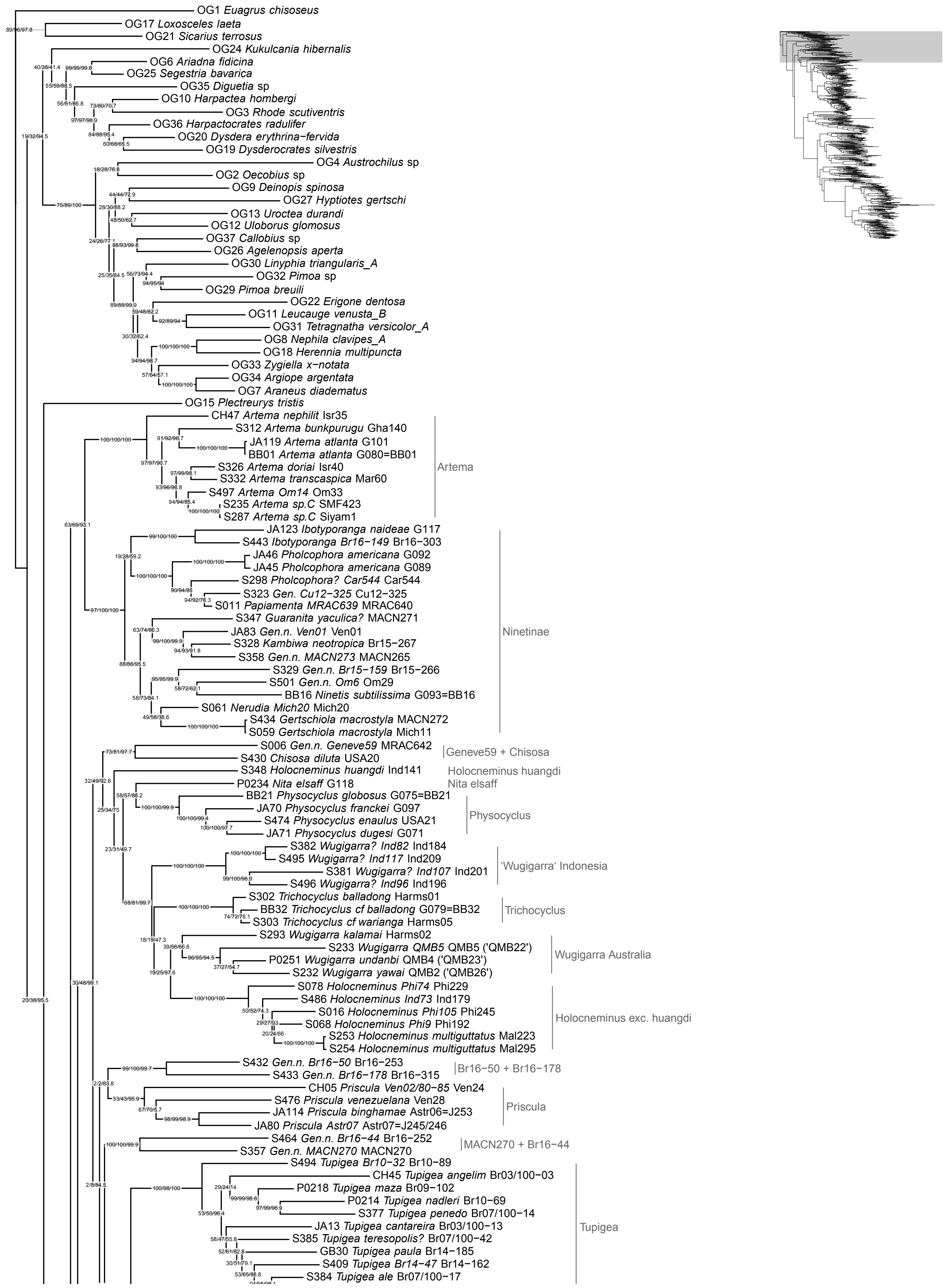

Figure S5 (a). Caption on page 23.

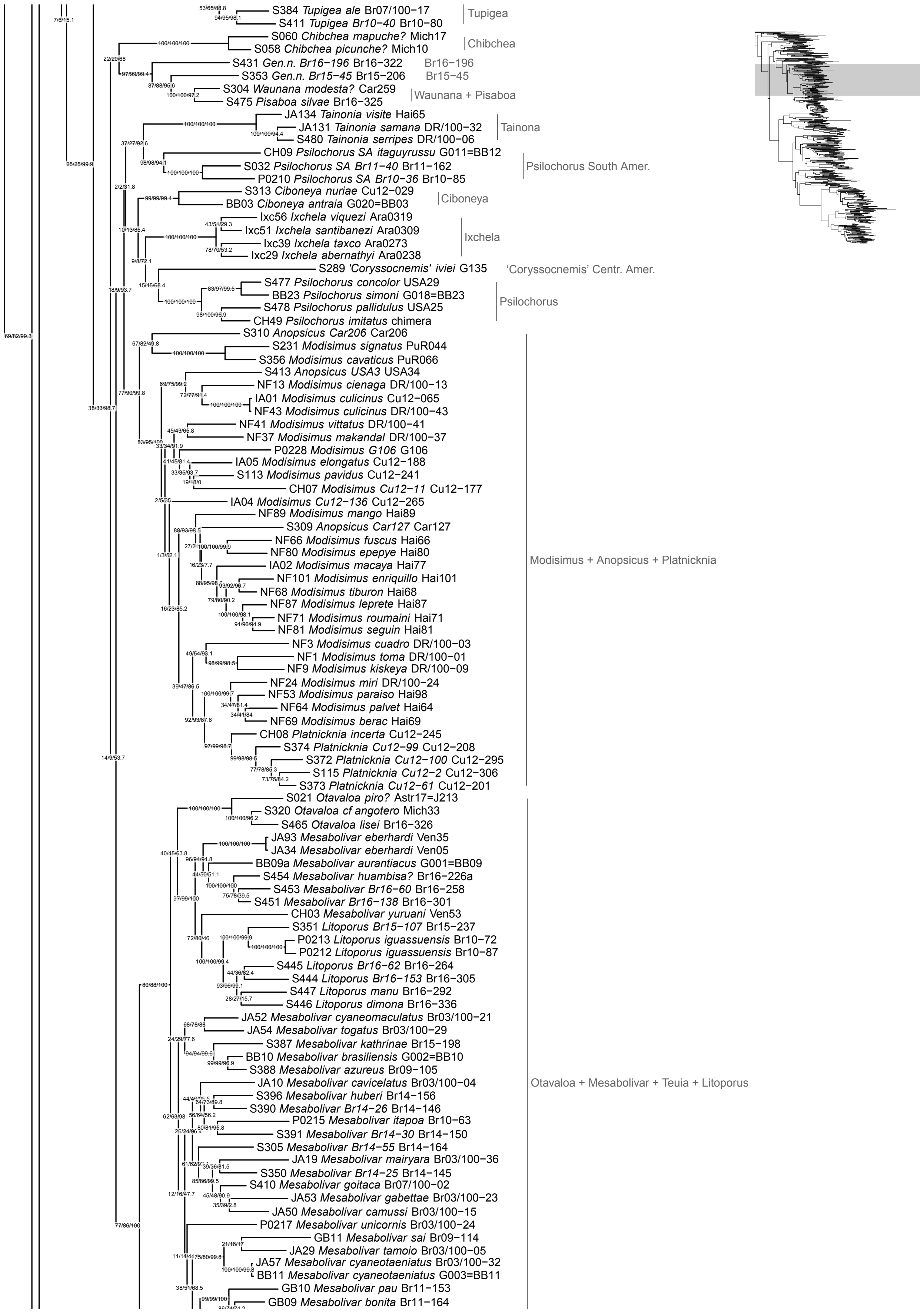

Figure S5 (b). Caption on page 23.

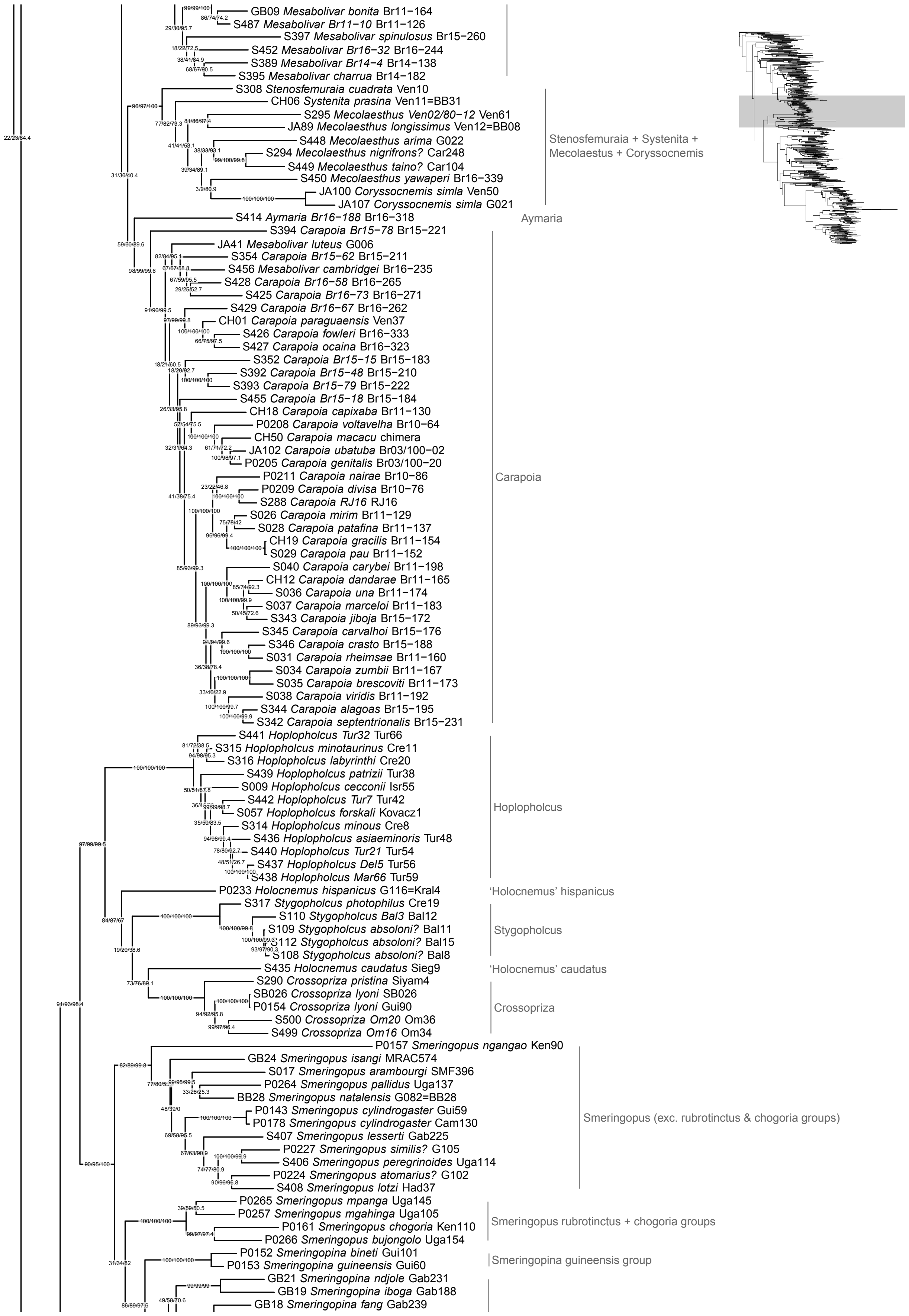

Figure S5 (c). Caption on page 23.

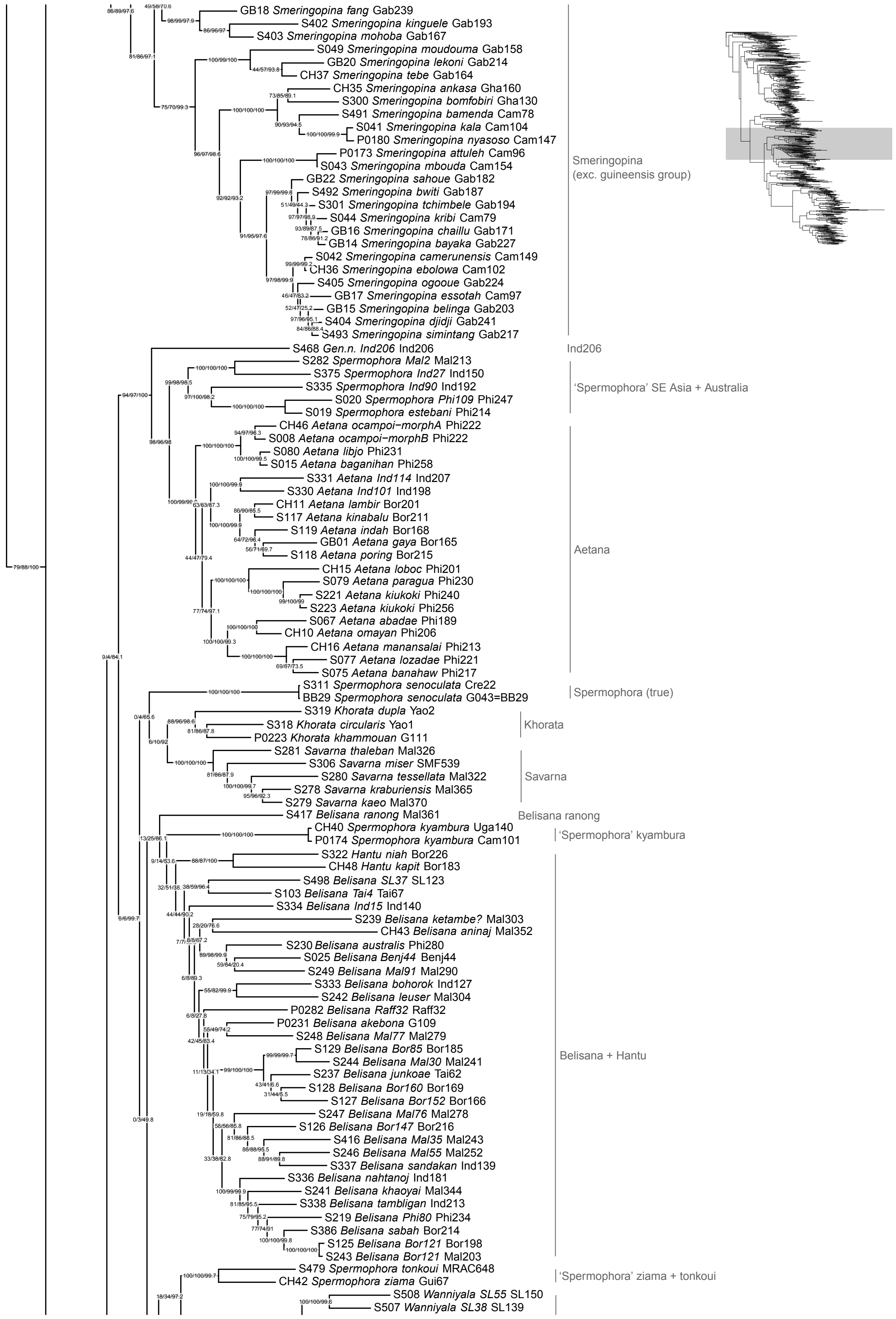

Figure S5 (d). Caption on page 23.

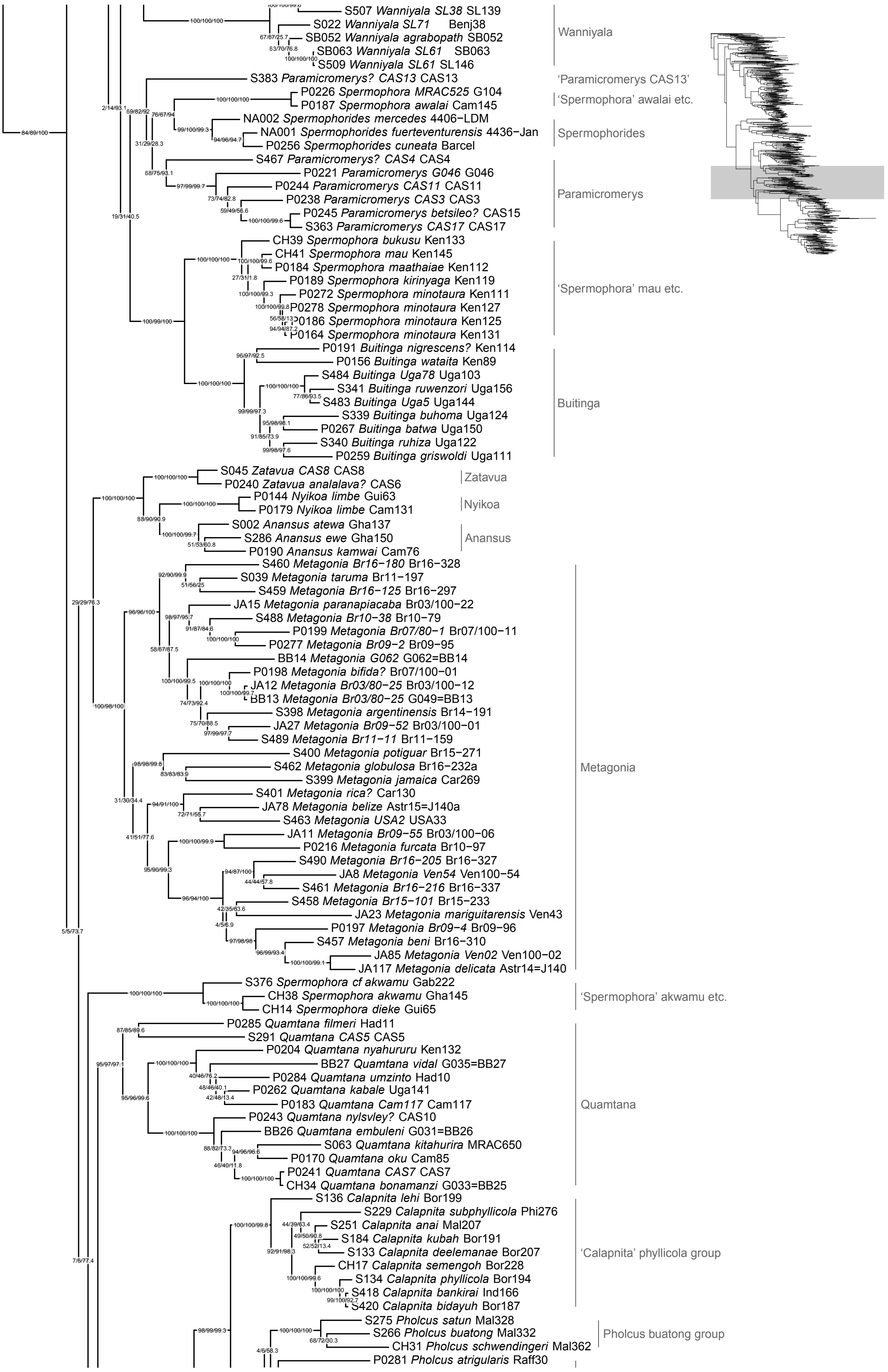

Figure S5 (e). Caption on page 23.

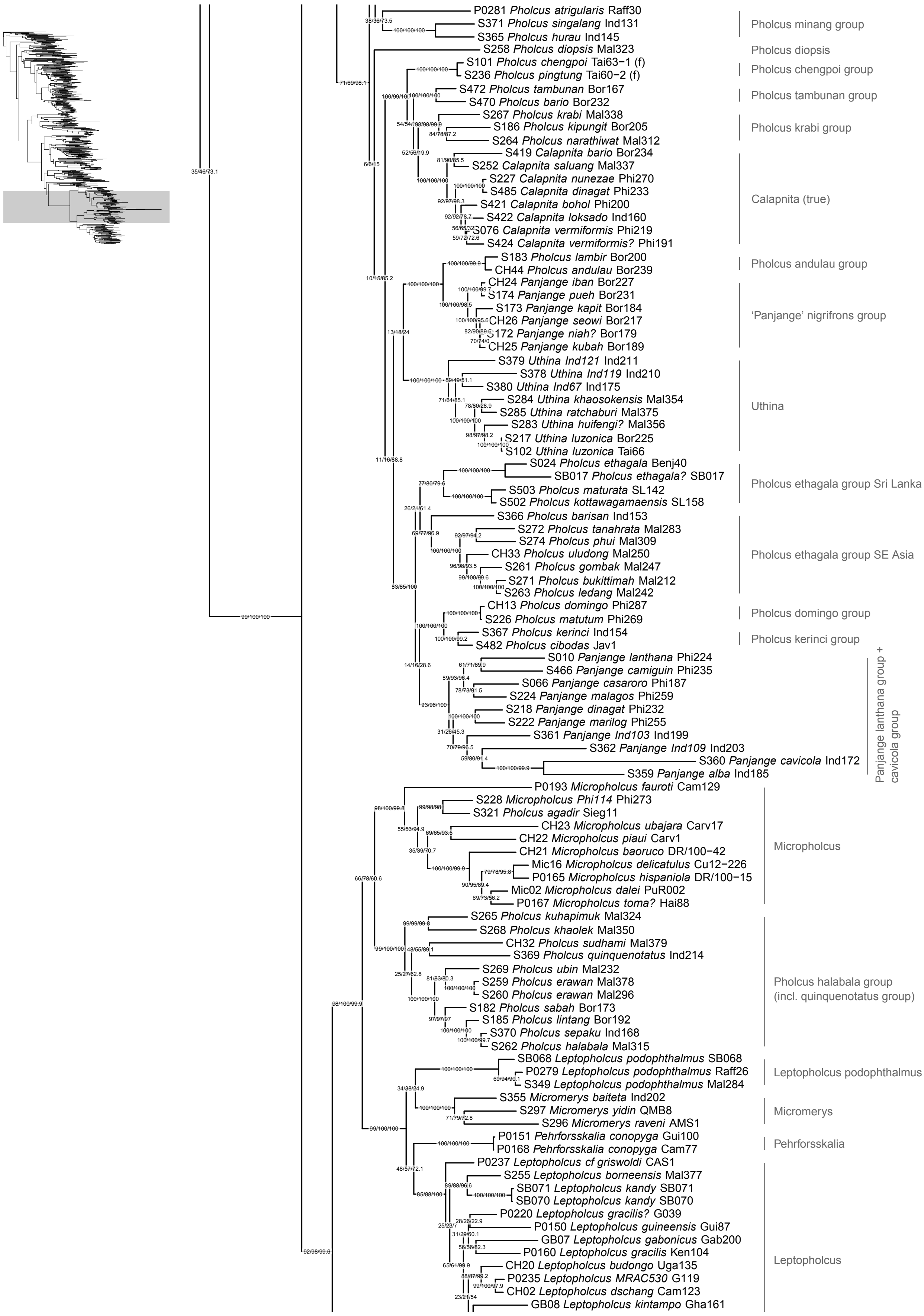

Figure S5 (f). Caption on page 23.

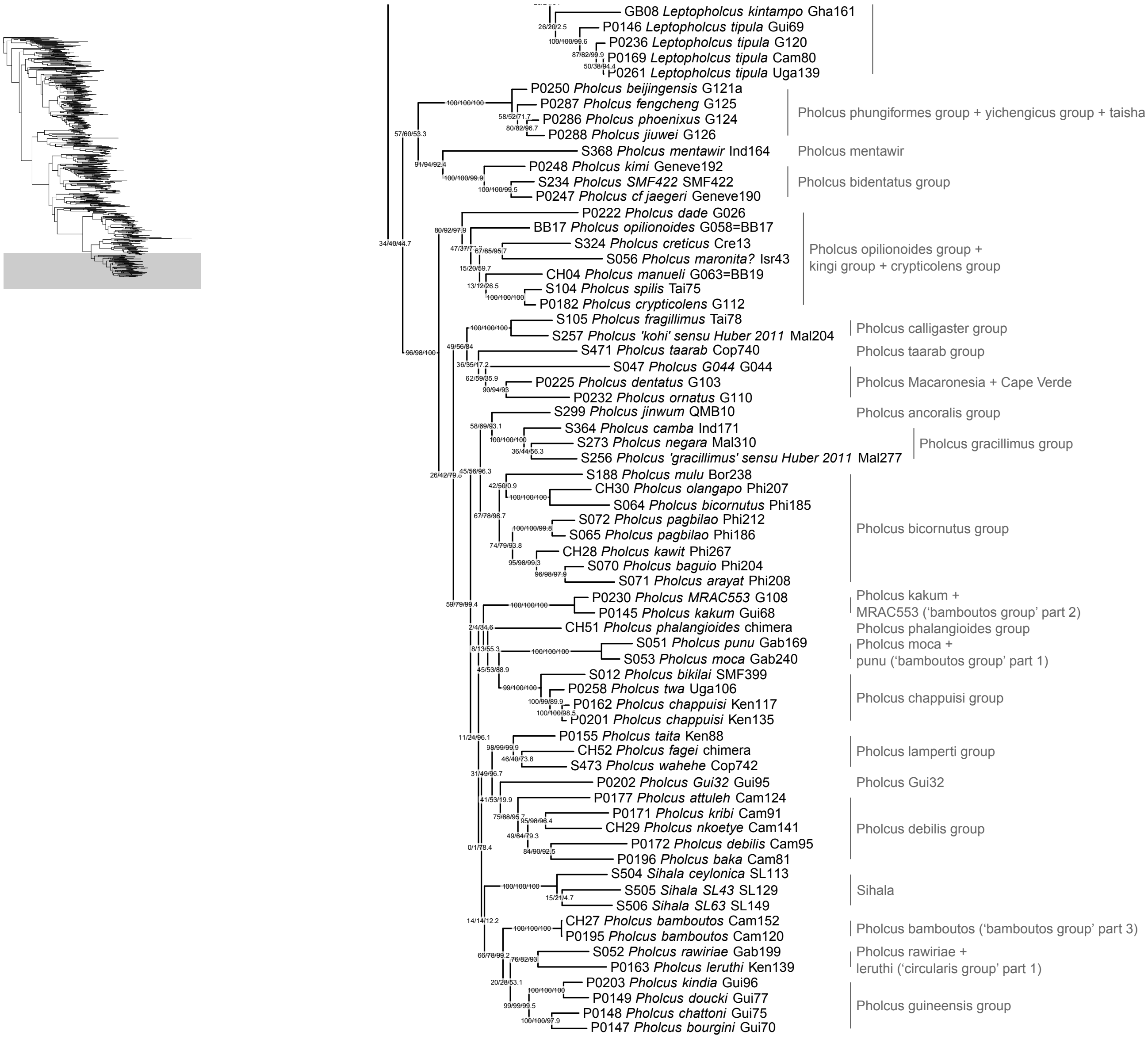

Figure S5 (g). Best maximum likelihood tree found by IQ-TREE for the complete sampling. Branch support values are standard bootstrap, rapid bootstrap and SH-like aLRT values, respectively, separated by a slash. Tip labels are composed of the specimens' code, species name, and collection vial number.

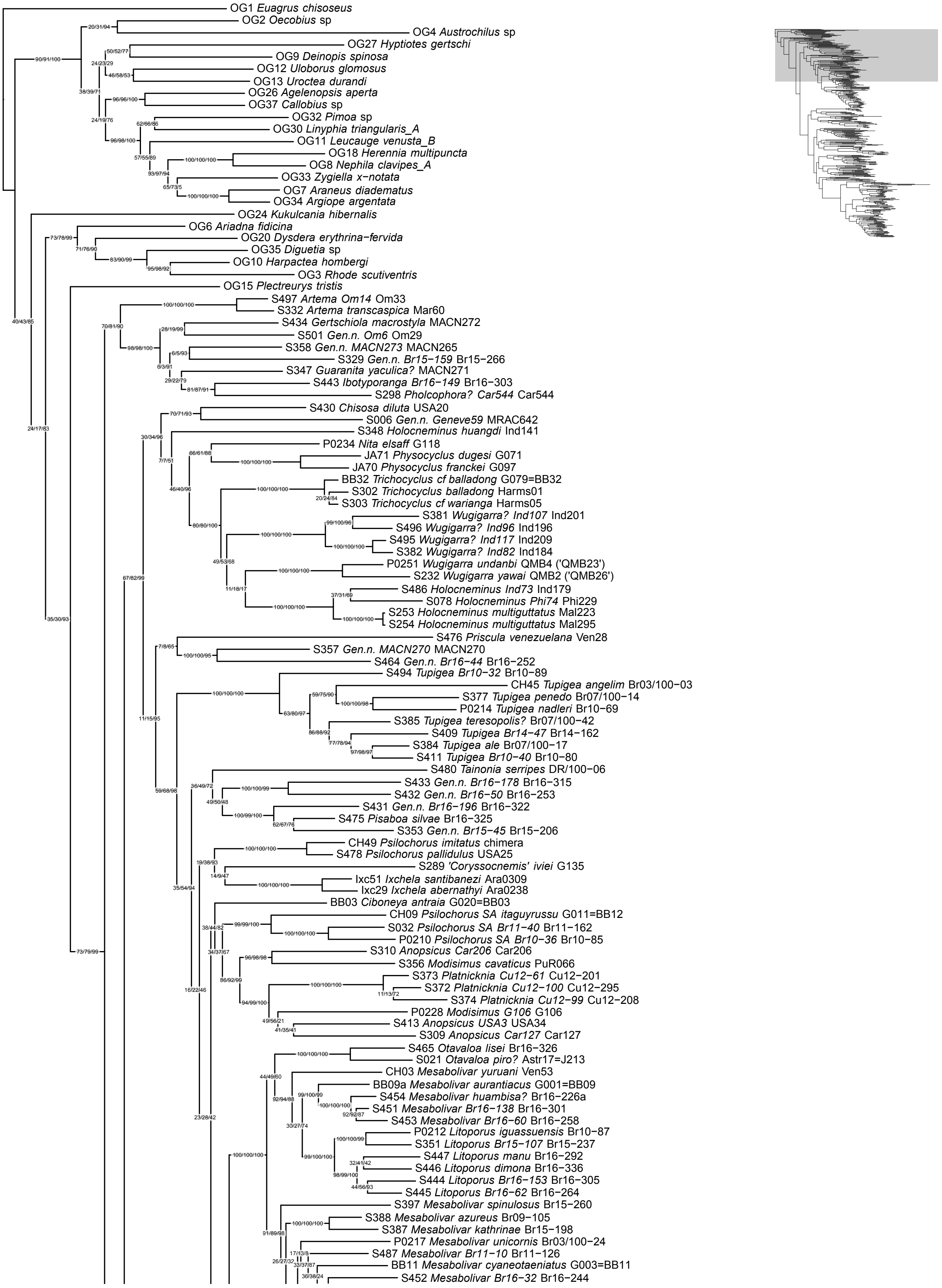

Figure S6 (a). Caption on page 27.

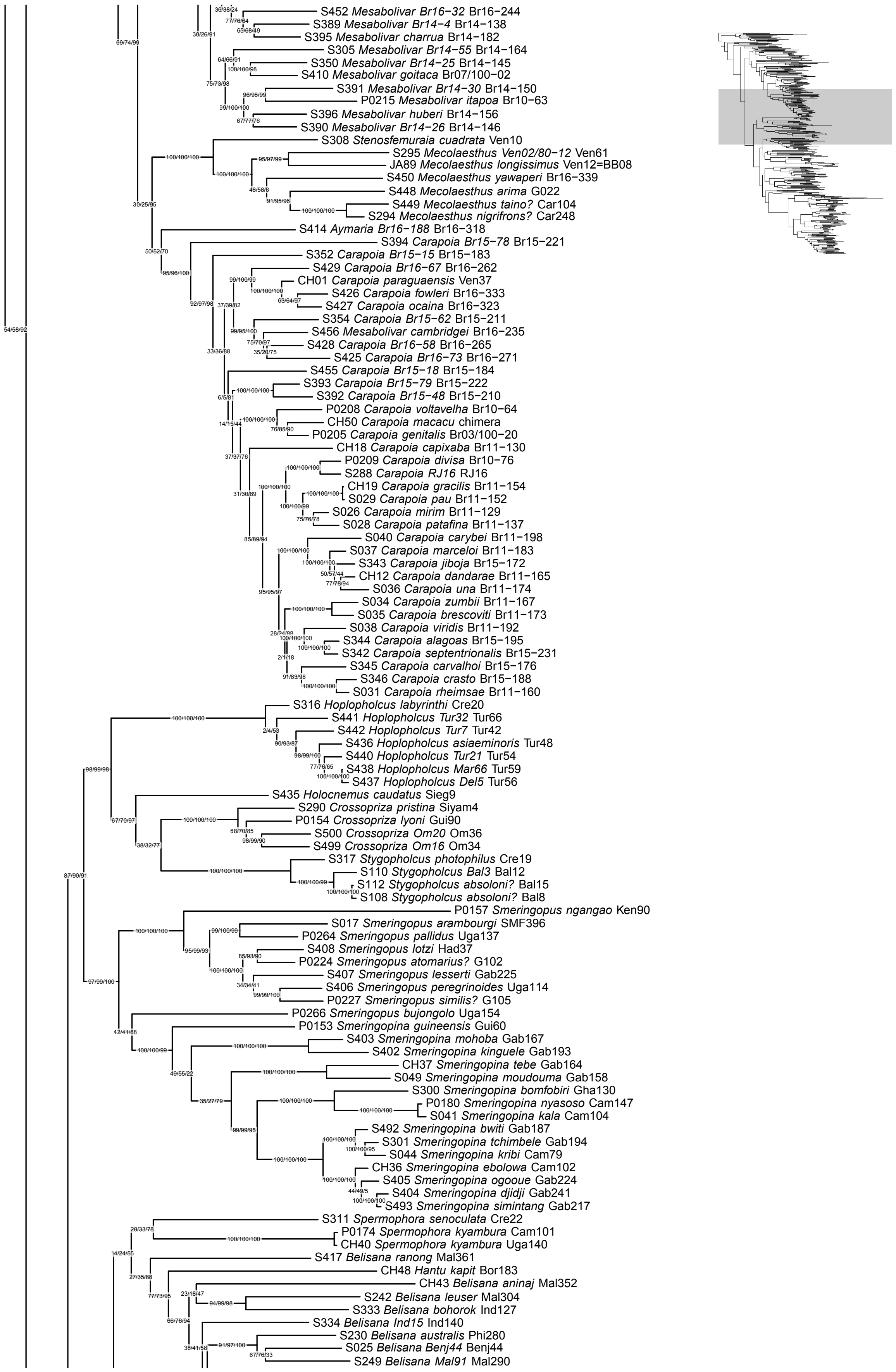

Figure S6 (b). Caption on page 27.

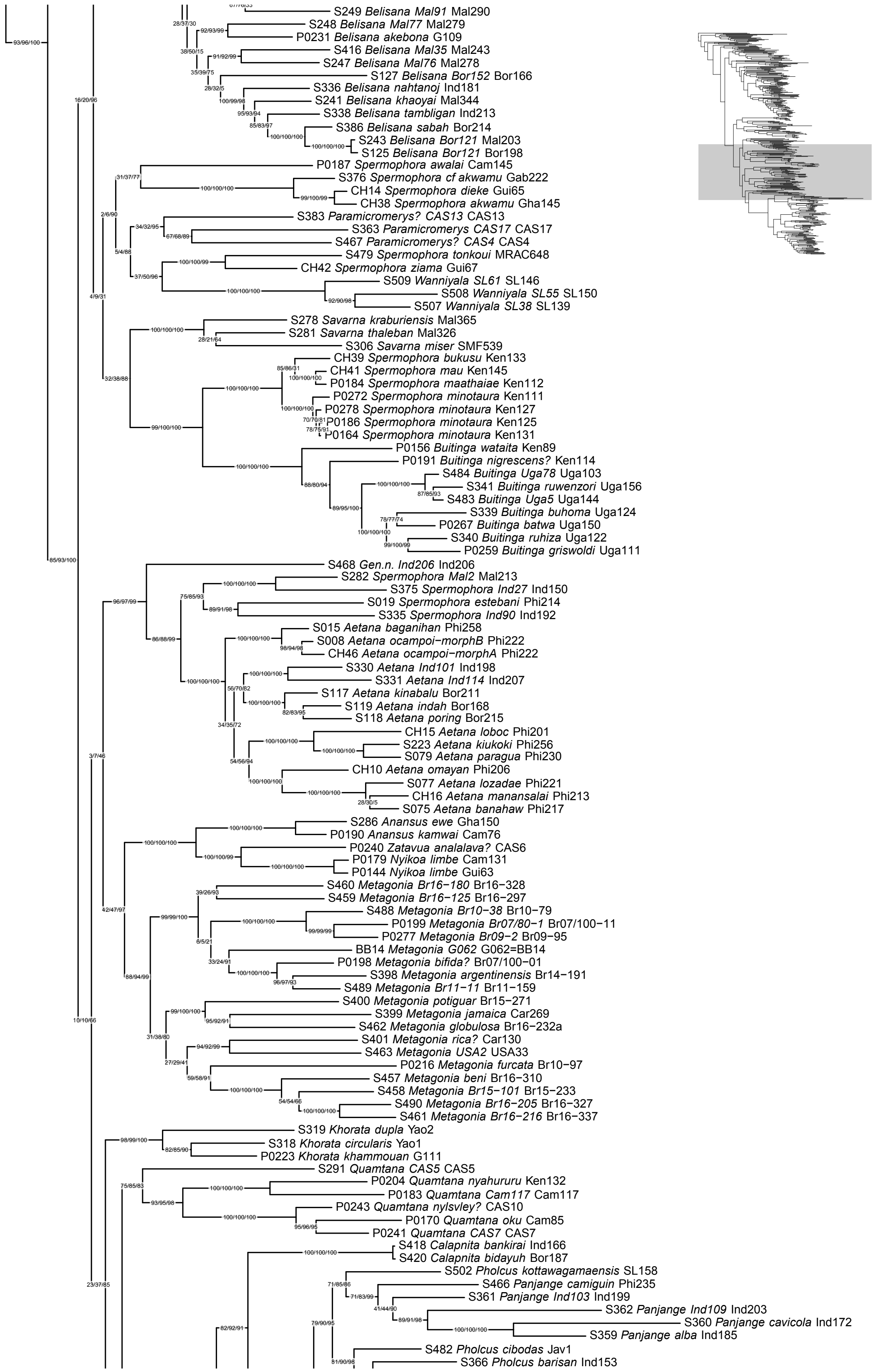

Figure S6 (c). Caption on page 27.

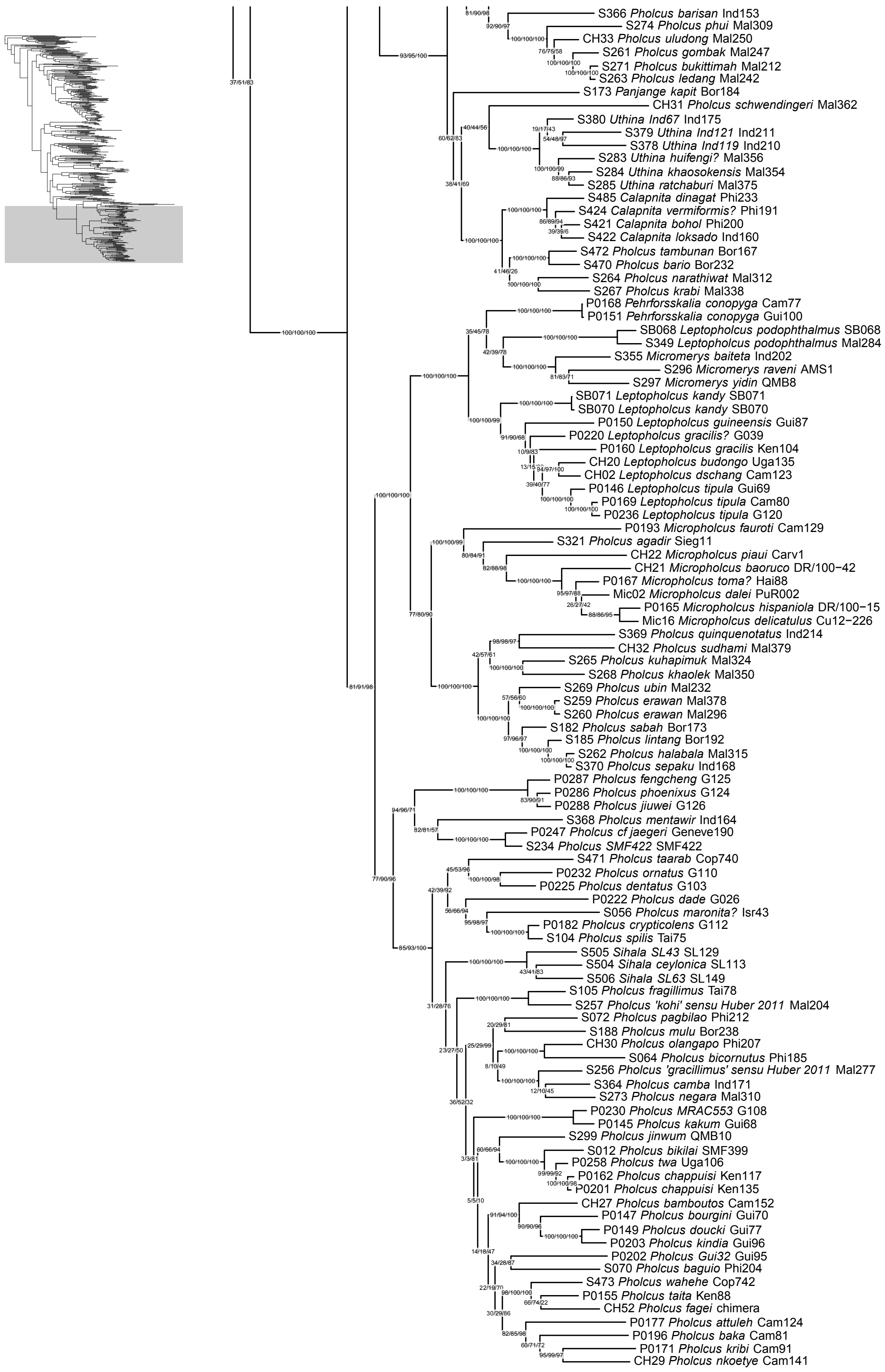

Figure S6 (d). Best maximum likelihood tree found by RAxML for the reduced sampling with minimum four loci available. Branch support values are standard bootstrap, rapid bootstrap and SH-like aLRT values, respectively, separated by a slash. Tip labels are composed of the specimens' code, species name, and collection vial number.

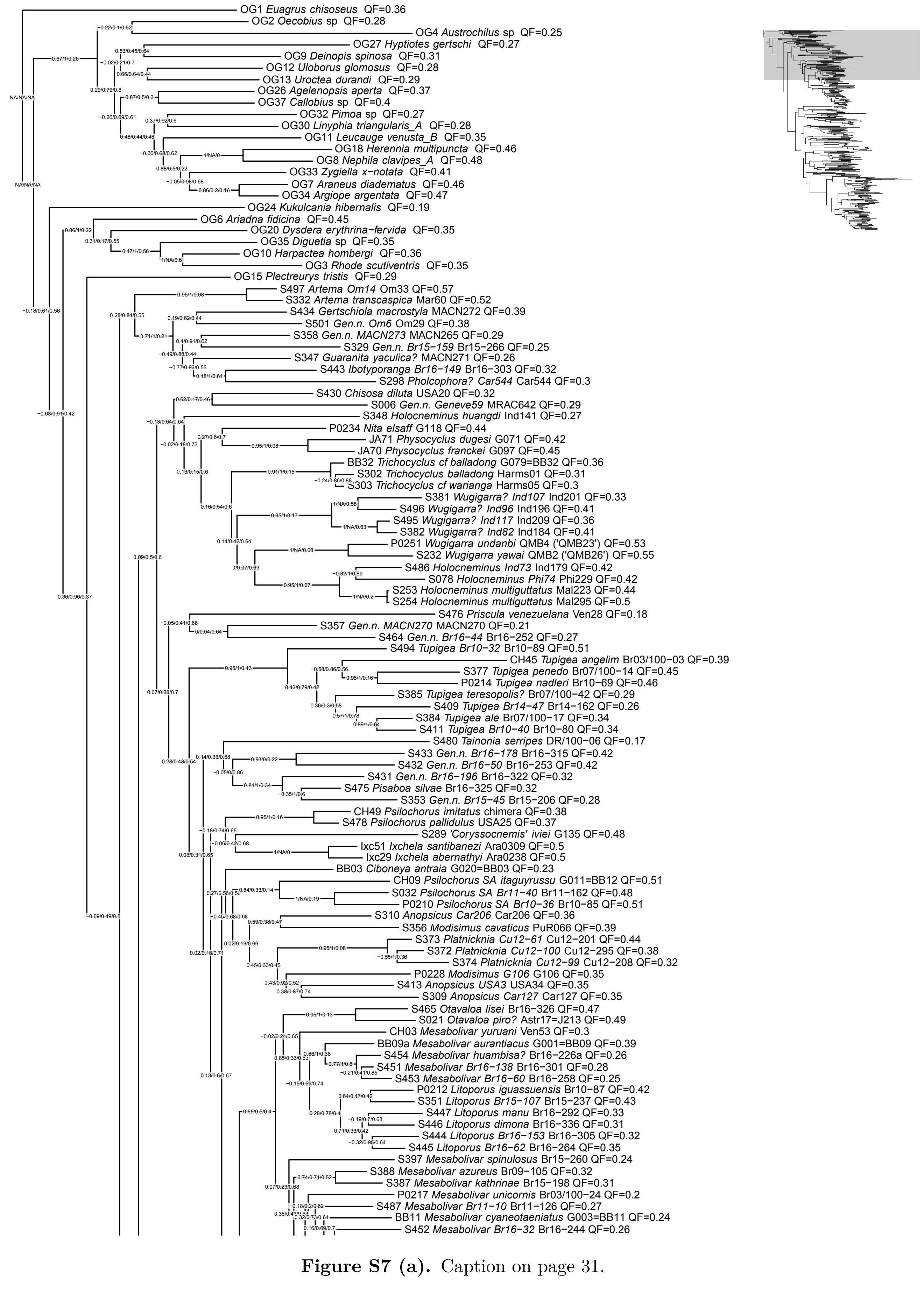

Figure S7 (a). Caption on page 31.

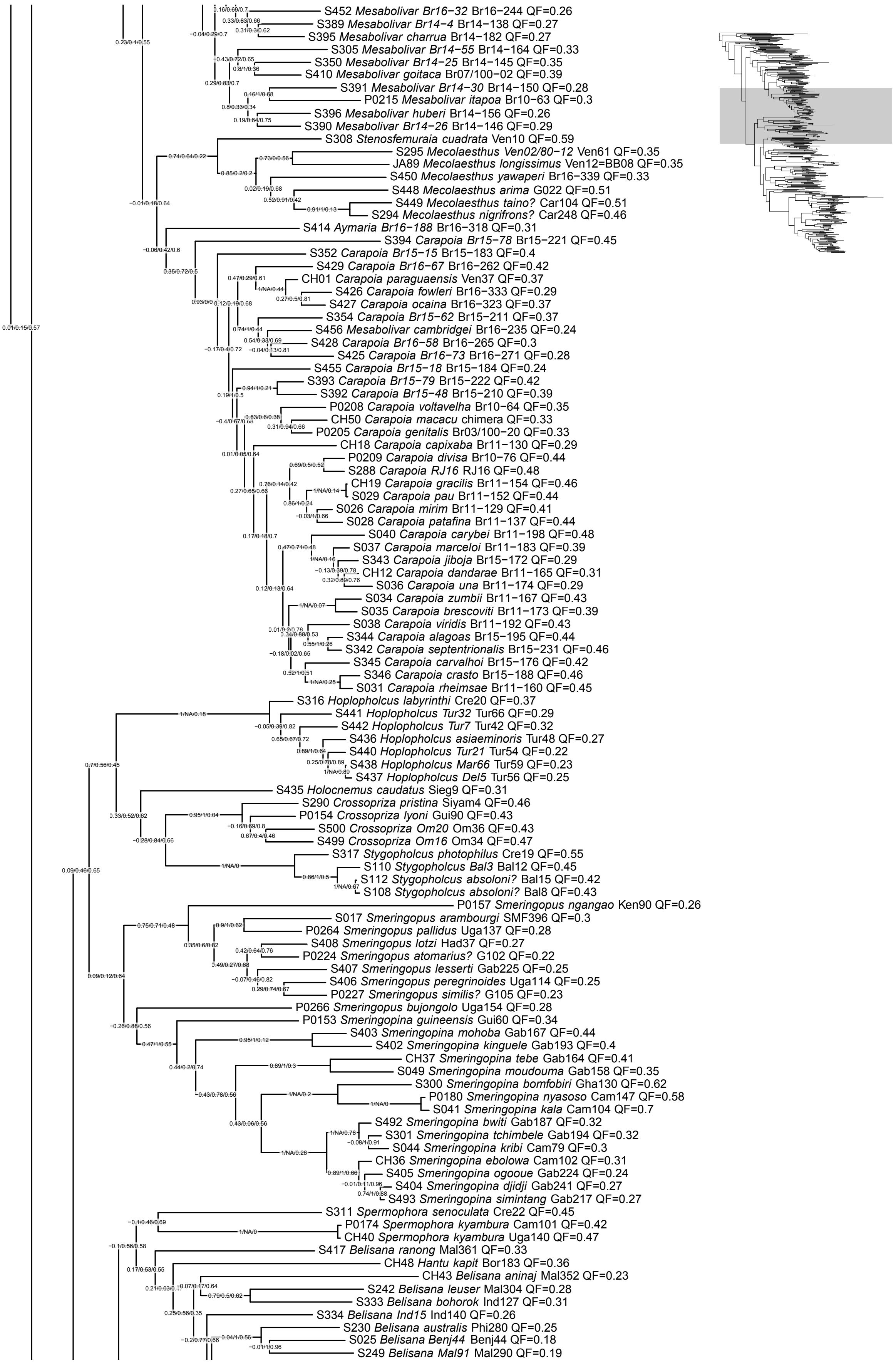

Figure S7 (b). Caption on page 31.

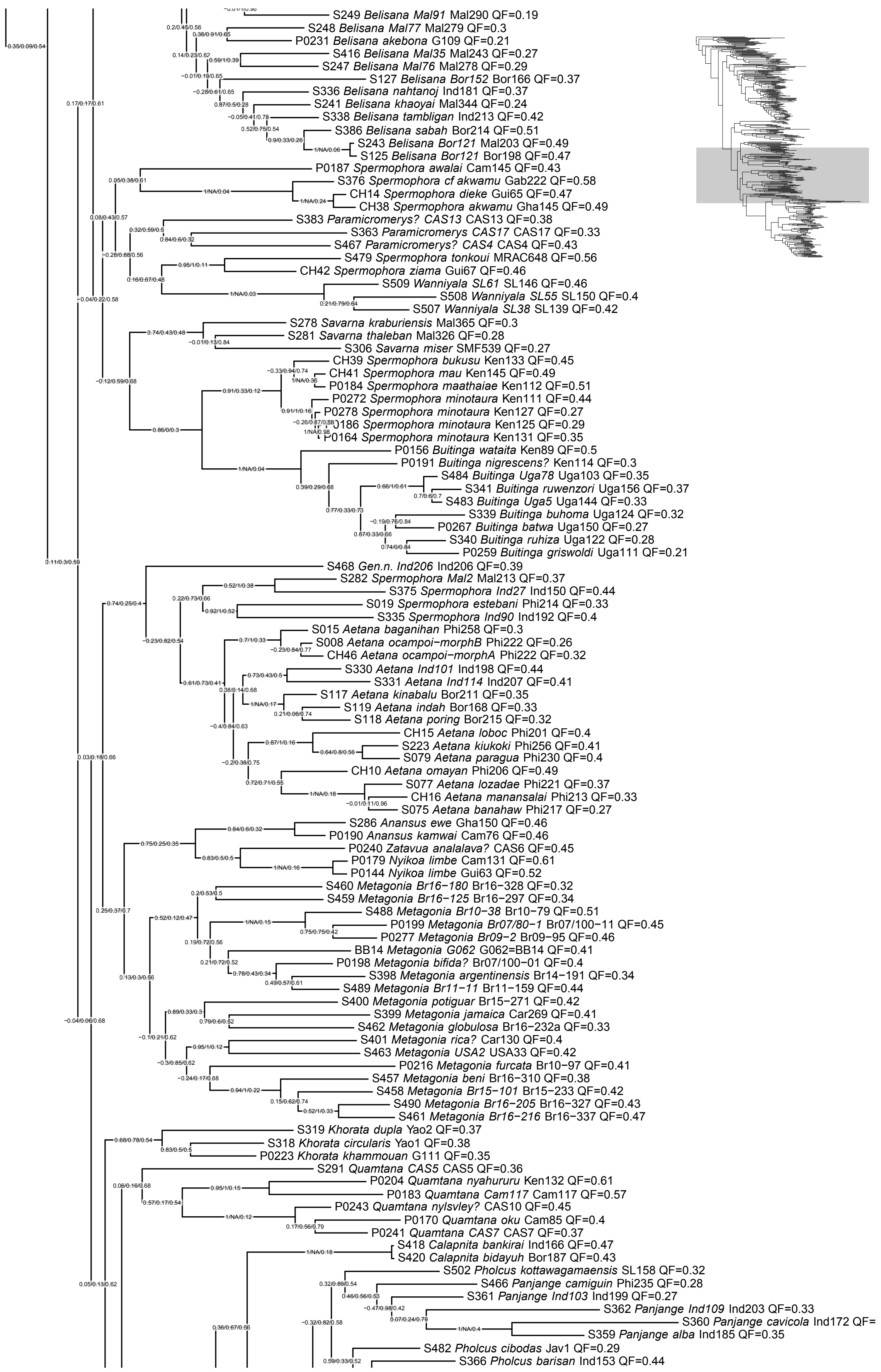

Figure S7 (c). Caption on page 31.

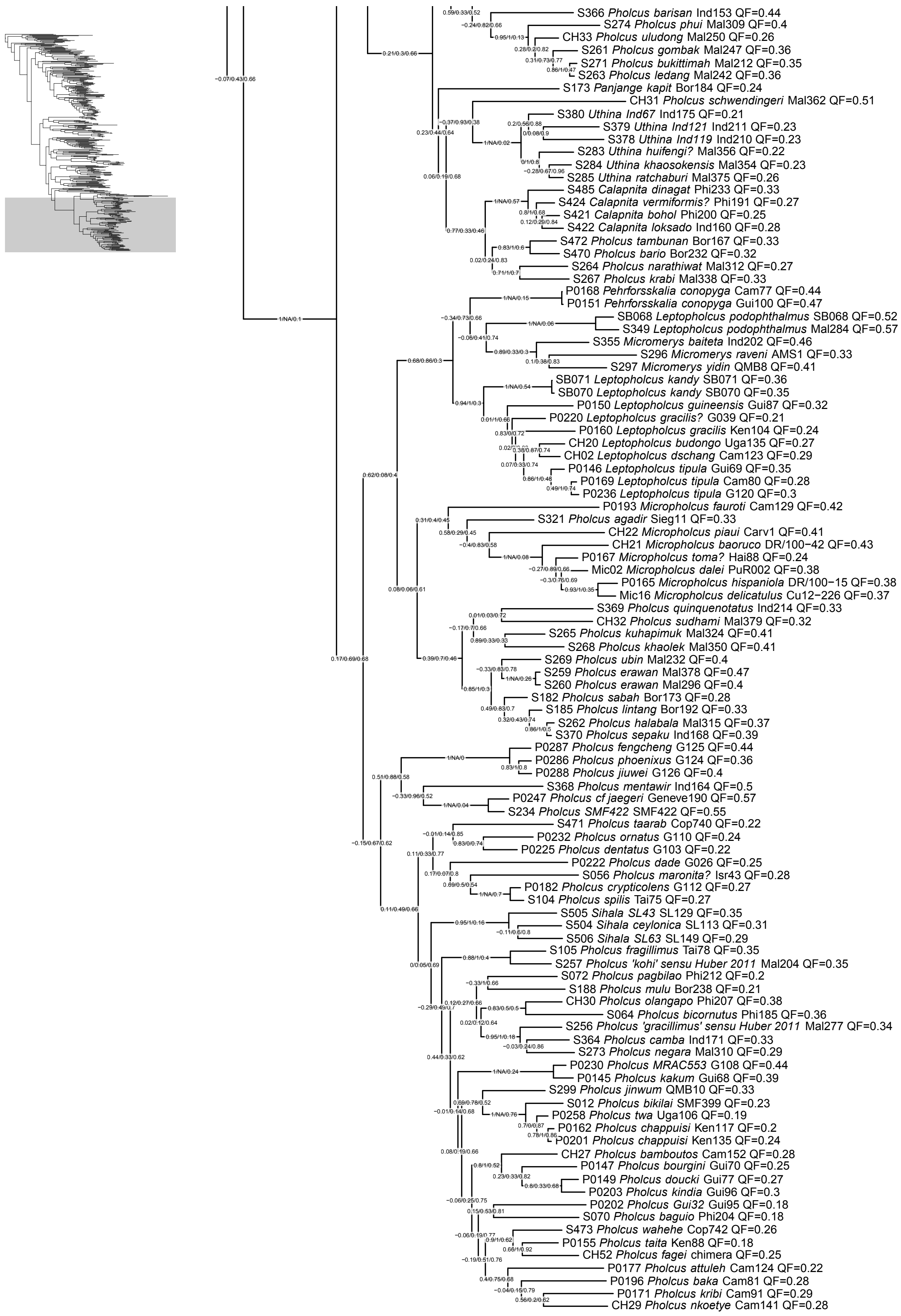

Figure S7 (d). Best maximum likelihood tree found by RAxML for the reduced sampling with minimum four loci available. Branch support values are quartetsampling scores (QC/QD/QU). QF scores are given at tips. Tip labels are composed of the specimens' code, species name, and collection vial number.

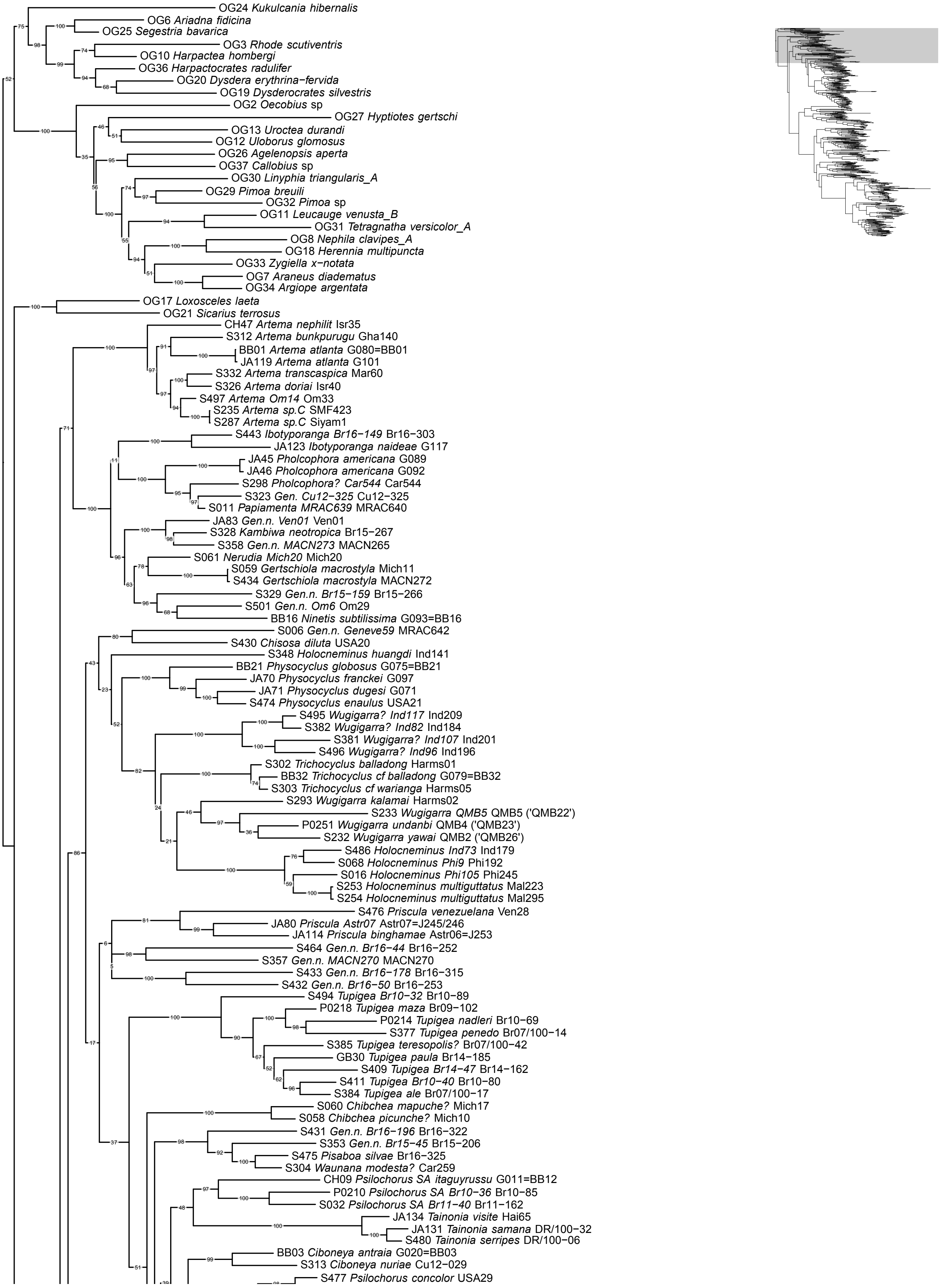

Figure S8 (a). Caption on page 37.

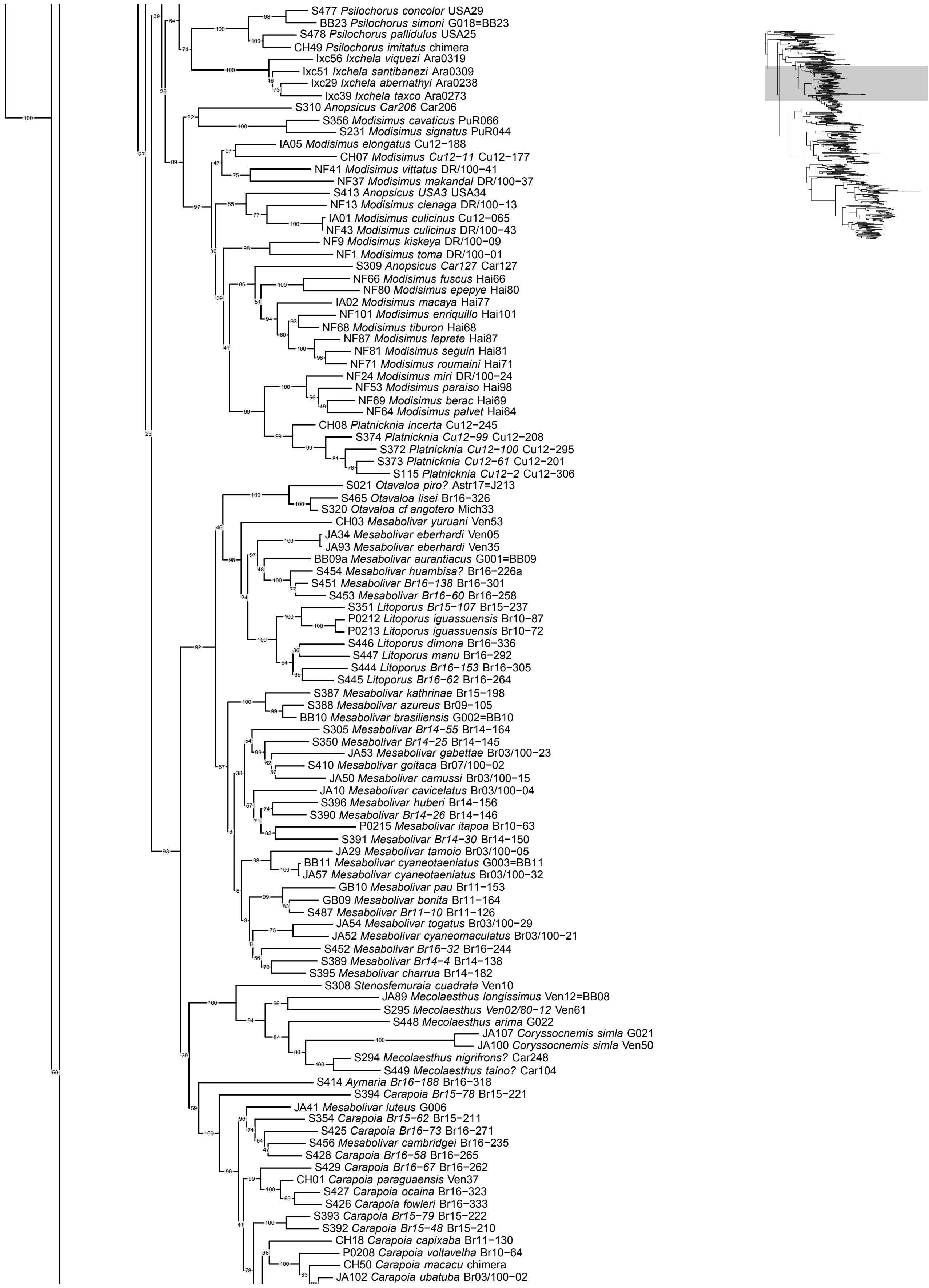

Figure S8 (b). Caption on page 37.

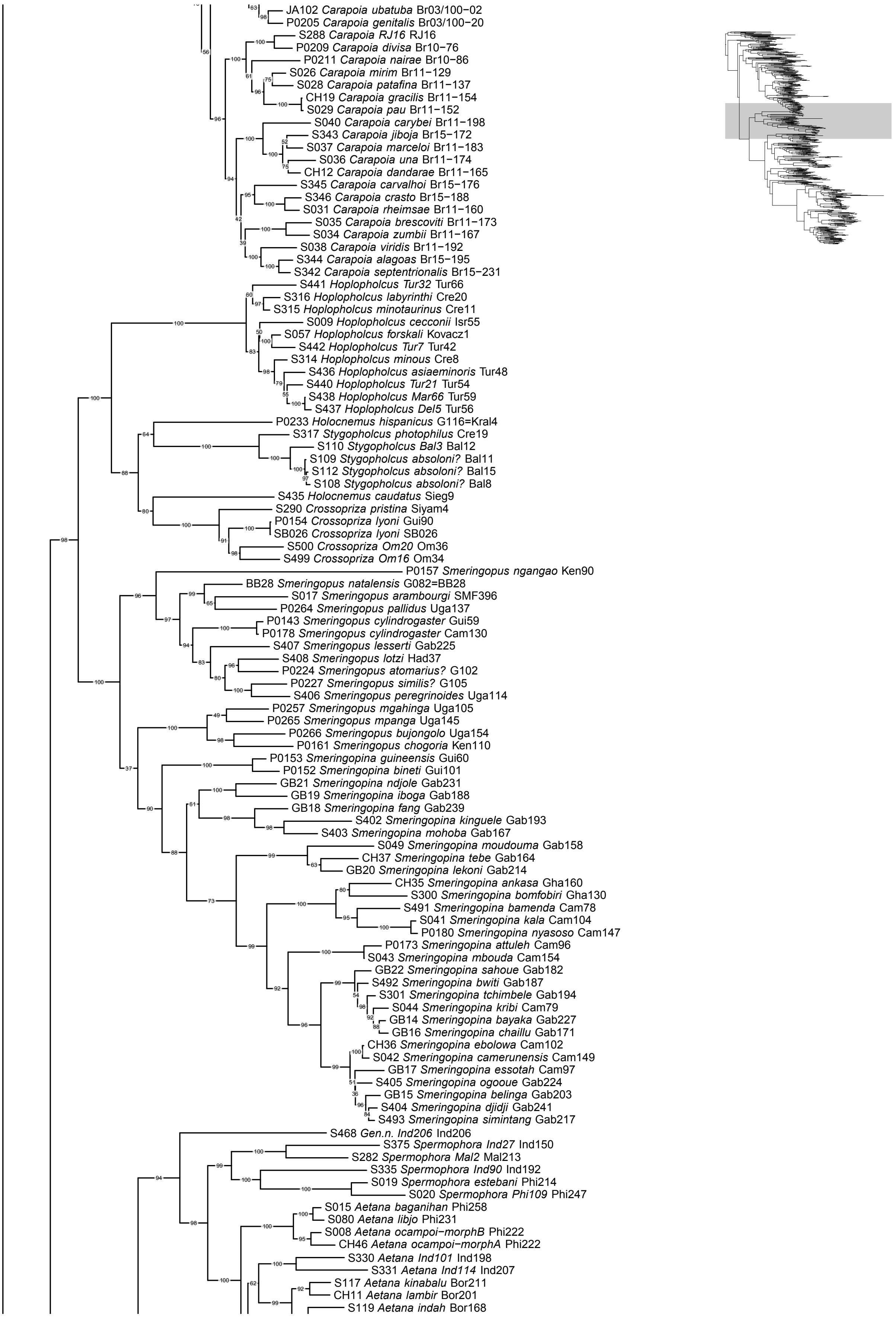

Figure S8 (c). Caption on page 37.

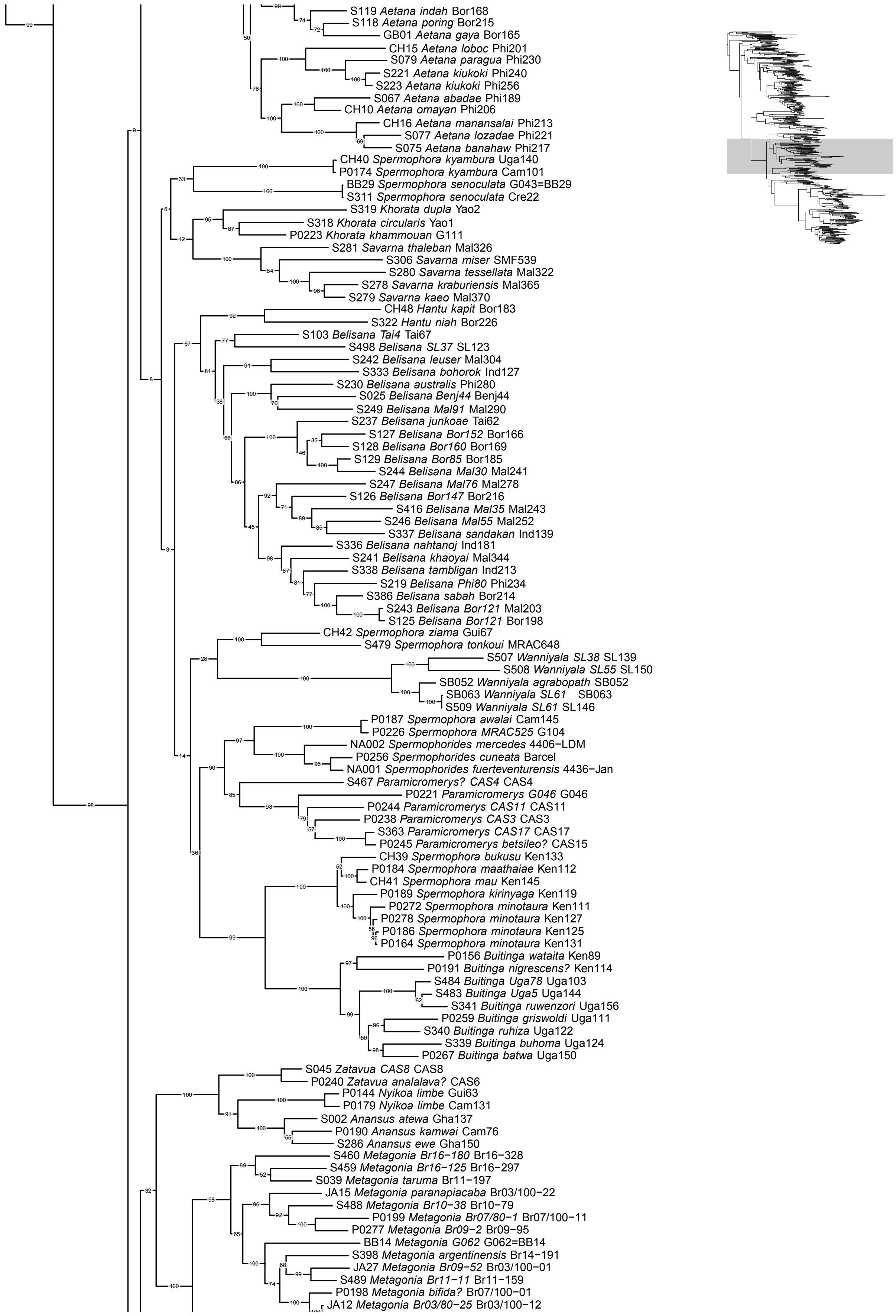

Figure S8 (d). Caption on page 37.

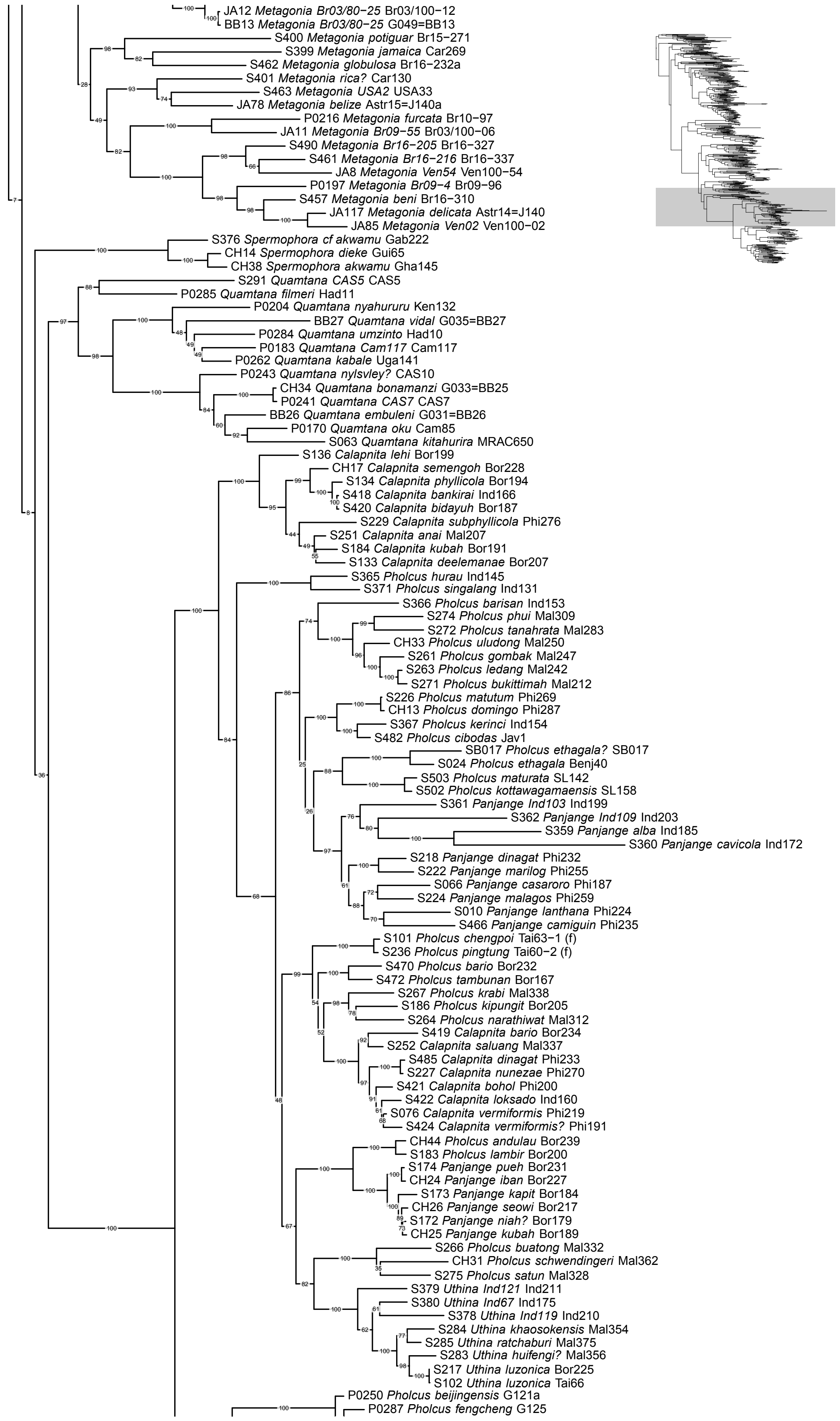

Figure S8 (e). Caption on page 37.

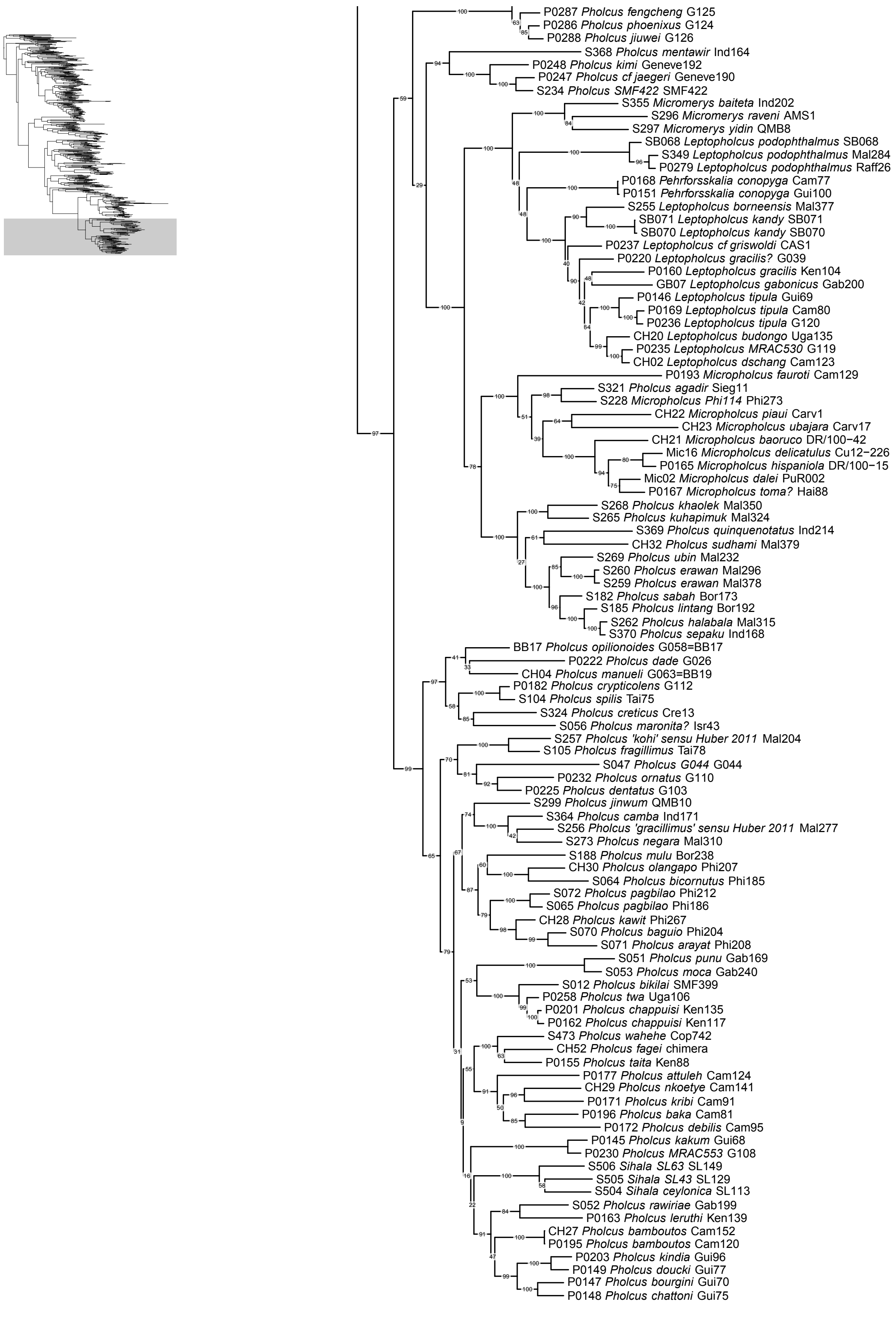

Figure S8 (f). Best maximum likelihood tree found by RAxML for the data set reduced by RogueNaRok. Branch support values rapid bootstrap scores. Tip labels are composed of the specimens' code, species name, and collection vial number.

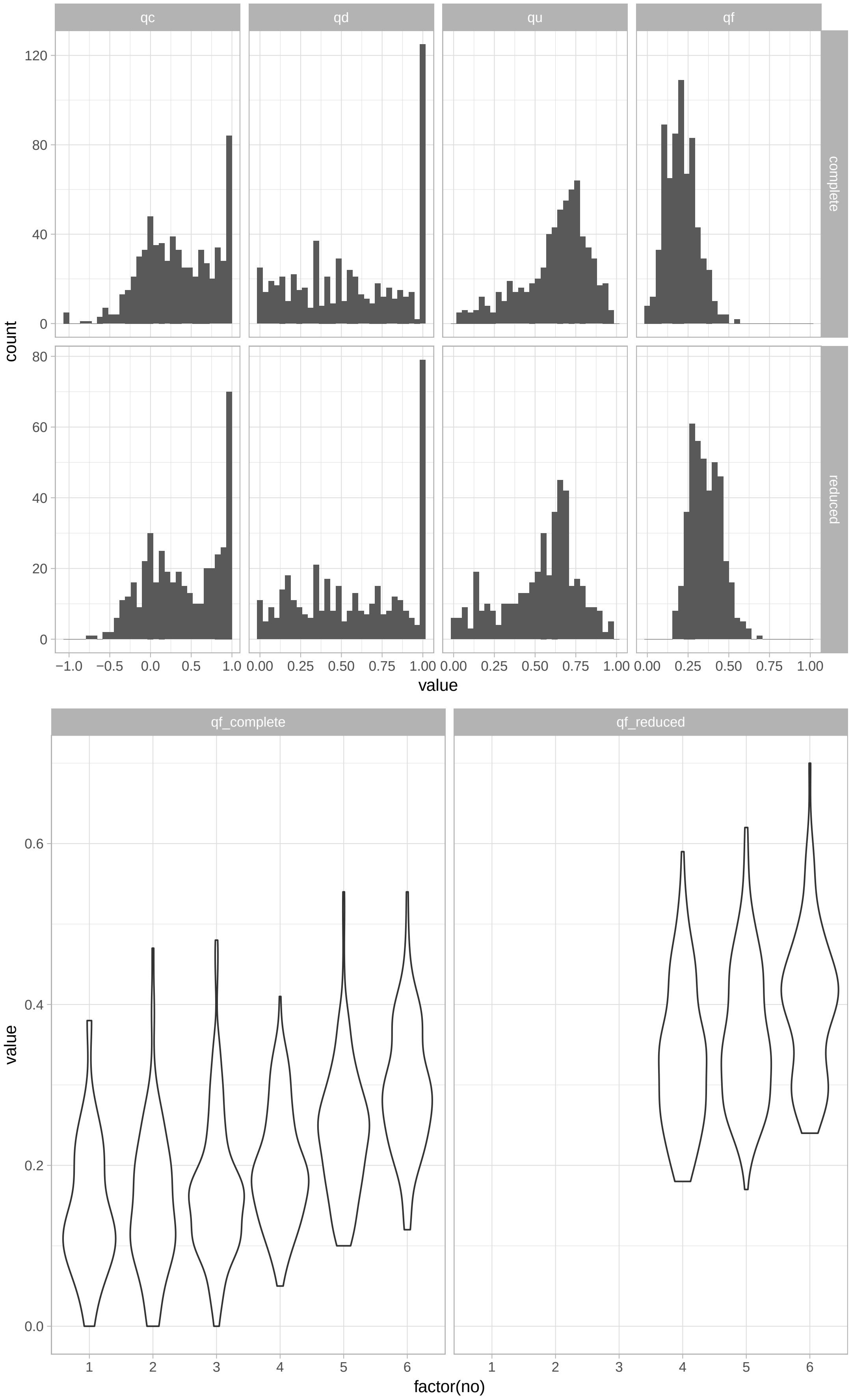

Figure S9: Branch support comparison between RAxML trees for the complete sampling (Fig. S4) and reduced sampling with minimum 4 loci available (Fig. S7). Upper half: Histograms of quartet sampling scores; lower half: equal-area violin plots of QF scores (low values indicate taxa with instable phylogenetic position).

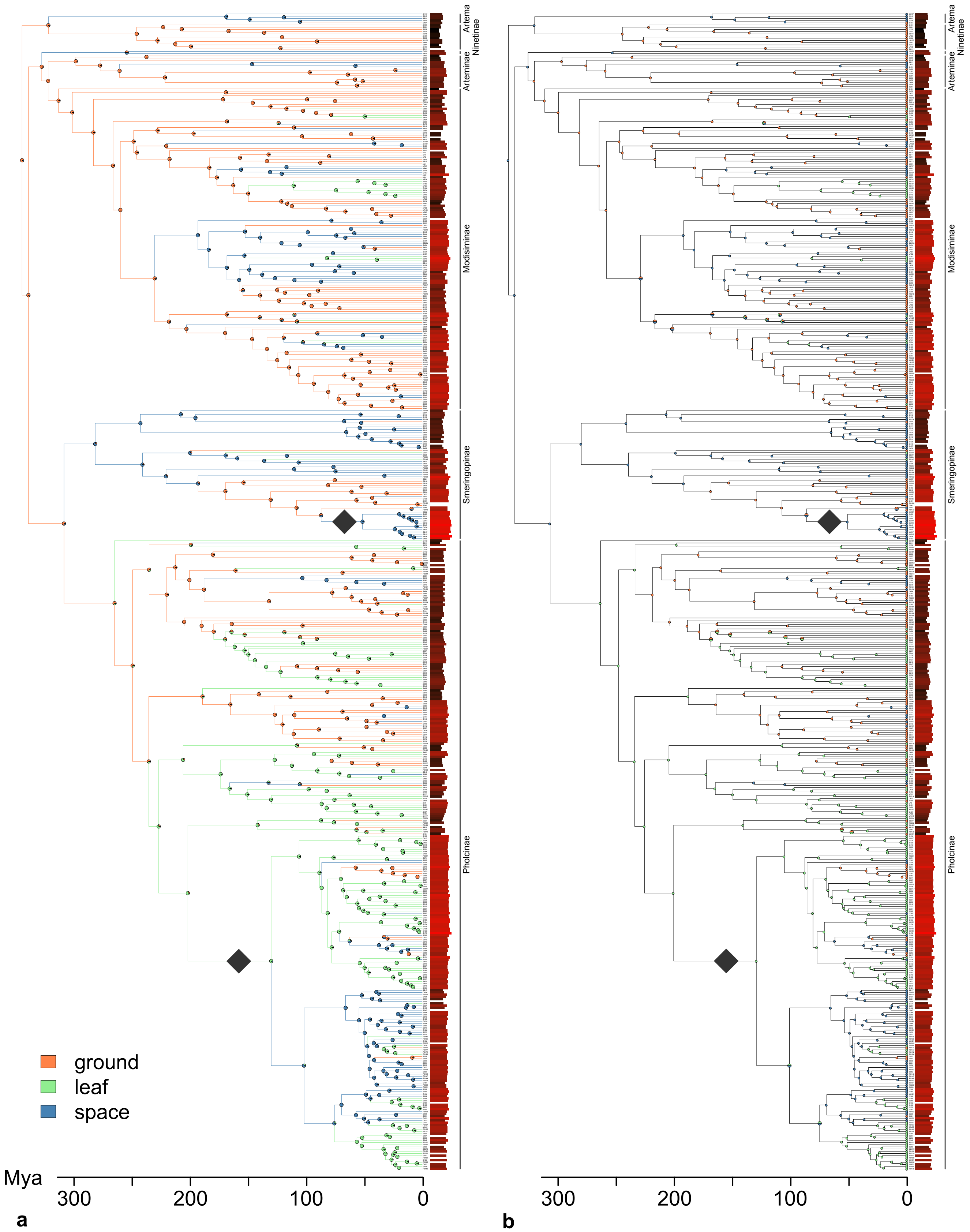

Figure S10: Time tree inferred with treePL with ancestral states (a) inferred by maximum likelihood and (b) reconstructed by maximum parsimony. Pies at nodes in (a) depict ancestral state likelihoods while branch colors in code the most likely state. Pies at nodes in (b) give the most parsimonious state(s). Bars next to tips illustrate the ratio of metatarsus to tibia of the first leg which was used as a proxy of microhabitat. Diamonds show speciation rate shifts of the best fitting scenario inferred with BAMM.

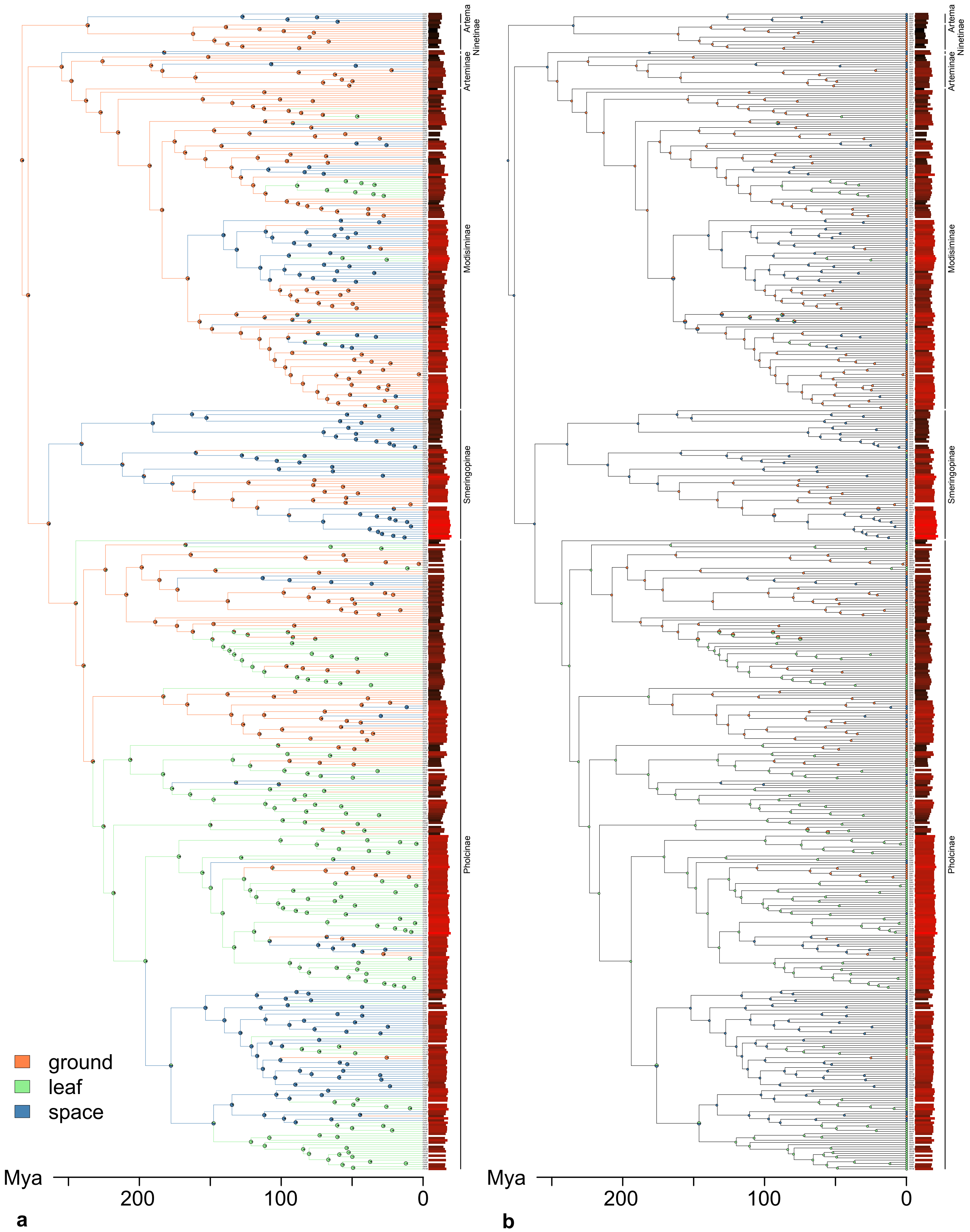

Figure S11: Time tree inferred with MCMCtree with ancestral states (a) inferred by maximum likelihood and (b) reconstructed by maximum parsimony. Pies at nodes in (a) depict ancestral state likelihoods while branch colors in code the most likely state. Pies at nodes in (b) give the most parsimonious state(s). Bars next to tips illustrate the ratio of metatarsus to tibia of the first leg which was used as a proxy of microhabitat. Diamonds show speciation rate shifts of the best fitting scenario inferred with BAMM.

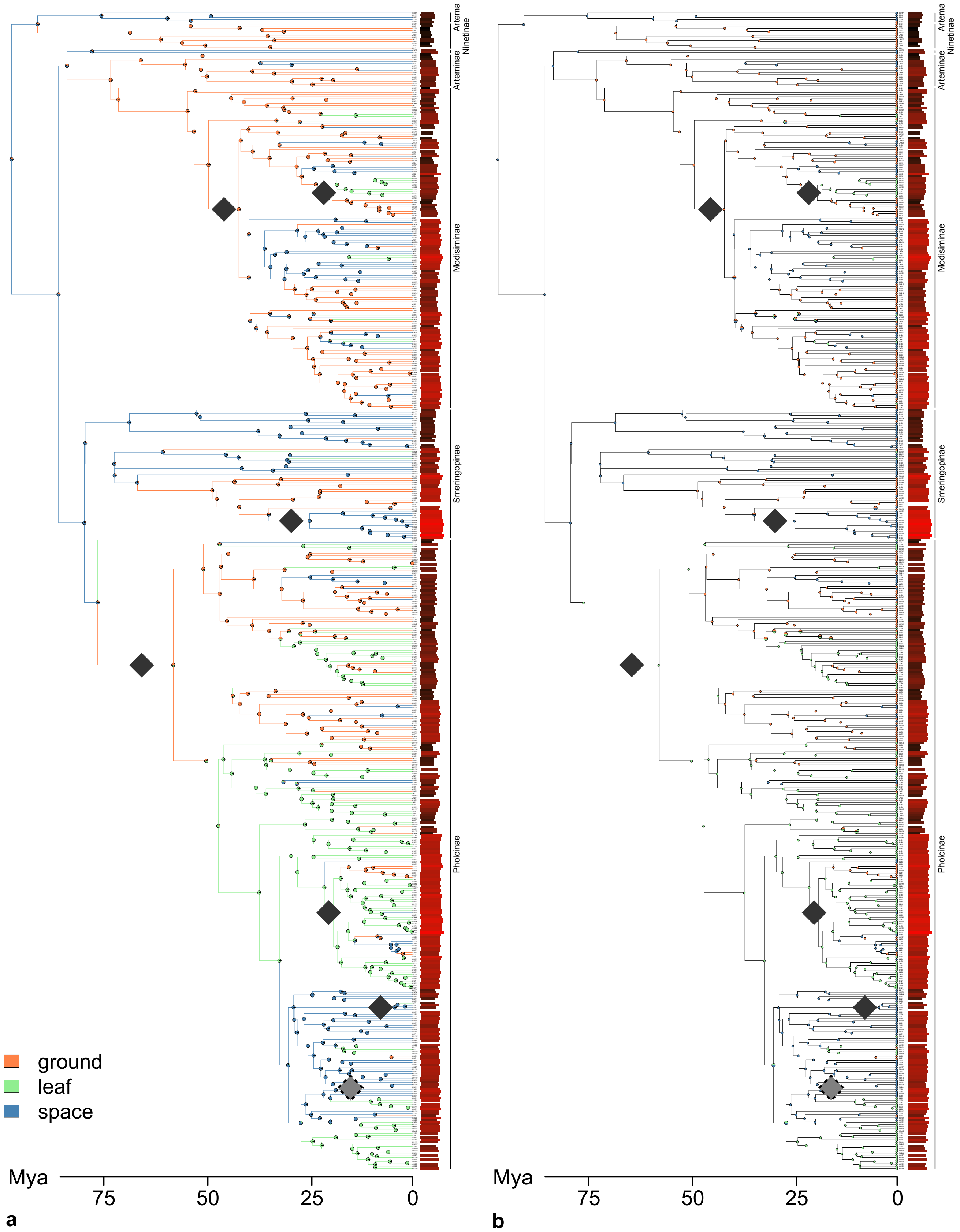

Figure S12: Time tree inferred with RelTime with ancestral states (a) inferred by maximum likelihood and (b) reconstructed by maximum parsimony. Pies at nodes in (a) depict ancestral state likelihoods while branch colors in code the most likely state. Pies at nodes in (b) give the most parsimonious state(s). Bars next to tips illustrate the ratio of metatarsus to tibia of the first leg which was used as a proxy of microhabitat. Diamonds show speciation rate shifts of the best fitting scenario inferred with BAMM. The light grey diamond was located on a branch which is not present in the current tree because no microhabitat preference data was available for the respective species (BAMM analyses did not use microhabitat information).

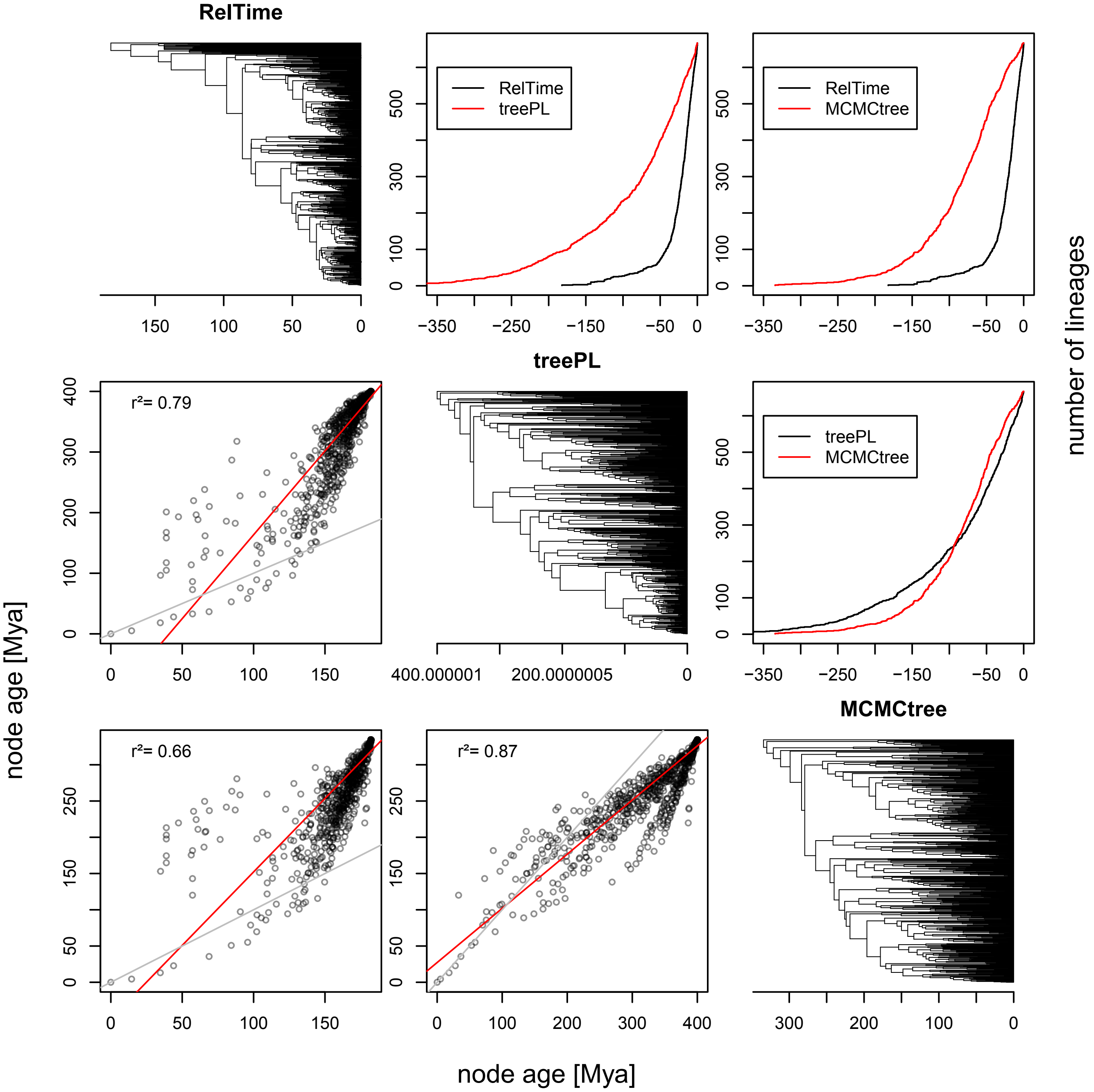

Figure S13: Comparison of divergence times inferred by the employed methods on the fixed topology of the most likely tree for the complete sampling (RAxML; Fig. S3). The lower triangle compares ages of each node. The red and grey lines are regression lines and the diagonal line (perfect match), respectively. The upper triangle compares lineages through time.

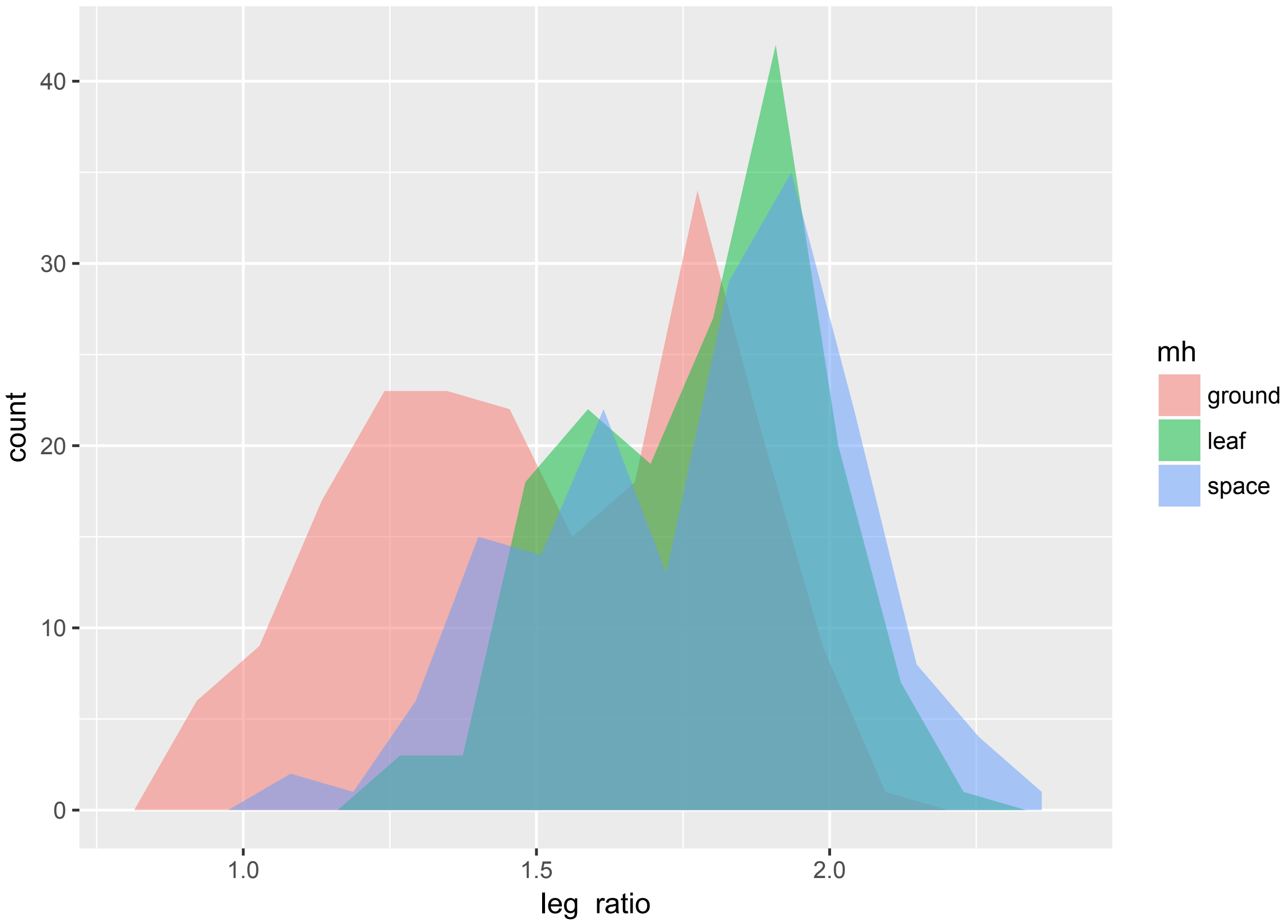

Figure S14: Distribution of the metatarsus to tibia ratio of the first leg by microhabitat. Leave dwellers and space living species appear to have higher ratios; however, bimodal distributions for all microhabitats might indicate also a phylogenetic component.

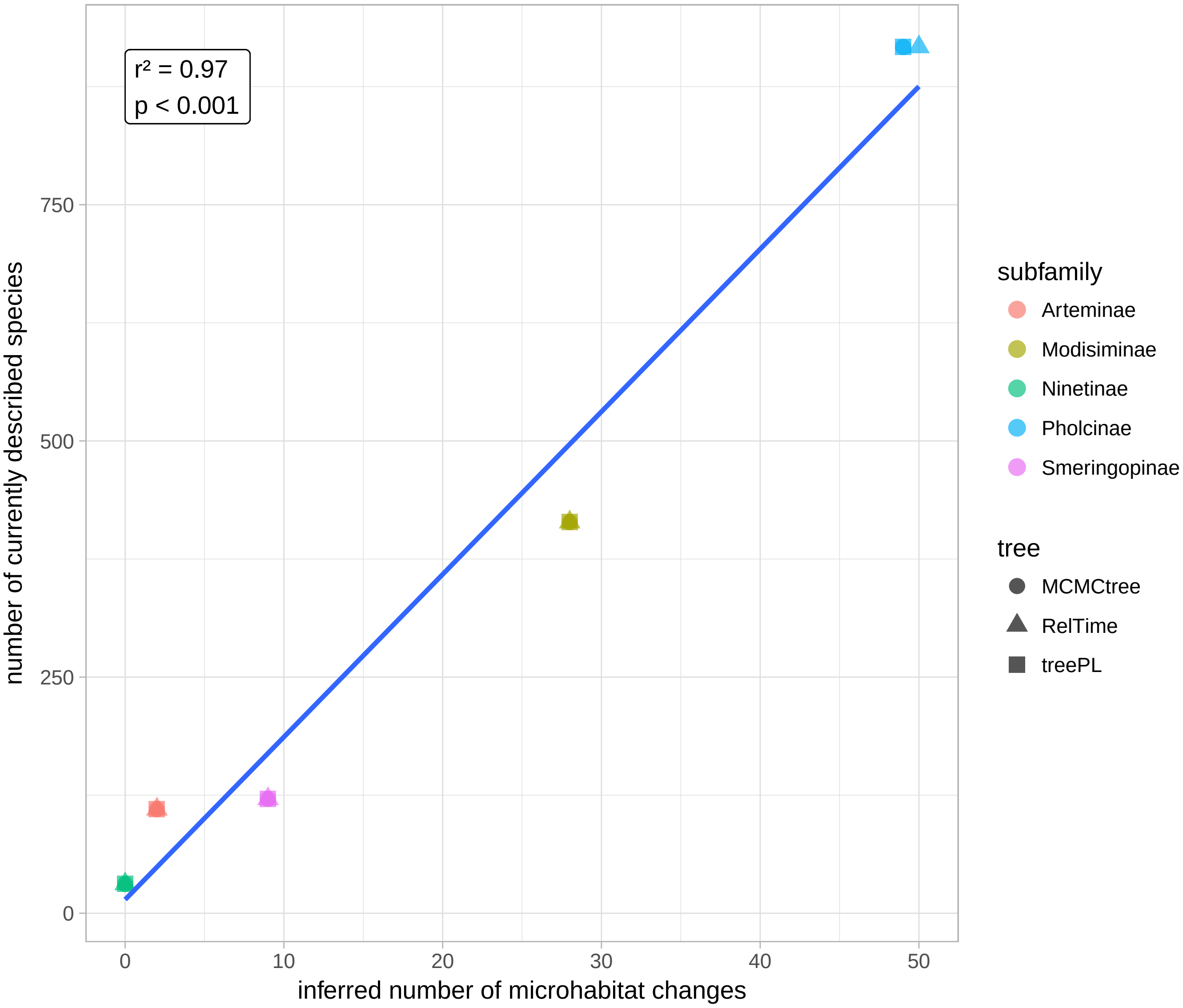

Figure S15: Number of microhabitat changes in subfamilies of pholcid spiders based on ancestral state inferences of all available time trees (complete sampling). Coefficient of determination and p-value of a linear regression are given in the upper left corner. The corresponding regression line is plotted in blue.

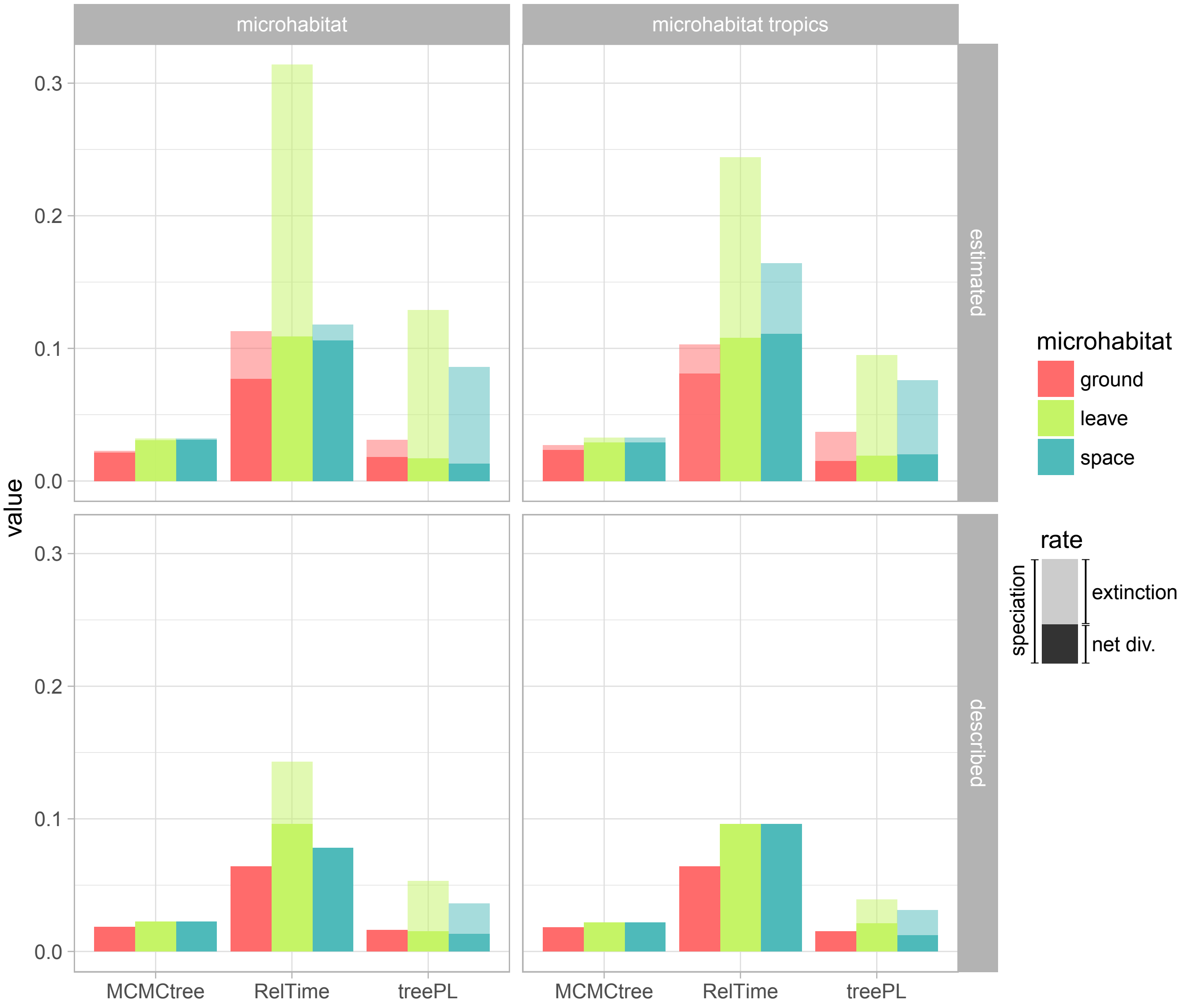

Figure S16: Diversification rates in dependence of microhabitat (inferred with MuSSE). Results based on currently described and of estimated species numbers are compared (rows) as well as rates inferred based on the complete sampling and based on species from tropical broad leave forest only (colums).

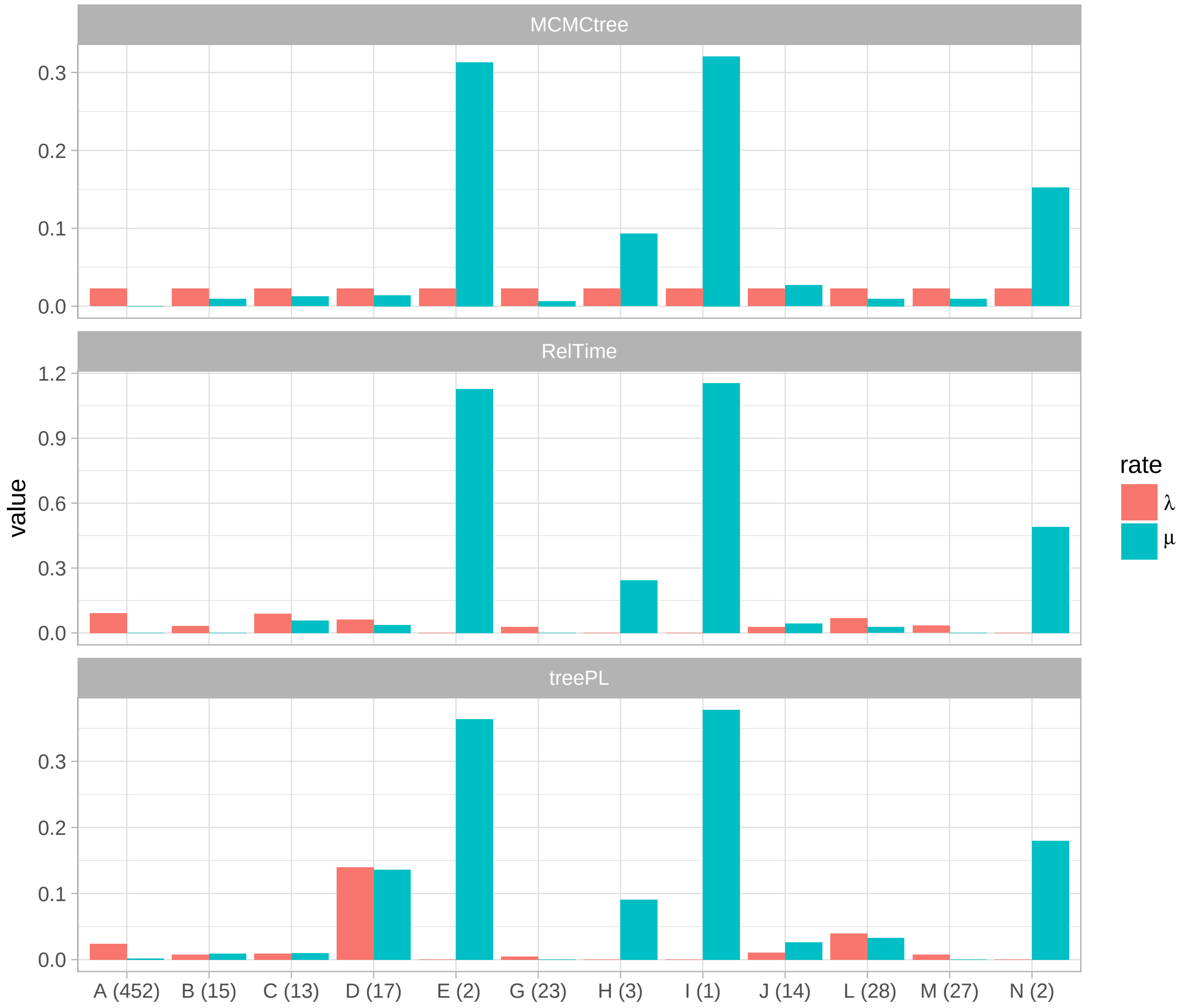

Figure S17: Diversification rates in dependence of biomes, inferred by MuSSE for all dated trees. Biomes are coded as follows:
A = Tropical & Subtropical Moist Broadleaf Forests,
B = Tropical & Subtropical Dry Broadleaf Forests,
C = Tropical & Subtropical Coniferous Forests,
D = Temperate Broadleaf & Mixed Forests,
E = Temperate Conifer Forests,
G = Tropical & Subtropical Grasslands, Savannas & Shrublands,
H = Temperate Grasslands, Savannas & Shrublands,
I = Flooded Grasslands & Savannas,
J = Montane Grasslands & Shrublands,
L = Mediterranean Forests, Woodlands & Scrub,
M = Deserts & Xeric Shrublands,
N = Mangroves.
Available specimen count per biome is given in parentheses at the x-axis.

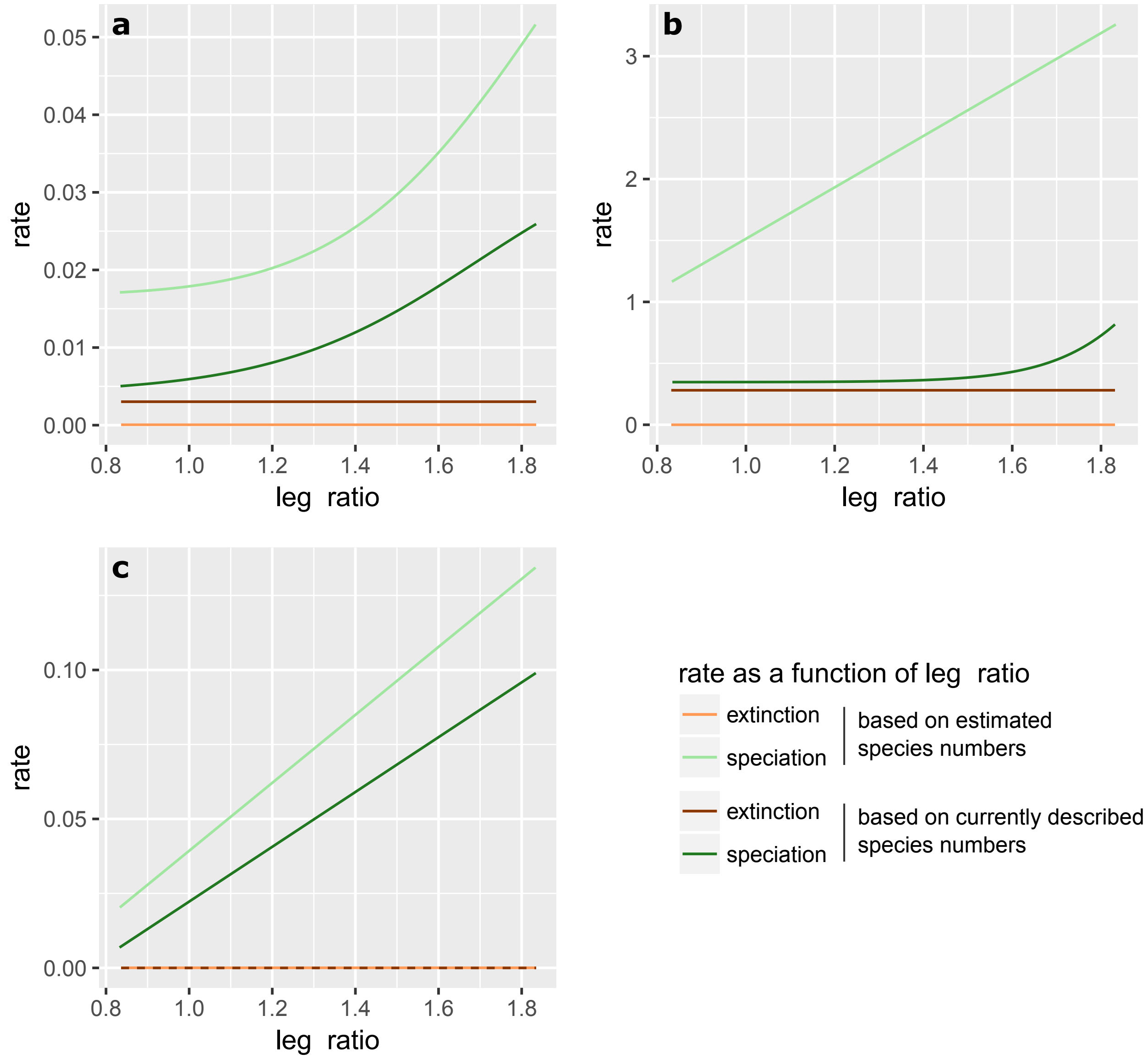

Figure S18: Diversification rates in dependence of the metatarsus to tibia ratio of the first leg (inferred with QuaSSE) using the dated tree resulting from (a) treePL, (b) MCMCtree, and (c) RelTime analyses.

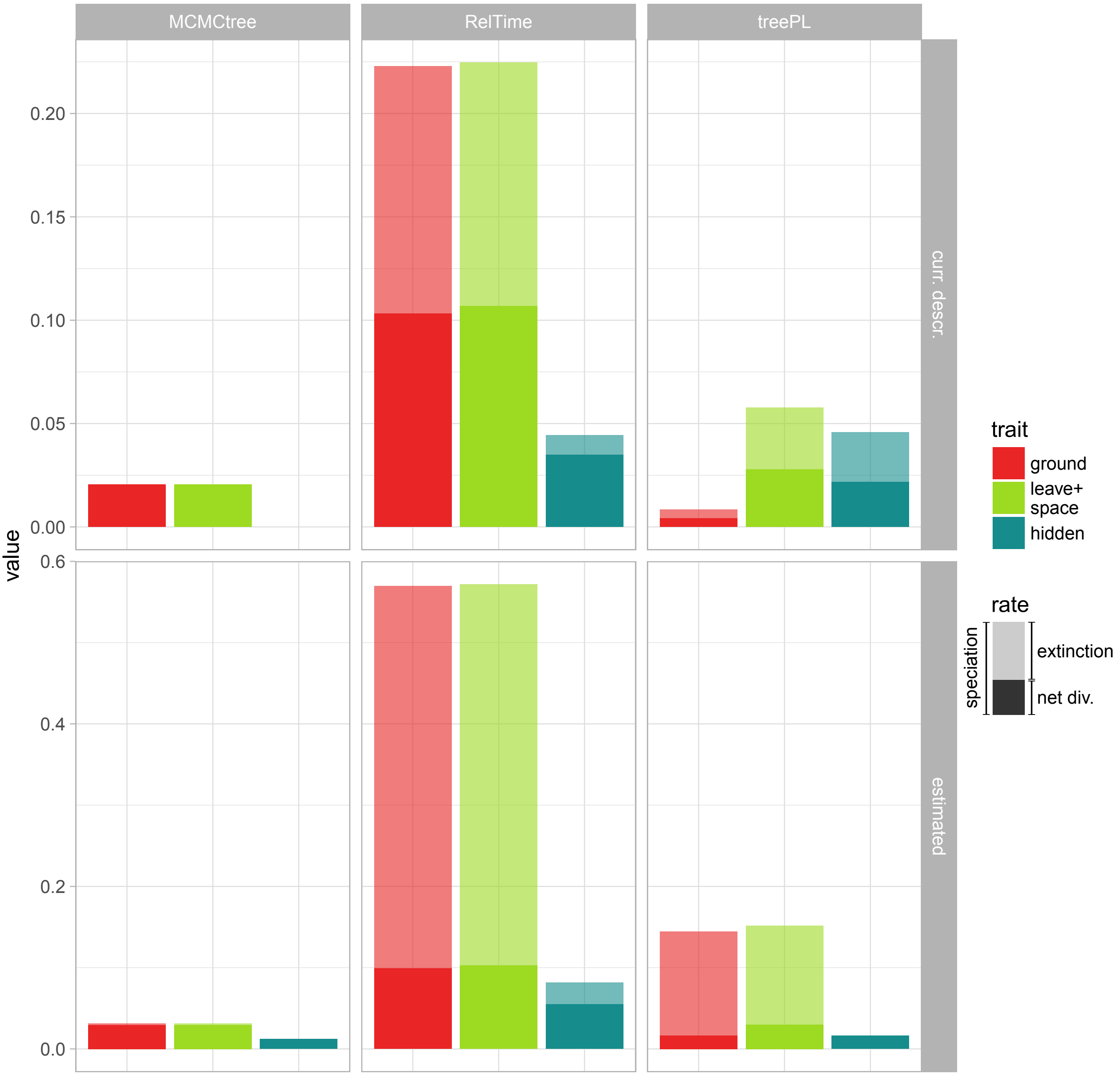

Figure S19: Diversification rates in dependence of microhabitat (inferred with HiSSE). Pooled leave- and space dwelling species were evaluated against ground living species. Results based on currently described and of estimated species numbers are compared (rows) among all dated trees (colums).

2 Supplementary Tables

Table S1: Table of accession numbers. The code which is also given in the trees, species names, GenBank accession numbers for all of the six loci if present, and ZFMK biobank accession numbers are given.

code	species	12S	16S	18S	28S	CO1	H3	biobank
S067	*Aetana abadae*			MG268047	MG268374		MG269086	ZFMK-DNA-FD04784097
S015	*Aetana baganihan*	MG267452		MG268044	MG268370		MG269076	ZFMK-DNA-FD04784085
S075	*Aetana banahaw*	MG267497	MG267738	MG268052	MG268379			ZFMK-DNA-FD04784153
GB01	*Aetana gaya*					MG268802		
S330	*Aetana Ind101*	MG267565	MG267863	MG268188	MG268542	MG268790	MG269081	ZFMK-DNA-FD04784318
S331	*Aetana Ind114*	MG267626	MG267881	MG268193	MG268492	MG268761	MG269085	ZFMK-DNA-FD04784326
S119	*Aetana indah*			MG268097	MG268405	MG268800	MG269080	ZFMK-DNA-FD04784131
S117	*Aetana kinabalu*	MG267498		MG268095	MG268403		MG269117	ZFMK-DNA-FD04784115
S221	*Aetana kiukoki*			MG268088	MG268392	MG268799		ZFMK-DNA-FD04784158
S223	*Aetana kiukoki*	MG267476		MG268089	MG268394	MG268798		ZFMK-DNA-FD04784174
GB04	*Aetana lambir*					MG268801		
S121	*Aetana lambir*			MG268098	MG268406			ZFMK-DNA-FD04784139
S080	*Aetana libjo*				MG268371		MG269075	ZFMK-DNA-FD04784098
GB31	*Aetana loboc*					MG268793		
S069	*Aetana loboc*		MG267736	MG268086	MG268377		MG269082	ZFMK-DNA-FD04784113
S077	*Aetana lozadae*		MG267739	MG268050	MG268378	MG268795	MG269084	ZFMK-DNA-FD04784074
GB32	*Aetana manansalai*					MG268796		
S073	*Aetana manansalai*		MG267737	MG268051	MG268381		MG269083	ZFMK-DNA-FD04784145
GB70	*Aetana ocampoi-morphA*					MG268791		
S007	*Aetana ocampoi-morphA*	MG267450		MG268046	MG268373		MG269078	ZFMK-DNA-FD04784116
S008	*Aetana ocampoi-morphB*	MG267451		MG268045	MG268372		MG269077	ZFMK-DNA-FD04784124
GB02	*Aetana omayan*					MG268794		
S001	*Aetana omayan*	MG267464	MG267735	MG268048	MG268375		MG269087	ZFMK-DNA-FD04784092
S079	*Aetana paragua*		MG267740	MG268049	MG268376	MG268797		ZFMK-DNA-FD04784090
S118	*Aetana poring*			MG268096	MG268404	MG268803	MG269079	ZFMK-DNA-FD04784123
OG26	*Agelenopsis aperta*		FJ607444	FJ607478	FJ607517	FJ607552	FJ607591	
S002	*Anansus atewa*			MG268035	MG268389		MG268969	ZFMK-DNA-FD04784100
S286	*Anansus ewe*	MG267543		MG268152	MG268466	MG268874		ZFMK-DNA-FD04784179
P0190	*Anansus kamwai*	JX023799		JX023985	JX024092	JX023572		ZFMK-DNA-100446434
S309	*Anopsicus Car127*		MG267843	MG268186	MG268481	MG268693		ZFMK-DNA-FD04784253
S310	*Anopsicus Car206*	MG267564	MG267862	MG268187	MG268482		MG269022	ZFMK-DNA-FD04784261
S413	*Anopsicus USA3*	MG267645	MG267936		MG268545	MG268677		ZFMK-DNA-FD04784405
OG7	*Araneus diadematus*		FJ607445	FJ607479	FJ607518	FJ607553	FJ607592	
OG34	*Argiope argentata*		FJ607446	FJ607480	FJ607519	FJ607554	FJ607593	
OG6	*Ariadna fidicina*		FJ607447	FJ607481	FJ607520	FJ607555	FJ607594	
BB01	*Artema atlanta*	AY560685	AY560663			AY560771		
JA119	*Artema atlanta*		DQ667748			DQ667854		ZFMK-DNA-FC17941390
S312	*Artema bunkpurugu*		MG267845	MG268183		MG268734		ZFMK-DNA-FD04784277
S326	*Artema doriai*		MG267860	MG268185		MG268738		ZFMK-DNA-FD04784294
GB03	*Artema nephilit*					MG268733		
S325	*Artema nephilit*		MG267859	MG268184				ZFMK-DNA-FD04784286
S497	*Artema Om14*	MG267717	MG268005	MG268334	MG268598	MG268735		ZFMK-DNA-FD05101751
S235	*Artema sp.C*		MG267783			MG268736		ZFMK-DNA-FD04784175
S287	*Artema sp.C*		MG267823	MG268182		MG268737		ZFMK-DNA-FD04784187
S332	*Artema transcaspica*	MG267589	MG267864	MG268244		MG268739		ZFMK-DNA-FD04784334
OG4	*Austrochilus sp*			FJ607483	FJ607522	FJ607557	FJ607596	
S414	*Aymaria Br16-188*	MG267646	MG268004	MG268261	MG268546	MG268805	MG268984	ZFMK-DNA-FD04784413
P0231	*Belisana akebona*	JX023821	JX023915	JX024018			JX023678	ZFMK-DNA-100446379
S238	*Belisana aninaj*			MG268121	MG268432			ZFMK-DNA-FD04784199
S415	*Belisana aninaj*	MG267647			MG268588	MG268876		ZFMK-DNA-FD04784421
S230	*Belisana australis*	MG267472		MG268091	MG268399	MG268868	MG269096	ZFMK-DNA-FD04784230
S333	*Belisana bohorok*	MG267627	MG267888	MG268197		MG268686	MG269097	ZFMK-DNA-FD04784342
S125	*Belisana Bor121*			MG268100	MG268407	MG268766	MG269109	ZFMK-DNA-FD04784155
S243	*Belisana Bor121*	MG267511	MG267789	MG268125	MG268436		MG269108	ZFMK-DNA-FD04784231
S126	*Belisana Bor147*			MG268101	MG268408			ZFMK-DNA-FD04784156
S127	*Belisana Bor152*		MG267786	MG268102	MG268409	MG268627	MG269100	ZFMK-DNA-FD04784164
S128	*Belisana Bor160*		MG267787	MG268103			MG269102	ZFMK-DNA-FD04784172
S129	*Belisana Bor85*	MG267499	MG267788				MG269101	ZFMK-DNA-FD04784180
S334	*Belisana Ind15*	MG267628	MG267883	MG268198		MG268636	MG269098	ZFMK-DNA-FD04784255
S237	*Belisana junkoae*	MG267468		MG268094	MG268474			ZFMK-DNA-FD04784191
S239	*Belisana ketambe?*	MG267508		MG268122	MG268433			ZFMK-DNA-FD04784207
S025	*Belisana Benj44*	MG267449	MG267742	MG268070			MG269095	ZFMK-DNA-FD04784062
S241	*Belisana khaoyai*	MG267509		MG268123	MG268434		MG269104	ZFMK-DNA-FD04784215
S242	*Belisana leuser*	MG267510		MG268124	MG268435	MG268833	MG269103	ZFMK-DNA-FD04784223
S244	*Belisana Mal30*	MG267512		MG268126		MG268825		ZFMK-DNA-FD04784239

code	species	12S	16S	18S	28S	CO1	H3	biobank
S416	*Belisana Mal35*	MG267648	MG267937	MG268262	MG268547			ZFMK-DNA-FD04784429
S246	*Belisana Mal55*		MG267790	MG268127	MG268437			ZFMK-DNA-FD04784247
S247	*Belisana Mal76*	MG267513	MG267791		MG268438	MG268823		ZFMK-DNA-FD04784160
S248	*Belisana Mal77*	MG267514	MG267792	MG268128	MG268439	MG268731	MG269094	ZFMK-DNA-FD04784168
S249	*Belisana Mal91*	MG267515	MG267793	MG268129		MG268623		ZFMK-DNA-FD04784176
S336	*Belisana nahtanoj*	MG267632	MG267884	MG268199	MG268494	MG268767	MG269105	ZFMK-DNA-FD04784271
S219	*Belisana Phi80*	MG267478			MG268391		MG269099	ZFMK-DNA-FD04784245
P0282	*Belisana Raff32*		JX023946	JX024058			JX023708	ZFMK-DNA-100437888
S417	*Belisana ranong*	MG267649		MG268263	MG268548	MG268804	MG269093	ZFMK-DNA-FD04784437
S386	*Belisana sabah*	MG267612	MG267901	MG268240	MG268520	MG268765	MG269107	ZFMK-DNA-FD04784291
S337	*Belisana sandakan*	MG267588		MG268189	MG268487			ZFMK-DNA-FD04784279
S498	*Belisana SL37*		MG268006	MG268335		MG268676		ZFMK-DNA-FD05101759
S103	*Belisana Tai4*	MG267484		MG268109		MG268904		ZFMK-DNA-FD04784122
S338	*Belisana tambligan*	MG267633	MG267885	MG268200	MG268495	MG268764	MG269106	ZFMK-DNA-FD04784287
P0267	*Buitinga batwa*	JX023838	JX023934	JX024046	JX024138		JX023698	ZFMK-DNA-100437896
S339	*Buitinga buhoma*	MG267629	MG267886	MG268208	MG268505	MG268952	MG269072	ZFMK-DNA-FD04784295
P0259	*Buitinga griswoldi*	JX023834		JX024040	JX024134		JX023690	ZFMK-DNA-100437913
P0191	*Buitinga nigrescens?*		JX023885	JX023986	JX024093	JX023573		ZFMK-DNA-100446422
S340	*Buitinga ruhiza*	MG267630		MG268209	MG268506	MG268953		ZFMK-DNA-FD04784303
S341	*Buitinga ruwenzori*	MG267631	MG267887	MG268210		MG268954	MG269071	ZFMK-DNA-FD04784311
S483	*Buitinga Uga5*	MG267702		MG268321	MG268581	MG268955	MG269073	ZFMK-DNA-FD04784387
S484	*Buitinga Uga78*	MG267703		MG268322	MG268582	MG268956	MG269074	ZFMK-DNA-FD04784395
P0156	*Buitinga wataita*	JX023769	JX023862	JX023959	JX024072	JX023552		ZFMK-DNA-100446448
S251	*Calapnita anai*	MG267516	MG267794					ZFMK-DNA-FD04784184
S418	*Calapnita bankirai*	MG267650	MG267938	MG268264		MG268923		ZFMK-DNA-FD04784350
S419	*Calapnita bario*	MG267651	MG267939			MG268849		ZFMK-DNA-FD04784358
S420	*Calapnita bidayuh*	MG267652	MG267940	MG268265		MG268924		ZFMK-DNA-FD04784366
S421	*Calapnita bohol*	MG267653	MG267941	MG268266	MG268549	MG268854		ZFMK-DNA-FD04784374
S133	*Calapnita deelemanae*	MG267500					MG269164	ZFMK-DNA-FD04784188
S485	*Calapnita dinagat*	MG267704	MG267991	MG268323	MG268583	MG268851		ZFMK-DNA-FD04784403
S184	*Calapnita kubah*					MG268920	MG269165	ZFMK-DNA-FD04784197
S136	*Calapnita lehi*	MG267503				MG268919	MG269163	ZFMK-DNA-FD04784212
S422	*Calapnita loksado*	MG267654	MG267942	MG268267	MG268550	MG268853		ZFMK-DNA-FD04784382
S227	*Calapnita nunezae*			MG268090	MG268397	MG268850		ZFMK-DNA-FD04784206
S134	*Calapnita phyllicola*	MG267501				MG268922		ZFMK-DNA-FD04784196
S252	*Calapnita saluang*	MG267517		MG268130	MG268440			ZFMK-DNA-FD04784192
GB33	*Calapnita semengoh*					MG268921		
S135	*Calapnita semengoh*	MG267502					MG269162	ZFMK-DNA-FD04784204
S229	*Calapnita subphyllicola*		MG267800			MG268918		ZFMK-DNA-FD04784222
S076	*Calapnita vermiformis*				MG268380			ZFMK-DNA-FD04784066
S424	*Calapnita vermiformis?*	MG267655	MG267943	MG268268	MG268551	MG268852		ZFMK-DNA-FD04784390
OG37	*Callobius sp*		FJ607450	FJ607485	FJ607524	FJ607559	FJ607598	
S344	*Carapoia alagoas*	MG267566	MG267865	MG268190	MG268488	MG268784	MG269039	ZFMK-DNA-FD04784335
S352	*Carapoia Br15-15*	MG267567	MG267866	MG268191	MG268489	MG268744	MG269025	ZFMK-DNA-FD04784304
S455	*Carapoia Br15-18*			MG268296	MG268594	MG268656	MG269029	ZFMK-DNA-FD04784353
S392	*Carapoia Br15-48*	MG267622	MG267907	MG268239	MG268516	MG268778	MG269027	ZFMK-DNA-FD04784339
S354	*Carapoia Br15-62*	MG267639	MG267893	MG268205	MG268501	MG268782	MG269050	ZFMK-DNA-FD04784320
S394	*Carapoia Br15-78*	MG267592	MG267918	MG268213	MG268522	MG268768		ZFMK-DNA-FD04784348
S393	*Carapoia Br15-79*	MG267623	MG267908	MG268238	MG268517	MG268777	MG269028	ZFMK-DNA-FD04784347
S428	*Carapoia Br16-58*	MG267659	MG267947	MG268333	MG268554	MG268770	MG269051	ZFMK-DNA-FD04784422
S429	*Carapoia Br16-67*	MG267660	MG267948	MG268271	MG268555	MG268779	MG269026	ZFMK-DNA-FD04784430
S425	*Carapoia Br16-73*	MG267656	MG267944	MG268269	MG268552	MG268769	MG269049	ZFMK-DNA-FD04784398
S035	*Carapoia brescoviti*	MG267433	MG267743	MG268059	MG268360		MG269041	ZFMK-DNA-FD04784142
GB34	*Carapoia capixaba*					MG268776		
S027	*Carapoia capixaba*	MG267494	MG267747	MG268087	MG268354		MG269035	ZFMK-DNA-FD04784078
S345	*Carapoia carvalhoi*	MG267636	MG267892	MG268204	MG268499	MG268786	MG269042	ZFMK-DNA-FD04784343
S040	*Carapoia carybei*	MG267429	MG267746	MG268057		MG268772	MG269044	ZFMK-DNA-FD04784087
S346	*Carapoia crasto*	MG267637	MG267891	MG268206	MG268498	MG268789	MG269037	ZFMK-DNA-FD04784256
GB05	*Carapoia dandarae*					MG268757		
S033	*Carapoia dandarae*		MG267753	MG268056	MG268356		MG269047	ZFMK-DNA-FD04784126
P0209	*Carapoia divisa*	JX023809	JX023900		JX024108	JX023585	JX023660	ZFMK-DNA-100446405
S426	*Carapoia fowleri*	MG267657	MG267945		MG268589	MG268780	MG269023	ZFMK-DNA-FD04784406
P0205	*Carapoia genitalis*	JX023807	JX023897	JX023997	JX024105	JX023582	JX023656	ZFMK-DNA-100446401
GB35	*Carapoia gracilis*					MG268773		
S030	*Carapoia gracilis*		MG267751	MG268063			MG269031	ZFMK-DNA-FD04784102
S343	*Carapoia jiboja*	MG267638	MG267890	MG268203	MG268497	MG268755	MG269045	ZFMK-DNA-FD04784327
P0206	*Carapoia macacu*		JX023898	JX023998		JX023583	JX023657	ZFMK-DNA-100446402
P0207	*Carapoia macacu*		JX023899	JX023999	JX024106		JX023658	ZFMK-DNA-100446403
S037	*Carapoia marceloi*	MG267428	MG267754	MG268055	MG268355	MG268756	MG269046	ZFMK-DNA-FD04784063
S026	*Carapoia mirim*		MG267749	MG268060	MG268357	MG268774	MG269032	ZFMK-DNA-FD04784070
P0211	*Carapoia nairae*	JX023811				JX023587	JX023662	ZFMK-DNA-100446407
S427	*Carapoia ocaina*	MG267658	MG267946	MG268270	MG268553	MG268781	MG269024	ZFMK-DNA-FD04784414
BB02	*Carapoia paraguaensis*	AY560723	AY560680			AY560771		
JA97	*Carapoia paraguaensis*		DQ667749		DQ667839	DQ667855		ZFMK-DNA-FC17941404
S028	*Carapoia patafina*		MG267748	MG268085	MG268358	MG268775	MG269033	ZFMK-DNA-FD04784086

code	species	12S	16S	18S	28S	CO1	H3	biobank
S029	*Carapoia pau*	MG267495	MG267750	MG268064			MG269030	ZFMK-DNA-FD04784094
S031	*Carapoia rheimsae*	MG267431	MG267744	MG268061	MG268361	MG268788	MG269036	ZFMK-DNA-FD04784110
S288	*Carapoia RJ16*	MG267544	MG267824	MG268153	MG268467	MG268787	MG269034	ZFMK-DNA-FD04784195
S342	*Carapoia septentrionalis*	MG267635	MG267889	MG268202	MG268496	MG268785	MG269040	ZFMK-DNA-FD04784319
JA102	*Carapoia ubatuba*		DQ667750		DQ667840	DQ667856		ZFMK-DNA-FC17941444
S036	*Carapoia una*	MG267427	MG267755	MG268054		MG268758	MG269048	ZFMK-DNA-FD04784150
S038	*Carapoia viridis*	MG267430	MG267745	MG268062	MG268353	MG268783	MG269038	ZFMK-DNA-FD04784071
P0208	*Carapoia voltavelha*	JX023808		JX024000	JX024107	JX023584	JX023659	ZFMK-DNA-100446404
S034	*Carapoia zumbii*	MG267432	MG267752	MG268058	MG268359	MG268680	MG269043	ZFMK-DNA-FD04784134
S060	*Chibchea mapuche?*	MG267436		MG268074	MG268363			ZFMK-DNA-FD04784144
S058	*Chibchea picunche?*	MG267435		MG268073	MG268362			ZFMK-DNA-FD04784128
S430	*Chisosa diluta*	MG267661		MG268272	MG268556	MG268626		ZFMK-DNA-FD04784438
BB03	*Ciboneya antraia*	AY560687	AY560665		AY560732	AY560794		
S313	*Ciboneya nuriae*		MG267846					ZFMK-DNA-FD04784285
S289	*'Coryssocnemis' iviei*	MG267545	MG267825	MG268154	MG268468	MG268619	MG269147	ZFMK-DNA-FD04784203
JA100	*Coryssocnemis simla*		DQ667753			DQ667858		ZFMK-DNA-FC17941428
JA107	*Coryssocnemis simla*	MG267466	DQ667752			DQ667859		ZFMK-DNA05-JA107
P0154	*Crossopriza lyoni*	JX023767	JX023860	JX023957	JX024070	JX023551	JX023619	ZFMK-DNA-100446459
SB026	*Crossopriza lyoni*	MG267734				MG268895		
S499	*Crossopriza Om16*	MG267718	MG268017	MG268336	MG268599	MG268821	MG269116	ZFMK-DNA-FD05101767
S500	*Crossopriza Om20*	MG267719	MG268007	MG268337	MG268600	MG268820	MG269115	ZFMK-DNA-FD05101775
S290	*Crossopriza pristina*	MG267546	MG267826	MG268155	MG268469	MG268894		ZFMK-DNA-FD04784211
OG9	*Deinopis spinosa*		FJ525351	FJ525388	FJ525370	FJ525318	FJ525337	
OG35	*Diguetia sp*	AY560724	AY560681		AY560766	AY560795		
OG20	*Dysdera erythrina-fervida*		GQ285606		GQ285615	GQ285630	GQ285625	
OG19	*Dysderocrates silvestris*		AF244151			AF244236	EU139755	
OG22	*Erigone dentosa*			FJ607489	FJ607628	FJ607563		
OG1	*Euagrus chisoseus*		FJ607454	FJ607490	FJ607529	FJ607564	FJ607602	
S323	*Gen. Cu12-325*		MG267855	MG268178				ZFMK-DNA-FD04784270
S329	*Gen.n. Br15-159*		MG267861	MG268180	MG268486	MG268614		ZFMK-DNA-FD04784310
S353	*Gen.n. Br15-45*	MG267642	MG267897	MG268211		MG268687	MG269014	ZFMK-DNA-FD04784312
S433	*Gen.n. Br16-178*	MG267663		MG268275		MG268641	MG268999	ZFMK-DNA-FD04784367
S431	*Gen.n. Br16-196*		MG267949	MG268273		MG268646	MG268991	ZFMK-DNA-FD04784351
S464	*Gen.n. Br16-44*		MG267978	MG268305	MG268571	MG268620	MG269110	ZFMK-DNA-FD04784425
S432	*Gen.n. Br16-50*	MG267662		MG268274		MG268657	MG268986	ZFMK-DNA-FD04784359
S006	*Gen.n. Geneve59*	MG267438		MG268066	MG268384		MG268985	ZFMK-DNA-FD04784108
S468	*Gen.n. Ind206*	MG267689	MG267982	MG268309	MG268574	MG268732	MG269065	ZFMK-DNA-FD04784362
S357	*Gen.n. MACN270*		MG267896	MG268201	MG268504	MG268621	MG269186	ZFMK-DNA-FD04784344
S358	*Gen.n. MACN273*	MG267610	MG267917	MG268229	MG268508	MG268618	MG268978	ZFMK-DNA-FD04784257
S501	*Gen.n. Om6*	MG267720	MG268008	MG268338	MG268601	MG268615	MG268968	ZFMK-DNA-FD05101783
JA83	*Gen.n. Ven01*				DQ667847	DQ667903		ZFMK-DNA-FC17941387
S059	*Gertschiola macrostyla*	MG267465		MG268038	MG268364			ZFMK-DNA-FD04784136
S434	*Gertschiola macrostyla*	MG267664	MG267950	MG268276	MG268557	MG268725	MG268967	ZFMK-DNA-FD04784375
S347	*Guaranita yaculica?*	MG267643		MG268194	MG268503	MG268616	MG268970	ZFMK-DNA-FD04784264
S122	*Hantu kapit*			MG268099	MG268413	MG268896	MG269136	ZFMK-DNA-FD04784147
S412	*Hantu kapit*	MG267571		MG268246		MG268897	MG269137	ZFMK-DNA-FD04784397
S322	*Hantu niah*			MG268181		MG268651	MG269064	ZFMK-DNA-FD04784262
OG10	*Harpactea hombergi*		AF244148		EU139820	AF244233	EU139752	
OG36	*Harpactocrates radulifer*				EU139821	AF244235	EU139753	
OG18	*Herennia multipuncta*	EU003236	EU003260	EU003386	EU003433	EU003288	EU003320	
S348	*Holocneminus huangdi*	MG267570	MG267879	MG268245	MG268534	MG268672	MG268998	ZFMK-DNA-FD04784272
S486	*Holocneminus Ind73*	MG267705	MG267992	MG268324		MG268713	MG269123	ZFMK-DNA-FD04784411
S253	*Holocneminus multiguttatus*	MG267518	MG267795	MG268131	MG268441			ZFMK-DNA-FD04784200
S254	*Holocneminus multiguttatus*	MG267519	MG267796	MG268132	MG268442		MG269124	ZFMK-DNA-FD04784208
S016	*Holocneminus Phi105*	MG267463		MG268067		MG268714		ZFMK-DNA-FD04784093
S078	*Holocneminus Phi74*		MG267756	MG268068		MG268712	MG269125	ZFMK-DNA-FD04784082
S068	*Holocneminus Phi9*		MG267757	MG268069			MG269126	ZFMK-DNA-FD04784105
S435	*Holocnemus caudatus*	MG267665	MG267951	MG268277	MG268590	MG268890	MG269111	ZFMK-DNA-FD04784383
P0233	*Holocnemus hispanicus*			JX024020		JX023600	JX023680	ZFMK-DNA-100446113
S436	*Hoplopholcus asiaeminoris*	MG267666	MG267952	MG268278		MG268813		ZFMK-DNA-FD04784391
S009	*Hoplopholcus cecconii*	MG267447		MG268084		MG268811		ZFMK-DNA-FD04784132
S437	*Hoplopholcus Del5*	MG267667	MG267953	MG268279		MG268814		ZFMK-DNA-FD04784399
S057	*Hoplopholcus forskali*	MG267446	MG267758	MG268083				ZFMK-DNA-FD04784120
S316	*Hoplopholcus labyrinthi*	MG267560	MG267849	MG268172		MG268808		ZFMK-DNA-FD04784309
S438	*Hoplopholcus Mar66*	MG267668	MG267954	MG268280		MG268815		ZFMK-DNA-FD04784407
S315	*Hoplopholcus minotaurinus*	MG267559	MG267848	MG268171				ZFMK-DNA-FD04784301
S314	*Hoplopholcus minous*	MG267558	MG267847	MG268170				ZFMK-DNA-FD04784293
S439	*Hoplopholcus patrizii*		MG267955	MG268281		MG268807		ZFMK-DNA-FD04784415
S440	*Hoplopholcus Tur21*	MG267669	MG267956	MG268282		MG268812		ZFMK-DNA-FD04784423
S441	*Hoplopholcus Tur32*	MG267670	MG267957	MG268283		MG268809	MG269183	ZFMK-DNA-FD04784431

code	species	12S	16S	18S	28S	CO1	H3	biobank
S442	*Hoplopholcus Tur7*	MG267671	MG267958	MG268284	MG268558	MG268810		ZFMK-DNA-FD04784439
OG27	*Hyptiotes gertschi*		FJ607455	FJ607492	FJ607531	FJ607566	FJ607604	
S443	*Ibotyporanga Br16-149*	MG267672	MG267959	MG268285	MG268559	MG268742	MG268975	ZFMK-DNA-FD04784352
JA123	*Ibotyporanga naideae*		DQ667837		DQ667843	DQ667852		ZFMK-DNA-FC17941422
Ixc29	*Ixchela abernathyi*	MG267480	KF178420		MG268421	KF150114		ZFMK-DNA-FD04784060
Ixc51	*Ixchela santibanezi*	MG267481	KF178432	MG268104	MG268422	KF150126	MG268979	ZFMK-DNA-FD04784076
Ixc39	*Ixchela taxco*		KF178395			KF150089		ZFMK-DNA-FD04784068
Ixc56	*Ixchela viquezi*		KF178434			KF150128		ZFMK-DNA-FD04784084
S328	*Kambiwa neotropica*				MG268485	MG268617		ZFMK-DNA-FD04784302
S318	*Khorata circularis*		MG267851	MG268174		MG268730	MG269122	ZFMK-DNA-FD04784325
S319	*Khorata dupla*		MG267852	MG268175		MG268763	MG269133	ZFMK-DNA-FD04784333
P0223	*Khorata khammouan*	JX023818		JX024011		JX023596	JX023672	ZFMK-DNA-100446390
OG24	*Kukulcania hibernalis*	AY560726	AY560684		AY560764	AY560796		
S255	*Leptopholcus borneensis*	MG267520			MG268443		MG269141	ZFMK-DNA-FD04784216
GB36	*Leptopholcus budongo*					MG268729		
P0260	*Leptopholcus budongo*			JX024041	JX024135		JX023691	ZFMK-DNA-100437914
P0237	*Leptopholcus cf griswoldi*		JX023919	JX024024	JX024125			ZFMK-DNA-100433834
P0176	*Leptopholcus dschang*	JX023787		JX023973	JX024082	JX023562	JX023636	ZFMK-DNA-100446437
P0192	*Leptopholcus dschang*	JX023800	JX023886	JX023987	JX024094		JX023646	ZFMK-DNA-100446421
GB07	*Leptopholcus gabonicus*					MG268727		
P0160	*Leptopholcus gracilis*	JX023773		JX023962	JX024076	JX023554	JX023622	ZFMK-DNA-100446452
P0220	*Leptopholcus gracilis?*		JX023908	JX024008	JX024114		JX023671	ZFMK-DNA-100446393
P0150	*Leptopholcus guineensis*	JX023763	JX023856	JX023955	JX024068	JX023548	JX023615	ZFMK-DNA-100446463
SB070	*Leptopholcus kandy*	MG267729	MG268019	MG268346			MG269142	
SB071	*Leptopholcus kandy*	MG267730	MG268020	MG268347			MG269143	
GB08	*Leptopholcus kintampo*					MG268728		
P0235	*Leptopholcus MRAC530*		JX023918	JX024022	JX024123			ZFMK-DNA-100446383
P0279	*Leptopholcus podophthalmus*			JX024055			JX023707	ZFMK-DNA-100437885
S349	*Leptopholcus podophthalmus*	MG267572	MG267867	MG268248	MG268491	MG268752	MG269140	ZFMK-DNA-FD04784280
SB068	*Leptopholcus podophthalmus*	MG267728	MG268018	MG268345		MG268753		
P0146	*Leptopholcus tipula*		JX023853	JX023954	JX024067	JX023545	JX023611	ZFMK-DNA-100446467
P0169	*Leptopholcus tipula*			JX023970	JX024079	JX023560	JX023629	ZFMK-DNA-100446444
P0236	*Leptopholcus tipula*			JX024023	JX024124	JX023602	JX023682	ZFMK-DNA-100446384
P0261	*Leptopholcus tipula*		JX023929				JX023692	ZFMK-DNA-100437902
OG11	*Leucauge venusta_B*		FJ607457	FJ607494	FJ607533	FJ607568	FJ607606	
OG30	*Linyphia triangularis_A*	EU003239	AY078664		EU003410	EU003292	AY078702	
S351	*Litoporus Br15-107*	MG267641	MG267895	MG268196		MG268640	MG268995	ZFMK-DNA-FD04784296
S444	*Litoporus Br16-153*	MG267673	MG267960			MG268697	MG268996	ZFMK-DNA-FD04784360
S445	*Litoporus Br16-62*	MG267674	MG267961	MG268286	MG268560	MG268698	MG268997	ZFMK-DNA-FD04784368
S446	*Litoporus dimona*	MG267675	MG267962	MG268287	MG268561	MG268692	MG268976	ZFMK-DNA-FD04784376
P0212	*Litoporus iguassuensis*	JX023812	JX023902			JX023588	JX023663	ZFMK-DNA-100446408
P0213	*Litoporus iguassuensis*	JX023813	JX023903				JX023664	ZFMK-DNA-100446409
S447	*Litoporus manu*		MG267963	MG268288	MG268562	MG268705	MG268990	ZFMK-DNA-FD04784384
OG17	*Loxosceles laeta*		EU817794		GQ279182	GQ279223		
S448	*Mecolaesthus arima*	MG267676	MG267964	MG268289	MG268597	MG268675	MG269015	ZFMK-DNA-FD04784392
JA89	*Mecolaesthus longissimus*	AY560696	DQ667756		AY560736	DQ667861		
S294	*Mecolaesthus nigrifrons?*		MG267829	MG268158	MG268471		MG269016	ZFMK-DNA-FD04784235
S449	*Mecolaesthus taino?*		MG267965	MG268290	MG268563	MG268688	MG269017	ZFMK-DNA-FD04784400
S295	*Mecolaesthus Ven02/80-12*	MG267549	MG267830	MG268159	MG268472	MG268632		ZFMK-DNA-FD04784243
S450	*Mecolaesthus yawaperi*	MG267677		MG268291	MG268564	MG268650		ZFMK-DNA-FD04784408
BB09a	*Mesabolivar aurantiacus*	AY560695	AY560669		AY560735	AY560778		
S388	*Mesabolivar azureus*	MG267618	MG267912	MG268231	MG268512		MG269002	ZFMK-DNA-FD04784307
GB09	*Mesabolivar bonita*					MG268695		
S487	*Mesabolivar Br11-10*	MG267706	MG267993	MG268325	MG268584	MG268696	MG269010	ZFMK-DNA-FD04784419
S350	*Mesabolivar Br14-25*	MG267640	MG267894	MG268207	MG268500	MG268643	MG269008	ZFMK-DNA-FD04784288
S390	*Mesabolivar Br14-26*	MG267619	MG267914	MG268233	MG268514	MG268684	MG269005	ZFMK-DNA-FD04784323
S391	*Mesabolivar Br14-30*	MG267620	MG267915	MG268234	MG268515	MG268683	MG269006	ZFMK-DNA-FD04784331
S389	*Mesabolivar Br14-4*	MG267621	MG267913	MG268232	MG268513	MG268674	MG269003	ZFMK-DNA-FD04784315
S305	*Mesabolivar Br14-55*		MG267839	MG268167		MG268690	MG269007	ZFMK-DNA-FD04784316
S451	*Mesabolivar Br16-138*	MG267678	MG267966	MG268292	MG268565	MG268648		ZFMK-DNA-FD04784416
S452	*Mesabolivar Br16-32*	MG267679	MG267967	MG268293	MG268591	MG268673	MG269012	ZFMK-DNA-FD04784424
S453	*Mesabolivar Br16-60*		MG267968	MG268294	MG268592	MG268649		ZFMK-DNA-FD04784432
BB10	*Mesabolivar brasiliensis*	AY560698			AY560738	AY560780		
S456	*Mesabolivar cambridgei*	MG267681	MG267970	MG268297		MG268771	MG269052	ZFMK-DNA-FD04784361
JA50	*Mesabolivar camussi*		DQ667769			DQ667875		ZFMK-DNA-FC17941320
JA10	*Mesabolivar cavicelatus*		DQ667772			DQ667880		ZFMK-DNA-FC17941285
S395	*Mesabolivar charrua*	MG267593	MG267919		MG268523	MG268681	MG269011	ZFMK-DNA-FD04784356

code	species	12S	16S	18S	28S	CO1	H3	biobank
JA52	*Mesabolivar cyaneomaculatus*					DQ667865		ZFMK-DNA-FC17941336
BB11	*Mesabolivar cyaneotaeniatus*	AY560699	AY560671		AY560739	AY560781		
JA57	*Mesabolivar cyaneotaeniatus*		DQ667760			DQ667866		ZFMK-DNA-FC17941369
JA34	*Mesabolivar eberhardi*		DQ667763			DQ667870		ZFMK-DNA-FC17941287
JA93	*Mesabolivar eberhardi*		DQ667764		DQ667845	DQ667872		ZFMK-DNA-FC17941372
JA53	*Mesabolivar gabettae*		DQ667775			DQ667882		ZFMK-DNA-FC17941344
S410	*Mesabolivar goitaca*	MG267608	MG267921	MG268225	MG268525	MG268682	MG269009	ZFMK-DNA-FD04784381
S454	*Mesabolivar huambisa?*	MG267680	MG267969	MG268295	MG268593	MG268647		ZFMK-DNA-FD04784440
S396	*Mesabolivar huberi*	MG267594	MG267922	MG268214	MG268524	MG268685	MG269004	ZFMK-DNA-FD04784364
P0215	*Mesabolivar itapoa*	JX023814	JX023905	JX024003	JX024111	JX023590	JX023666	ZFMK-DNA-100446398
S387	*Mesabolivar kathrinae*	MG267617	MG267911	MG268230	MG268511	MG268639	MG269001	ZFMK-DNA-FD04784299
JA41	*Mesabolivar luteus*		DQ667766		DQ667846	DQ667873		ZFMK-DNA-FC17941343
JA19	*Mesabolivar mairyara*		DQ667770			DQ667876		ZFMK-DNA-FC17941357
GB10	*Mesabolivar pau*					MG268694		
GB11	*Mesabolivar sai*					MG268629		
S397	*Mesabolivar spinulosus*	MG267595	MG267920			MG268637	MG269000	ZFMK-DNA-FD04784372
JA29	*Mesabolivar tamoio*		DQ667767			DQ667874		ZFMK-DNA-FC17941342
JA54	*Mesabolivar togatus*					DQ667884		ZFMK-DNA-FC17941352
P0217	*Mesabolivar unicornis*	JX023816		JX024005		JX023592	JX023668	ZFMK-DNA-100446396
BB07	*Mesabolivar yuruani*	AY560694	AY560668					
JA101	*Mesabolivar yuruani*		DQ667754		DQ667844	DQ667860		ZFMK-DNA-FC17941436
S398	*Metagonia argentinensis*	MG267596	MG267924	MG268215		MG268715	MG268981	ZFMK-DNA-FD04784380
JA78	*Metagonia belize*		DQ667778					ZFMK-DNA-FC17941442
S457	*Metagonia beni*	MG267682	MG267971	MG268298		MG268884		ZFMK-DNA-FD04784369
P0198	*Metagonia bifida?*		JX023891	JX023991	JX024099	JX023577		ZFMK-DNA-100446415
JA12	*Metagonia Br03/80-25*		DQ667786			DQ667892		ZFMK-DNA-FC17941301
BB13	*Metagonia Br03/80-25*	AY560702			AY560741	AY560783		
P0199	*Metagonia Br07/80-1*		JX023892	JX023992	JX024100	JX023578	JX023650	ZFMK-DNA-100446414
P0277	*Metagonia Br09-2*		JX023942	JX024053	JX024142	JX023605	JX023705	ZFMK-DNA-100437883
P0197	*Metagonia Br09-4*			JX023990	JX024098	JX023576		ZFMK-DNA-100446416
JA27	*Metagonia Br09-52*		DQ667890			DQ667890		ZFMK-DNA-FC17941326
JA11	*Metagonia Br09-55*		DQ667785			DQ667891		ZFMK-DNA-FC17941293
S488	*Metagonia Br10-38*	MG267707	MG267994	MG268326	MG268585	MG268873	MG268980	ZFMK-DNA-FD04784427
S489	*Metagonia Br11-11*	MG267708	MG267995	MG268327	MG268586	MG268726	MG268982	ZFMK-DNA-FD04784435
S458	*Metagonia Br15-101*		MG267972	MG268299	MG268566	MG268762	MG269128	ZFMK-DNA-FD04784377
S459	*Metagonia Br16-125*		MG267973	MG268300	MG268595	MG268875	MG268983	ZFMK-DNA-FD04784385
S460	*Metagonia Br16-180*		MG267974	MG268301	MG268567	MG268817		ZFMK-DNA-FD04784393
S490	*Metagonia Br16-205*	MG267709	MG267996	MG268328	MG268587	MG268892	MG269129	ZFMK-DNA-FD04784443
S461	*Metagonia Br16-216*	MG267683	MG267975	MG268302	MG268568	MG268893	MG269130	ZFMK-DNA-FD04784401
JA117	*Metagonia delicata*		DQ667780					ZFMK-DNA-FC17941374
P0216	*Metagonia furcata*	JX023815	JX023906	JX024004	JX024112	JX023591	JX023667	ZFMK-DNA-100446397
BB14	*Metagonia G062*	AY560703	AY560673		AY560742	AY560784		
S462	*Metagonia globulosa*	MG267684	MG267976	MG268303	MG268569			ZFMK-DNA-FD04784409
S399	*Metagonia jamaica*	MG267597	MG267927	MG268216	MG268531	MG268816	MG269113	ZFMK-DNA-FD04784388
JA23	*Metagonia mariguitarensis*		DQ667781			DQ667887		ZFMK-DNA-FC17941294
JA15	*Metagonia paranapiacaba*		DQ667783			DQ667889		ZFMK-DNA-FC17941325
S400	*Metagonia potiguar*	MG267598	MG267926	MG268217	MG268532	MG268826		ZFMK-DNA-FD04784396
S401	*Metagonia rica?*	MG267599	MG267925	MG268218	MG268526	MG268883		ZFMK-DNA-FD04784404
S039	*Metagonia taruma*			MG268036	MG268368			ZFMK-DNA-FD04784079
S463	*Metagonia USA2*	MG267685	MG267977	MG268304	MG268570	MG268824	MG268988	ZFMK-DNA-FD04784417
JA85	*Metagonia Ven02*		DQ667793			DQ667900		ZFMK-DNA-FC17941403
JA8	*Metagonia Ven54*		DQ667788			DQ667894		ZFMK-DNA-FC17941364
S355	*Micromerys baiteta*	MG267573	MG267868	MG268249	MG268535	MG268630	MG269146	ZFMK-DNA-FD04784328
S296	*Micromerys raveni*	MG267550	MG267831	MG268160		MG268723	MG269144	ZFMK-DNA-FD04784251
S297	*Micromerys yidin*	MG267551	MG267832	MG268161	MG268473	MG268724	MG269145	ZFMK-DNA-FD04784252
GB37	*Micropholcus baoruco*					MG268886		
P0166	*Micropholcus baoruco*	JX023779		JX023967			JX023626	ZFMK-DNA-100446103
Mic02	*Micropholcus dalei*			KF715594	KF715612	KF715606	KF715577	ZFMK-DNA-100417581
Mic16	*Micropholcus delicatulus*		KF715619	KF715596	KF715614		KF715575	ZFMK-DNA-100417884
P0193	*Micropholcus fauroti*	JX023801	JX023887	JX023988	JX024095	JX023574	JX023647	ZFMK-DNA-100446420
P0165	*Micropholcus hispaniola*	JX023778		JX023966		JX023558	JX023625	ZFMK-DNA-100446457
S228	*Micropholcus Phi114*				MG268398		MG269112	ZFMK-DNA-FD04784214
GB38	*Micropholcus piaui*					MG268905		
Mic10	*Micropholcus piaui*		KF715615	KF715588	KF715608		KF715567	ZFMK-DNA-100436777
P0167	*Micropholcus toma?*	JX023780		JX023968	JX024078		JX023627	ZFMK-DNA-100446446
GB39	*Micropholcus ubajara*					MG268847		
Mic11	*Micropholcus ubajara*			KF715589			KF715572	ZFMK-DNA-100437029
NF69	*Modisimus berac*		FJ227995			FJ228030		ZFMK-DNA-FC17941283

code	species	12S	16S	18S	28S	CO1	H3	biobank
S356	*Modisimus cavaticus*	MG267644	MG267898	MG268195	MG268502	MG268625	MG268989	ZFMK-DNA-FD04784336
NF13	*Modisimus cienaga*		FJ227982			FJ228018		ZFMK-DNA-FC17941297
IA03	*Modisimus Cu12-11*		MG268026			MG268691		
S106	*Modisimus Cu12-11*				MG268427			ZFMK-DNA-FD04784146
IA04	*Modisimus Cu12-136*		MG268025		MG268609	MG268710		
NF3	*Modisimus cuadro*		FJ227981			FJ228017		ZFMK-DNA-FC17941353
IA01	*Modisimus culicinus*		MG268024		MG268607	MG268708		
NF43	*Modisimus culicinus*		FJ227971			FJ228007		ZFMK-DNA-FC17941314
IA05	*Modisimus elongatus*		MG268023		MG268610	MG268689		
NF101	*Modisimus enriquillo*		FJ227973			FJ228008		ZFMK-DNA-FC17941281
NF80	*Modisimus epepye*		FJ227976			FJ228011		ZFMK-DNA-FC17941331
NF66	*Modisimus fuscus*		FJ227970			FJ228006		ZFMK-DNA-FC17941354
P0228	*Modisimus G106*		JX023913	JX024016	JX024120	JX023597	JX023676	ZFMK-DNA-100446376
NF9	*Modisimus kiskeya*		FJ227978			FJ228013		ZFMK-DNA-FC17941276
NF87	*Modisimus leprete*		FJ227959			FJ227997		ZFMK-DNA-FC17941355
IA02	*Modisimus macaya*		MG268022		MG268608	MG268671		
NF37	*Modisimus makandal*		FJ227987			FJ228021		ZFMK-DNA-FC17941282
NF89	*Modisimus mango*		FJ227960			FJ227998		ZFMK-DNA-FC17941363
NF24	*Modisimus miri*		FJ227964			FJ228032		ZFMK-DNA-FC17941337
NF64	*Modisimus palvet*		FJ227962			FJ228000		ZFMK-DNA-FC17941346
NF53	*Modisimus paraiso*		FJ227974			FJ228009		ZFMK-DNA-FC17941330
S113	*Modisimus pavidus*			MG268115	MG268431	MG268709		ZFMK-DNA-FD04784091
NF71	*Modisimus roumaini*		FJ227983			FJ228019		ZFMK-DNA-FC17941291
NF81	*Modisimus seguin*		FJ227992			FJ228026		ZFMK-DNA-FC17941339
S231	*Modisimus signatus*		MG267785		MG268400	MG268624		ZFMK-DNA-FD04784238
NF68	*Modisimus tiburon*		FJ227967			FJ228003		ZFMK-DNA-FC17941275
NF1	*Modisimus toma*		FJ227980			FJ228016		ZFMK-DNA-FC17941273
NF41	*Modisimus vittatus*		FJ227991			FJ228025		ZFMK-DNA-FC17941306
OG8	*Nephila clavipes_A*		EU003276	EU003378		EU003302	EU003333	
S061	*Nerudia Mich20*				MG268365			ZFMK-DNA-FD04784152
BB16	*Ninetis subtilissima*	AY560705			AY560744			
P0234	*Nita elsaff*	JX023823	JX023917	JX024021		JX023601	JX023681	ZFMK-DNA-100446382
P0144	*Nyikoa limbe*		JX023851	JX023952	JX024065	JX023544		ZFMK-DNA-100446469
P0179	*Nyikoa limbe*	JX023789	JX023879	JX023976	JX024085	JX023564	JX023638	ZFMK-DNA-100446423
OG2	*Oecobius sp*		FJ607466	FJ607505	FJ607540	FJ607579	FJ607617	
S320	*Otavaloa cf angotero*		MG267853	MG268176			MG268994	ZFMK-DNA-FD04784341
S465	*Otavaloa lisei*	MG267686	MG267979	MG268306	MG268572	MG268631	MG268992	ZFMK-DNA-FD04784433
S021	*Otavaloa piro?*	MG267434	MG267759	MG268041		MG268822	MG268993	ZFMK-DNA-FD04784133
S359	*Panjange alba*	MG267574	MG267869		MG268543	MG268818		ZFMK-DNA-FD04784265
S466	*Panjange camiguin*	MG267687	MG267980	MG268307	MG268573	MG268908		ZFMK-DNA-FD04784441
S066	*Panjange casaroro*		MG267760		MG268385	MG268899		ZFMK-DNA-FD04784089
S360	*Panjange cavicola*	MG267575	MG267870		MG268544	MG268878		ZFMK-DNA-FD04784273
S218	*Panjange dinagat*	MG267479			MG268390	MG268901		ZFMK-DNA-FD04784237
GB40	*Panjange iban*					MG268929		
S169	*Panjange iban*	MG267504			MG268410			ZFMK-DNA-FD04784220
S361	*Panjange Ind103*	MG267576	MG267872	MG268251	MG268536	MG268909	MG269149	ZFMK-DNA-FD04784281
S362	*Panjange Ind109*	MG267577	MG267871	MG268253	MG268490	MG268958		ZFMK-DNA-FD04784289
S173	*Panjange kapit*	MG267491	MG267935		MG268415	MG268931		ZFMK-DNA-FD04784157
GB41	*Panjange kubah*					MG268932		
S171	*Panjange kubah*	MG267506			MG268412			ZFMK-DNA-FD04784236
S010	*Panjange lanthana*	MG267455		MG268107		MG268881		ZFMK-DNA-FD04784140
S224	*Panjange malagos*	MG267475			MG268395	MG268898		ZFMK-DNA-FD04784182
S222	*Panjange marilog*	MG267477			MG268393	MG268900		ZFMK-DNA-FD04784166
S172	*Panjange niah?*	MG267507						ZFMK-DNA-FD04784244
S174	*Panjange pueh*	MG267492			MG268416	MG268930		ZFMK-DNA-FD04784165
GB42	*Panjange seowi*					MG268933		
S170	*Panjange seowi*	MG267505			MG268411			ZFMK-DNA-FD04784228
S011	*Papiamenta MRAC639*	MG267439		MG268037				ZFMK-DNA-FD04784148
P0245	*Paramicromerys betsileo?*	JX023828	JX023923					ZFMK-DNA-100433806
P0244	*Paramicromerys CAS11*		JX023922	JX024030				ZFMK-DNA-100433815
S363	*Paramicromerys CAS17*	MG267611	MG267899	MG268235		MG268638		ZFMK-DNA-FD04784297
P0238	*Paramicromerys CAS3*		JX023920	JX024025				ZFMK-DNA-100437925
P0221	*Paramicromerys G046*			JX024009	JX024115	JX023594		ZFMK-DNA-100446392
S383	*Paramicromerys? CAS13*	MG267613	MG267900	MG268237		MG268951	MG269092	ZFMK-DNA-FD04784267
S467	*Paramicromerys? CAS4*	MG267688	MG267981	MG268308	MG268596	MG268940	MG269091	ZFMK-DNA-FD04784354
P0151	*Pehrforsskalia conopyga*	JX023764	JX023857	JX023956	JX024069	JX023549	JX023616	ZFMK-DNA-100446462
P0168	*Pehrforsskalia conopyga*	JX023781	JX023870	JX023969		JX023559	JX023628	ZFMK-DNA-100446445
JA45	*Pholcophora americana*		DQ667797		DQ667848	DQ667905		ZFMK-DNA-FC17941280
JA46	*Pholcophora americana*		DQ667798			DQ667906		ZFMK-DNA-FC17941288
S298	*Pholcophora? Car544*	MG267552	MG267833		MG268475	MG268741		ZFMK-DNA-FD04784260

code	species	12S	16S	18S	28S	CO1	H3	biobank
S256	*'Pholcus gracillimus' sensu Huber 2011*	MG267563	MG267857	MG268133		MG268858	MG269180	ZFMK-DNA-FD04784224
S257	*'Pholcus kohi' sensu Huber 2011*	MG267521	MG267858	MG268134		MG268859		ZFMK-DNA-FD04784232
S321	*Pholcus agadir*		MG267854	MG268177	MG268484	MG268754	MG268987	ZFMK-DNA-FD04784254
S178	*Pholcus andulau*					MG268927	MG269175	ZFMK-DNA-FD04784173
S469	*Pholcus andulau*	MG267690				MG268928		ZFMK-DNA-FD04784370
S071	*Pholcus arayat*		MG267761	MG268260				ZFMK-DNA-FD04784129
P0281	*Pholcus atrigularis*	JX023846	JX023945			JX023606		ZFMK-DNA-100437887
P0177	*Pholcus attuleh*	JX023788	JX023877	JX023974		JX023563	JX023637	ZFMK-DNA-100446436
S070	*Pholcus baguio*		MG267769	MG268259		MG268865	MG269174	ZFMK-DNA-FD04784121
P0196	*Pholcus baka*	JX023803	JX023890	JX023989		JX023575	JX023649	ZFMK-DNA-100446417
GB43	*Pholcus bamboutos*					MG268848		
P0181	*Pholcus bamboutos*	JX023791	JX023881				JX023640	ZFMK-DNA-100446425
P0195	*Pholcus bamboutos*	JX023802	JX023889					ZFMK-DNA-100446418
S470	*Pholcus bario*	MG267691	MG267983	MG268310	MG268575	MG268855		ZFMK-DNA-FD04784378
S366	*Pholcus barisan*	MG267581	MG267873	MG268252	MG268537	MG268959	MG269139	ZFMK-DNA-FD04784321
P0250	*Pholcus beijingensis*	JX023831	JX023925	JX024034				ZFMK-DNA-100437904
S064	*Pholcus bicornutus*	MG267444	MG267762	MG268033	MG268388			ZFMK-DNA-FD04784073
S012	*Pholcus bikilai*	MG267441	MG267763	MG268032			MG269173	ZFMK-DNA-FD04784061
P0147	*Pholcus bourgini*	JX023760	JX023854			JX023546	JX023612	ZFMK-DNA-100446466
S266	*Pholcus buatong*	MG267530					MG269171	ZFMK-DNA-FD04784209
S271	*Pholcus bukittimah*	MG267535	MG267810	MG268141	MG268455	MG268916		ZFMK-DNA-FD04784249
S364	*Pholcus camba*	MG267578	MG267876	MG268257		MG268860	MG269178	ZFMK-DNA-FD04784305
P0247	*Pholcus cf jaegeri*	JX023829	JX023924	JX024032	JX024129	JX023603	JX023685	ZFMK-DNA-100437916
P0162	*Pholcus chappuisi*	JX023775	JX023867	JX023964			JX023623	ZFMK-DNA-100446454
P0201	*Pholcus chappuisi*	JX023804	JX023894	JX023994	JX024102		JX023652	ZFMK-DNA-100446412
P0148	*Pholcus chattoni*	JX023761					JX023613	ZFMK-DNA-100446465
S101	*Pholcus chengpoi*				MG268424	MG268907		ZFMK-DNA-FD04784106
S482	*Pholcus cibodas*	MG267701		MG268320	MG268580	MG268950		ZFMK-DNA-FD04784379
S324	*Pholcus creticus*	MG267562	MG267856	MG268179				ZFMK-DNA-FD04784278
P0182	*Pholcus crypticolens*	JX023792	JX023882	JX023977		JX023566		ZFMK-DNA-100446426
P0222	*Pholcus dade*	JX023817	JX023909	JX024010	JX024116	JX023595		ZFMK-DNA-100446391
P0172	*Pholcus debilis*	JX023784	JX023873				JX023632	ZFMK-DNA-100446441
P0225	*Pholcus dentatus*	JX023819	JX023911	JX024013			JX023674	ZFMK-DNA-100446388
S258	*Pholcus diopsis*	MG267522			MG268444	MG268879		ZFMK-DNA-FD04784240
GB13	*Pholcus domingo*					MG268947		
S013	*Pholcus domingo*	MG267440						ZFMK-DNA-FD04784069
P0149	*Pholcus doucki*	JX023762	JX023855			JX023547	JX023614	ZFMK-DNA-100446464
S259	*Pholcus erawan*	MG267523	MG267801		MG268445	MG268839	MG269151	ZFMK-DNA-FD04784248
S260	*Pholcus erawan*	MG267524	MG267802		MG268446		MG269152	ZFMK-DNA-FD04784161
S024	*Pholcus ethagala*	MG267448	MG267741				MG268974	ZFMK-DNA-FD04784149
SB017	*Pholcus ethagala?*	MG267733					MG268977	
P0158	*Pholcus fagei*	JX023771	JX023864	JX023961	JX024074		JX023621	ZFMK-DNA-100446450
P0159	*Pholcus fagei*	JX023772	JX023865		JX024075	JX023553		ZFMK-DNA-100446451
P0287	*Pholcus fengcheng*	JX023848		JX024062	JX024145	JX023608	JX023713	ZFMK-DNA-100433811
S105	*Pholcus fragillimus*	MG267486		MG268111	MG268426	KJ650076		ZFMK-DNA-FD04784138
S047	*Pholcus G044*			MG268027	MG268352	DQ667927		ZFMK-DNA-FD04784143
S261	*Pholcus gombak*	MG267525	MG267803		MG268447	MG268915		ZFMK-DNA-FD04784169
P0202	*Pholcus Gui32*	JX023805		JX023995		JX023579	JX023653	ZFMK-DNA-100446110
S262	*Pholcus halabala*	MG267526	MG267804		MG268448	MG268843	MG269159	ZFMK-DNA-FD04784177
S365	*Pholcus hurau*	MG267579	MG267874			MG268838		ZFMK-DNA-FD04784313
S299	*Pholcus jinwum*	MG267553		MG268162	MG268476	MG268925	MG269177	ZFMK-DNA-FD04784268
P0288	*Pholcus jiuwei*	JX023849	JX023950	JX024063	JX024146	JX023609	JX023714	ZFMK-DNA-100437875
P0145	*Pholcus kakum*	JX023759	JX023852	JX023953	JX024066		JX023610	ZFMK-DNA-100446468
GB44	*Pholcus kawit*					MG268866		
S225	*Pholcus kawit*	MG267474	MG267799					ZFMK-DNA-FD04784190
S367	*Pholcus kerinci*	MG267582				MG268949		ZFMK-DNA-FD04784329
S268	*Pholcus khaolek*	MG267532	MG267808	MG268138		MG268832	MG269155	ZFMK-DNA-FD04784225
P0248	*Pholcus kimi*	JX023830		JX024033	JX024130			ZFMK-DNA-100433839
P0203	*Pholcus kindia*	JX023806	JX023895			JX023580	JX023654	ZFMK-DNA-100446399
S186	*Pholcus kipungit*					MG268835		ZFMK-DNA-FD04784213
S502	*Pholcus kottawagamaensis*	MG267721	MG268009			MG268888		ZFMK-DNA-FD05101791
S267	*Pholcus krabi*	MG267531	MG267807	MG268137	MG268452	MG268836		ZFMK-DNA-FD04784217
P0171	*Pholcus kribi*	JX023783	JX023872			JX023561	JX023631	ZFMK-DNA-100446442
S265	*Pholcus kuhapimuk*	MG267529	MG267806	MG268136	MG268451	MG268830	MG269156	ZFMK-DNA-FD04784201
S183	*Pholcus lambir*					MG268926	MG269176	ZFMK-DNA-FD04784189
S263	*Pholcus ledang*	MG267527	MG267805		MG268449	MG268917		ZFMK-DNA-FD04784185
P0163	*Pholcus leruthi*	JX023776	JX023868			JX023556		ZFMK-DNA-100446455
S185	*Pholcus lintang*			MG268118	MG268418	MG268842	MG269158	ZFMK-DNA-FD04784205
JA42	*Pholcus manueli*		DQ667800			DQ667908		ZFMK-DNA-FC17941351
BB19	*Pholcus manueli (sp1 Bruvo)*	AY560706			AY560749	AY560786		
S056	*Pholcus maronita?*	MG267467	MG267765	MG268029		MG268880		ZFMK-DNA-FD04784112

code	species	12S	16S	18S	28S	CO1	H3	biobank
S503	*Pholcus maturata*	MG267727	MG268010			MG268887		ZFMK-DNA-FD05101704
S226	*Pholcus matutum*	MG267473			MG268396	MG268948		ZFMK-DNA-FD04784198
S368	*Pholcus mentawir*	MG267583	MG267877	MG268258	MG268538	MG268711	MG269135	ZFMK-DNA-FD04784337
S053	*Pholcus moca*		MG267766	MG268030	MG268350			ZFMK-DNA-FD04784088
P0230	*Pholcus MRAC553*		JX023914	JX024017	JX024121	JX023598	JX023677	ZFMK-DNA-100446378
S188	*Pholcus mulu*	MG267493		MG268119	MG268419	MG268863		ZFMK-DNA-FD04784221
S264	*Pholcus narathiwat*	MG267528		MG268135	MG268450	MG268834		ZFMK-DNA-FD04784193
S273	*Pholcus negara*	MG267537	MG267812	MG268142		MG268861	MG269179	ZFMK-DNA-FD04784170
GB45	*Pholcus nkoetye*					MG268819		
P0270	*Pholcus nkoetye*	JX023840	JX023937				JX023701	ZFMK-DNA-100437893
GB46	*Pholcus olangapo*					MG268857		
S014	*Pholcus olangapo*	MG267443	MG267797	MG268028	MG268414			ZFMK-DNA-FD04784077
BB17	*Pholcus opilionoides*	AY560707	AY560674					
P0232	*Pholcus ornatus*	JX023822	JX023916	JX024019	JX024122	JX023599	JX023679	ZFMK-DNA-100446380
S065	*Pholcus pagbilao*	MG267442	MG267767					ZFMK-DNA-FD04784081
S072	*Pholcus pagbilao*	MG267496	MG267798	MG268108		MG268862		ZFMK-DNA-FD04784137
BB18a	*Pholcus phalangioides*	AY560708	AY560675					
JA43	*Pholcus phalangioides*		DQ667809			DQ667920		ZFMK-DNA-FC17941359
P0286	*Pholcus phoenixus*	JX023847	JX023949	JX024061	JX024144		JX023712	ZFMK-DNA-100437877
S274	*Pholcus phui*	MG267538	MG267813		MG268456	MG268912		ZFMK-DNA-FD04784178
S236	*Pholcus pingtung*	MG267469				MG268906	MG269131	ZFMK-DNA-FD04784183
S051	*Pholcus punu*		MG267764	MG268031	MG268351			ZFMK-DNA-FD04784072
S369	*Pholcus quinquenotatus*	MG267584	MG267878	MG268250		MG268828	MG269150	ZFMK-DNA-FD04784345
S052	*Pholcus rawiriae*	MG267445	MG267768				MG269134	ZFMK-DNA-FD04784080
S182	*Pholcus sabah*			MG268117	MG268417	MG268841	MG269157	ZFMK-DNA-FD04784181
S275	*Pholcus satun*		MG267814			MG268911		ZFMK-DNA-FD04784186
GB47	*Pholcus schwendingeri*					MG268910		
S276	*Pholcus schwendingeri*	MG267539	MG267815		MG268457		MG269170	ZFMK-DNA-FD04784194
S370	*Pholcus sepaku*	MG267625	MG267916	MG268242	MG268521	MG268844	MG269160	ZFMK-DNA-FD04784258
S371	*Pholcus singalang*	MG267580	MG267875			MG268837		ZFMK-DNA-FD04784266
S234	*Pholcus SMF422*	MG267470			MG268402	MG268869	MG269132	ZFMK-DNA-FD04784167
S104	*Pholcus spilis*	MG267485		MG268110		MG268885	MG269172	ZFMK-DNA-FD04784130
GB48	*Pholcus sudhami*					MG268831		
S277	*Pholcus sudhami*	MG267540	MG267816	MG268143			MG269154	ZFMK-DNA-FD04784202
S471	*Pholcus taarab*	MG267692	MG267984	MG268311		MG268827	MG269161	ZFMK-DNA-FD04784386
P0155	*Pholcus taita*	JX023768	JX023861	JX023958			JX023620	ZFMK-DNA-100446447
S472	*Pholcus tambunan*	MG267693	MG267985		MG268576	MG268856		ZFMK-DNA-FD04784394
S272	*Pholcus tanahrata*	MG267536	MG267811			MG268913		ZFMK-DNA-FD04784162
P0258	*Pholcus twa*	JX023833	JX023928	JX024039			JX023689	ZFMK-DNA-100437912
S269	*Pholcus ubin*	MG267533	MG267809	MG268139	MG268453	MG268840	MG269153	ZFMK-DNA-FD04784233
GB49	*Pholcus uludong*					MG268914		
S270	*Pholcus uludong*	MG267534		MG268140	MG268454			ZFMK-DNA-FD04784241
S473	*Pholcus wahehe*	MG267694	MG267986			MG268864	MG269166	ZFMK-DNA-FD04784402
JA71	*Physocyclus dugesi*	MG267437		MG268106	MG268383	DQ667928		ZFMK-DNA05-JA71
S474	*Physocyclus enaulus*	MG267695		MG268312		MG268722		ZFMK-DNA-FD04784410
JA70	*Physocyclus franckei*	MG267482	DQ667825	MG268105	MG268423	DQ667930		ZFMK-DNA05-JA70
BB21	*Physocyclus globosus*	AY560712			AY560751	AY560788		
OG29	*Pimoa breuili*		JN010167	JN010185	JN010190			
OG32	*Pimoa sp*		FJ607471	FJ607510	FJ607545	FJ607584	FJ607622	
S475	*Pisaboa silvae*	MG267696	MG267987	MG268313	MG268577			ZFMK-DNA-FD04784418
S372	*Platnicknia Cu12-100*		MG267904	MG268227	MG268509	MG268702	MG269020	ZFMK-DNA-FD04784274
S115	*Platnicknia Cu12-2*			MG268116		MG268703		ZFMK-DNA-FD04784107
S373	*Platnicknia Cu12-61*	MG267590	MG267905	MG268226	MG268510	MG268704	MG269021	ZFMK-DNA-FD04784282
S374	*Platnicknia Cu12-99*		MG267906	MG268228		MG268699	MG269018	ZFMK-DNA-FD04784290
IA07	*Platnicknia incerta*		MG268021			MG268701		
S114	*Platnicknia incerta*					MG268700	MG269019	ZFMK-DNA-FD04784099
OG15	*Plectreurys tristis*		FJ607472	FJ607511	FJ607546	FJ607585	FJ607623	
JA80	*Priscula Astr07*		DQ667838			DQ667853		ZFMK-DNA-FC17941458
JA114	*Priscula binghamae*		DQ667827			DQ667932		ZFMK-DNA-FC17941445
JA22	*Priscula Ven02/80-85*		DQ667828		DQ667850	DQ667933		ZFMK-DNA-FC17941286
S307	*Priscula Ven02/80-85*		MG267841		MG268533		MG268972	ZFMK-DNA-FD04784332
S476	*Priscula venezuelana*	MG267697	MG267988	MG268314		MG268867		ZFMK-DNA-FD04784426
S477	*Psilochorus concolor*			MG268315	MG268578			ZFMK-DNA-FD04784434
S046	*Psilochorus imitatus*	MG267426	MG267771	MG268040				ZFMK-DNA-FD04784135
BB24	*Psilochorus imitatus=sp.1 Bruvo*	AY560715			AY560754	AY560790		
S478	*Psilochorus pallidulus*	MG267715	MG267989	MG268316		MG268628		ZFMK-DNA-FD04784442
P0210	*Psilochorus SA Br10-36*	JX023810	JX023901	JX024001	JX024109	JX023586	JX023661	ZFMK-DNA-100446406
S032	*Psilochorus SA Br11-40*		MG267770	MG268065	MG268367	MG268706		ZFMK-DNA-FD04784118
BB12a	*Psilochorus SA itaguyrussu*	AY560701	AY560672		AY560740	AY560782		
P0219	*Psilochorus SA itaguyrussu*			JX024007	JX024113	JX023593	JX023670	ZFMK-DNA-100446394
BB23	*Psilochorus simoni*	AY560714			AY560753	AY560789		
BB25	*Quamtana bonamanzi*	AY560719			AY560757			

code	species	12S	16S	18S	28S	CO1	H3	biobank
GB50	*Quamtana bonamanzi*					MG268745		
P0183	*Quamtana Cam117*	JX023793	JX023883	JX023978	JX024086	JX023567	JX023641	ZFMK-DNA-100446427
S291	*Quamtana CAS5*	MG267547	MG267827	MG268156	MG268470	MG268806	MG269148	ZFMK-DNA-FD04784219
P0241	*Quamtana CAS7*	JX023825		JX024028	JX024127		JX023683	ZFMK-DNA-100433857
BB26	*Quamtana embuleni*	AY560721			AY560759	AY560793		
P0285	*Quamtana filmeri*		JX023948			JX023607	JX023711	ZFMK-DNA-100437878
P0262	*Quamtana kabale*			JX024042			JX023693	ZFMK-DNA-100437901
S063	*Quamtana kitahurira*			MG268075			MG269138	ZFMK-DNA-FD04784065
P0204	*Quamtana nyahururu*		JX023896	JX023996	JX024104	JX023581	JX023655	ZFMK-DNA-100446400
P0243	*Quamtana nylsvley?*	JX023827		JX024029	JX024128		JX023684	ZFMK-DNA-100433809
P0170	*Quamtana oku*	JX023782	JX023871	JX023971	JX024080		JX023630	ZFMK-DNA-100446443
P0284	*Quamtana umzinto*			JX024060			JX023710	ZFMK-DNA-100433855
BB27	*Quamtana vidal*	AY560720			AY560758	AY560792		
OG3	*Rhode scutiventris*		EU139684		EU139822	EU139636	EU139754	
S279	*Savarna kaeo*		MG267818	MG268145	MG268459			ZFMK-DNA-FD04784218
S278	*Savarna kraburiensis*		MG267817	MG268144	MG268458	MG268939		ZFMK-DNA-FD04784210
S306	*Savarna miser*		MG267840	MG268168	MG268479	MG268942		ZFMK-DNA-FD04784324
S280	*Savarna tessellata*			MG268146	MG268460	MG268760		ZFMK-DNA-FD04784226
S281	*Savarna thaleban*		MG267819	MG268147	MG268461	MG268941		ZFMK-DNA-FD04784234
OG25	*Segestria bavarica*	AY560731			AY560765	AY560802		
OG21	*Sicarius terrosus*		EU817810		EU817740	EU817704		
S504	*Sihala ceylonica*	MG267722	MG268011	MG268339	MG268602	MG268845	MG269168	ZFMK-DNA-FD05101712
S505	*Sihala SL43*	MG267723	MG268012	MG268340		MG268829	MG269167	ZFMK-DNA-FD05101720
S506	*Sihala SL63*	MG267724	MG268013	MG268341	MG268603	MG268846	MG269169	ZFMK-DNA-FD05101728
GB51	*Smeringopina ankasa*					MG268634		
S054	*Smeringopina ankasa*		MG267772	MG268081				ZFMK-DNA-FD04784096
P0173	*Smeringopina attuleh*		JX023874				JX023633	ZFMK-DNA-100446440
S491	*Smeringopina bamenda*	MG267710	MG267997				MG269120	ZFMK-DNA-FD05101703
GB14	*Smeringopina bayaka*					MG268662		
GB15	*Smeringopina belinga*					MG268665		
P0152	*Smeringopina bineti*	JX023765	JX023858				JX023617	ZFMK-DNA-100446461
S300	*Smeringopina bomfobiri*	MG267554	MG267834	MG268163		MG268633	MG269118	ZFMK-DNA-FD04784276
S492	*Smeringopina bwiti*	MG267711	MG267998			MG268659	MG269068	ZFMK-DNA-FD05101711
S042	*Smeringopina camerunensis*		MG267773	MG268079				ZFMK-DNA-FD04784103
GB16	*Smeringopina chaillu*					MG268663		
S404	*Smeringopina djidji*	MG267602	MG267930	MG268220		MG268666	MG269069	ZFMK-DNA-FD04784428
GB52	*Smeringopina ebolowa*					MG268668		
P0175	*Smeringopina ebolowa*	JX023786	JX023876				JX023635	ZFMK-DNA-100446438
GB17	*Smeringopina essotah*					MG268664		
GB18	*Smeringopina fang*					MG268654		
P0153	*Smeringopina guineensis*	JX023766	JX023859			JX023550	JX023618	ZFMK-DNA-100446114
GB19	*Smeringopina iboga*					MG268645		
S041	*Smeringopina kala*	MG267459	MG267774	MG268078	MG268387	MG268622	MG269119	ZFMK-DNA-FD04784095
S402	*Smeringopina kinguele*	MG267600	MG267928	MG268219	MG268527		MG269181	ZFMK-DNA-FD04784412
S044	*Smeringopina kribi*	MG267460	MG267775	MG268080		MG268660		ZFMK-DNA-FD04784119
GB20	*Smeringopina lekoni*					MG268652		
S043	*Smeringopina mbouda*		MG267776	MG268082			MG269066	ZFMK-DNA-FD04784111
S403	*Smeringopina mohoba*	MG267601	MG267929			MG268655	MG269182	ZFMK-DNA-FD04784420
S049	*Smeringopina moudouma*	MG267462	MG267777	MG268077	MG268386			ZFMK-DNA-FD04784151
GB21	*Smeringopina ndjole*					MG268644		
P0180	*Smeringopina nyasoso*	JX023790	JX023880			JX023565	JX023639	ZFMK-DNA-100446424
S405	*Smeringopina ogooue*	MG267603	MG267931			MG268669	MG269070	ZFMK-DNA-FD04784436
GB22	*Smeringopina sahoue*					MG268658		
S493	*Smeringopina simintang*	MG267716	MG267999	MG268329		MG268667		ZFMK-DNA-FD05101719
S301	*Smeringopina tchimbele*		MG267835	MG268164		MG268661	MG269067	ZFMK-DNA-FD04784284
GB54	*Smeringopina tebe*					MG268653		
S050	*Smeringopina tebe*	MG267461	MG267778	MG268076				ZFMK-DNA-FD04784064
S017	*Smeringopus arambourgi*	MG267458	MG267779	MG268039	MG268382		MG269061	ZFMK-DNA-FD04784101
P0224	*Smeringopus atomarius?*		JX023910	JX024012	JX024117		JX023673	ZFMK-DNA-100446389
P0266	*Smeringopus bujongolo*	JX023837	JX023933	JX024045			JX023697	ZFMK-DNA-100437897
P0161	*Smeringopus chogoria*	JX023774	JX023866	JX023963				ZFMK-DNA-100446453
P0143	*Smeringopus cylindrogaster*		JX023850	JX023951	JX024064			ZFMK-DNA-100446470
P0178	*Smeringopus cylindrogaster*		JX023878	JX023975	JX024084			ZFMK-DNA-100446102
GB24	*Smeringopus isangi*					MG268870		
S407	*Smeringopus lesserti*	MG267605	MG267933	MG268222	MG268530	MG268871	MG269062	ZFMK-DNA-FD04784357
S408	*Smeringopus lotzi*	MG267606	MG267934	MG268223	MG268529	MG268872	MG269063	ZFMK-DNA-FD04784365
P0257	*Smeringopus mgahinga*		JX023927	JX024038			JX023688	ZFMK-DNA-100437911

code	species	12S	16S	18S	28S	CO1	H3	biobank
P0265	*Smeringopus mpanga*		JX023932				JX023696	ZFMK-DNA-100433856
BB28	*Smeringopus natalensis*	AY560717			AY560755			
P0157	*Smeringopus ngangao*	JX023770	JX023863	JX023960	JX024073			ZFMK-DNA-100446449
P0264	*Smeringopus pallidus*	JX023836	JX023931	JX024044	JX024136		JX023695	ZFMK-DNA-100437899
S406	*Smeringopus peregrinoides*	MG267604	MG267932	MG268221	MG268528		MG269060	ZFMK-DNA-FD04784349
P0227	*Smeringopus similis?*	JX023820	JX023912	JX024015	JX024119		JX023675	ZFMK-DNA-100446129
GB55	*Spermophora akwamu*					MG268747		
S018	*Spermophora akwamu*	MG267457		MG268042			MG269055	ZFMK-DNA-FD04784109
P0187	*Spermophora awalai*	JX023797		JX023982	JX024089	JX023570	JX023644	ZFMK-DNA-100446431
GB56	*Spermophora bukusu*					MG268678		
P0273	*Spermophora bukusu*	JX023842	JX023939	JX024049				ZFMK-DNA-100437879
S376	*Spermophora cf akwamu*	MG267614	MG267902	MG268236	MG268507	MG268746	MG269056	ZFMK-DNA-FD04784306
GB27	*Spermophora dieke*					MG268748		
S055	*Spermophora dieke*	MG267456	MG267780	MG268043				ZFMK-DNA-FD04784104
S019	*Spermophora estebani*	MG267454		MG268072	MG268369		MG269058	ZFMK-DNA-FD04784117
S375	*Spermophora Ind27*	MG267624	MG267903	MG268241	MG268519	MG268743	MG269185	ZFMK-DNA-FD04784298
S335	*Spermophora Ind90*	MG267634	MG267882	MG268212	MG268493	MG268891	MG269057	ZFMK-DNA-FD04784263
P0189	*Spermophora kirinyaga*	JX023798		JX023984	JX024091			ZFMK-DNA-100446433
GB57	*Spermophora kyambura*					MG268877		
P0174	*Spermophora kyambura*	JX023785	JX023875	JX023972			JX023634	ZFMK-DNA-100446439
P0263	*Spermophora kyambura*	JX023835	JX023930	JX024043			JX023694	ZFMK-DNA-100437900
P0184	*Spermophora maathaiae*	JX023794	JX023884	JX023979	JX024087	JX023568		ZFMK-DNA-100446428
S282	*Spermophora Mal2*			MG268148	MG268462	MG268889	MG269184	ZFMK-DNA-FD04784242
GB58	*Spermophora mau*					MG268679		
P0275	*Spermophora mau*	JX023844		JX024051	JX024140		JX023703	ZFMK-DNA-100437881
P0164	*Spermophora minotaura*	JX023777	JX023869	JX023965	JX024077	JX023557	JX023624	ZFMK-DNA-100446456
P0186	*Spermophora minotaura*	JX023796		JX023981	JX024088	JX023569	JX023643	ZFMK-DNA-100446430
P0272	*Spermophora minotaura*		JX023938	JX024048	JX024139	JX023604	JX023702	ZFMK-DNA-100433830
P0278	*Spermophora minotaura*		JX023943	JX024054	JX024143		JX023706	ZFMK-DNA-100437884
P0226	*Spermophora MRAC525*			JX024014	JX024118			ZFMK-DNA-100446118
S020	*Spermophora Phi109*	MG267453		MG268071			MG269059	ZFMK-DNA-FD04784125
BB29	*Spermophora senoculata*	AY560718			AY560756	AY560791		
S311	*Spermophora senoculata*	MG267557	MG267844		MG268483	MG268642		ZFMK-DNA-FD04784269
S479	*Spermophora tonkoui*	MG267698	MG267990	MG268317		MG268882	MG269121	ZFMK-DNA-FD04784355
GB59	*Spermophora ziama*					MG268792		
P0185	*Spermophora ziama*	JX023795		JX023980			JX023642	ZFMK-DNA-100446429
P0256	*Spermophorides cuneata*			JX024037	JX024133			ZFMK-DNA-100437910
NA001	*Spermophorides fuerteventurensis*				MG268611	MG268903		
NA002	*Spermophorides mercedes*				MG268612	MG268902		
S308	*Stenosfemuraia cuadrata*		MG267842	MG268169	MG268480	MG268707		ZFMK-DNA-FD04784340
S108	*Stygopholcus absoloni?*	MG267487		MG268112	MG268428	MG268938		ZFMK-DNA-FD04784154
S109	*Stygopholcus absoloni?*	MG267488				MG268936		ZFMK-DNA-FD04784067
S112	*Stygopholcus absoloni?*	MG267490		MG268114	MG268430	MG268937		ZFMK-DNA-FD04784083
S110	*Stygopholcus Bal3*	MG267489		MG268113	MG268429	MG268935		ZFMK-DNA-FD04784075
S317	*Stygopholcus photophilus*	MG267561	MG267850	MG268173		MG268934	MG269114	ZFMK-DNA-FD04784317
BB31	*Systenita prasina*	AY560722			AY560761			
JA88	*Systenita prasina*					DQ667939		ZFMK-DNA-FC17941427
JA131	*Tainonia samana*		FJ799766			FJ799781		ZFMK-DNA-FC17941391
S480	*Tainonia serripes*	MG267699	MG268003	MG268318		MG268670	MG269127	ZFMK-DNA-FD04784363
JA134	*Tainonia visite*		FJ799769			FJ799782		ZFMK-DNA-FC17941415
OG31	*Tetragnatha versicolor_A*	EU003246			EU003429	EU003308		
S302	*Trichocyclus balladong*	MG267555	MG267836	MG268165		MG268635		ZFMK-DNA-FD04784292
BB32	*Trichocyclus cf balladong*	AY560686	AY560664		AY560733	AY560772		
S303	*Trichocyclus cf warianga*	MG267556	MG267837	MG268166	MG268477			ZFMK-DNA-FD04784300
S384	*Tupigea ale*	MG267591	MG267909			MG268720	MG269190	ZFMK-DNA-FD04784275
JA48	*Tupigea angelim*		DQ667834			DQ667941		ZFMK-DNA-FC17941304
S481	*Tupigea angelim*	MG267700		MG268319	MG268579		MG269187	ZFMK-DNA-FD04784371
S494	*Tupigea Br10-32*	MG267712	MG268000	MG268330		MG268759		ZFMK-DNA-FD05101727
S411	*Tupigea Br10-40*	MG267609	MG267923			MG268721	MG269191	ZFMK-DNA-FD04784389
S409	*Tupigea Br14-47*	MG267607		MG268224		MG268719	MG269189	ZFMK-DNA-FD04784373
JA13	*Tupigea cantareira*		DQ667835			DQ667942		ZFMK-DNA-FC17941309
P0218	*Tupigea maza*		JX023907	JX024006			JX023669	ZFMK-DNA-100446395
P0214	*Tupigea nadleri*		JX023904	JX024002	JX024110	JX023589	JX023665	ZFMK-DNA-100446410
GB30	*Tupigea paula*					MG268716		
S377	*Tupigea penedo*	MG267615		MG268243	MG268518	MG268717		ZFMK-DNA-FD04784314

code	species	12S	16S	18S	28S	CO1	H3	biobank
S385	*Tupigea teresopolis?*	MG267616	MG267910			MG268718	MG269188	ZFMK-DNA-FD04784283
OG12	*Uloborus glomosus*	EU003247		EU003366	EU003438	EU003310	EU003340	
OG13	*Uroctea durandi*			FJ948939	FJ948980	FJ949021	FJ949058	
S283	*Uthina huifengi?*	MG267541	MG267820	MG268149	MG268463	MG268962		ZFMK-DNA-FD04784250
S378	*Uthina Ind119*	MG267585		MG268254	MG268539	MG268961		ZFMK-DNA-FD04784322
S379	*Uthina Ind121*	MG267586		MG268255	MG268540	MG268957		ZFMK-DNA-FD04784330
S380	*Uthina Ind67*	MG267587		MG268256	MG268541	MG268960		ZFMK-DNA-FD04784338
S284	*Uthina khaosokensis*		MG267821	MG268150	MG268464	MG268966		ZFMK-DNA-FD04784163
S102	*Uthina luzonica*	MG267483			MG268425	MG268964		ZFMK-DNA-FD04784114
S217	*Uthina luzonica*			MG268120	MG268420	MG268963		ZFMK-DNA-FD04784229
S285	*Uthina ratchaburi*	MG267542	MG267822	MG268151	MG268465	MG268965		ZFMK-DNA-FD04784171
SB052	*Wanniyala agrabopath*	MG267731		MG268348		MG268946		
S022	*Wanniyala SL71*		MG267781	MG268053				ZFMK-DNA-FD04784141
SB063	*Wanniyala SL61*	MG267732		MG268349			MG269088	
S507	*Wanniyala SL38*	MG267725	MG268014	MG268342	MG268604	MG268944	MG269089	ZFMK-DNA-FD05101736
S508	*Wanniyala SL55*		MG268015	MG268343	MG268605	MG268943	MG269090	ZFMK-DNA-FD05101744
S509	*Wanniyala SL61*	MG267726	MG268016	MG268344	MG268606	MG268945		ZFMK-DNA-FD05101752
S304	*Waunana modesta?*		MG267838		MG268478		MG269013	ZFMK-DNA-FD04784308
S293	*Wugigarra kalamai*	MG267548	MG267828	MG268157				ZFMK-DNA-FD04784227
S233	*Wugigarra QMB5*			MG268093			MG268971	ZFMK-DNA-FD04784159
P0251	*Wugigarra undanbi*		JX023926	JX024035	JX024131		JX023686	ZFMK-DNA-100433838
S232	*Wugigarra yawai*	MG267471	MG267784	MG268092	MG268401	MG268749	MG268973	ZFMK-DNA-FD04784246
S381	*Wugigarra? Ind107*	MG267568		MG268192		MG268613	MG269053	ZFMK-DNA-FD04784346
S495	*Wugigarra? Ind117*	MG267713	MG268001	MG268331		MG268751		ZFMK-DNA-FD05101735
S382	*Wugigarra? Ind82*	MG267569	MG267880	MG268247		MG268750	MG269054	ZFMK-DNA-FD04784259
S496	*Wugigarra? Ind96*	MG267714	MG268002	MG268332		MG268740		ZFMK-DNA-FD05101743
P0240	*Zatavua analalava?*	JX023824	JX023921	JX024027	JX024126			ZFMK-DNA-100433837
S045	*Zatavua CAS8*		MG267782	MG268034	MG268366			ZFMK-DNA-FD04784127
OG33	*Zygiella x-notata*	EU003248	EU003251	EU003367	EU003431	EU003311	EU003341	

Table S2: Sampling localities along with decimal coordinates (WGS84) and the biome in which the majority of the available species records occurred. Biomes codes correspond to Olson et al. (2001): (A) Tropical & Subtropical Moist Broadleaf Forests; (B) Tropical & Subtropical Dry Broadleaf Forests; (C) Tropical & Subtropical Coniferous Forests; (D) Temperate Broadleaf & Mixed Forests; (E) Temperate Conifer Forests; (G) Tropical & Subtropical Grasslands, Savannas & Shrublands; (H) Temperate Grasslands, Savannas & Shrublands; (I) Flooded Grasslands & Savannas; (J) Montane Grasslands & Shrublands; (L) Mediterranean Forests, Woodlands & Scrub; (M) Deserts & Xeric Shrublands; (N) Mangroves.

code	species	locality	latitude	longitude	biome
S067	*Aetana abadae*	Philippines, Negros, Negros Oriental Prov., Casaroro Falls	9.281	123.208	A
S015	*Aetana baganihan*	Philippines, Mindanao, Marilog Distr., Baganihan	7.469	125.25	A
S075	*Aetana banahaw*	Philippines, Luzon, Mt. Banahaw, forest near Taytay Falls	14.11	121.507	A
GB01	*Aetana gaya*	Malaysia, Sabah, Gaya Island	6.016	116.02	A
S330	*Aetana Ind101*	Indonesia, West Papua, Manokwari, Gunung Meja	-0.86	134.084	A
S331	*Aetana Ind114*	Indonesia, Ternate, at Danau Tolire (lake)	0.83	127.311	A
S119	*Aetana indah*	Malaysia, Sabah, Crocker Range between Kota Kinabalu and Tambuan, N-slope	5.834	116.336	A/J
S117	*Aetana kinabalu*	Malaysia, Sabah, Mt. Kinabalu, forest along Silau Silau Trail	6.014	116.54	J
S221	*Aetana kiukoki*	Philippines, Camiguin, Mt. Hibok Hibok	9.196	124.692	A
S223	*Aetana kiukoki*	Philippines, Mindanao, Marilog Distr., Baganihan	7.469	125.25	A
GB04	*Aetana lambir*	Malaysia, Sarawak, Lambir Hills NP	4.203	114.04	A
S121	*Aetana lambir*	Malaysia, Sarawak, Lambir Hills NP	4.203	114.04	A
S080	*Aetana libjo*	Philippines, Dinagat, Paragua Forest, 'site 1'	10.222	125.553	A
GB31	*Aetana loboc*	Philippines, Bohol, near Loboc, above Loboc River	9.651	124.022	A
S069	*Aetana loboc*	Philippines, Bohol, near Loboc, above Loboc River	9.651	124.022	A
S077	*Aetana lozadae*	Philippines, Luzon, Mt. Isarog, W slope	13.664	123.34	A
GB32	*Aetana manansalai*	Philippines, Luzon, between Lucban and Tayabas	14.063	121.567	A
S073	*Aetana manansalai*	Philippines, Luzon, between Lucban and Tayabas	14.063	121.567	A
GB70	*Aetana ocampoi-morphA*	Philippines, Luzon, Mt. Isarog, W slope	13.664	123.34	A
S007	*Aetana ocampoi-morphA*	Philippines, Luzon, Mt. Isarog, W slope	13.664	123.34	A
S008	*Aetana ocampoi-morphB*	Philippines, Luzon, Mt. Isarog, W slope	13.664	123.34	A
GB02	*Aetana omayan*	Philippines, Luzon, Baguio, Crystal Cave	16.396	120.572	C
S001	*Aetana omayan*	Philippines, Luzon, Baguio, Crystal Cave	16.396	120.572	C
S079	*Aetana paragua*	Philippines, Dinagat, Paragua Forest, 'site 1'	10.222	125.553	A
S118	*Aetana poring*	Malaysia, Sabah, Mt. Kinabalu, forest above Kinabalu Mountain Lodge	6.013	116.534	J
OG26	*Agelenopsis aperta*	–			na
S002	*Anansus atewa*	Ghana, Eastern Region, Atewa Hills, Atewa Atwirebu Reserve	6.231	-0.558	A
S286	*Anansus ewe*	Ghana, Central Region, Kakum National Park	5.348	-1.384	A
P0190	*Anansus kamwai*	Cameroon, Northwest Region, near Bamenda	6.008	10.302	A
S309	*Anopsicus Car127*	Costa Rica, Heredia, La Selva Biol. Stn, 2014	10.423	-84.022	A
S310	*Anopsicus Car206*	Jamaica, Trelawny Parish, Trail to Upper Windsor Cave	18.348	-77.647	A
S413	*Anopsicus USA3*	USA, Texas, Big Bend National Park, Cat Tail Fall	29.273	-103.336	M
OG7	*Araneus diadematus*	–			na
OG34	*Argiope argentata*	–			na
OG6	*Ariadna fidicina*	–			na
BB01	*Artema atlanta*	USA, Florida, Sarasota	27.33	-82.526	SA
JA119	*Artema atlanta*	Egypt, Cairo	30	31.25	SA
S312	*Artema bunkpurugu*	Ghana, Northern Region, between Gambaga and Nakpanduri	10.567	-0.291	G
S326	*Artema doriai*	Israel, Judea and Samaria Distr., Memorial Ha-Biq'a, NE Peza'el	32.052	35.459	D
GB03	*Artema nephilit*	Israel, Northern Distr., Mount Berenice, S Teverya (Tiberias)	32.777	35.541	na
S325	*Artema nephilit*	Jordan, Ma'an Prov., Petra	30.324	35.447	na
S497	*Artema Om14*	Oman, Ad Dakhiliya, W of Bahla	22.934	57.084	H
S235	*Artema sp.C*	India, Rajastan, Durjana	29.008	74.822	M
S287	*Artema sp.C*	Sudan, Khartoum, Khartoum City	15.55	32.55	M
S332	*Artema transcaspica*	Tajikistan, Khatton Area, Khushody	37.152	68.07	M
OG4	*Austrochilus sp*	–			na
S414	*Aymaria Br16-188*	Brazil, Acre, Cruzeiro do Sul, forest at Fazenda Colorado, 'site 2'	-7.528	-72.655	A
P0231	*Belisana akebona*	Japan, Tokushima Perf., Naka-gun, Naka-chô, Sawadani	33.82	134.48	D
S238	*Belisana aninaj*	Thailand, Nakhon Si Thammarat, Khao Nan N.P., Tham Hong, 120 m a.s.l., degraded forest around cave, leaf litter	8.733	98.635	A
S415	*Belisana aninaj*	Thailand, Nakhon Si Thammarat, Khao Nan N.P., Tham Hong, 120 m a.s.l., degraded forest around cave, leaf litter	8.733	98.635	A
S230	*Belisana australis*	Philippines, Mindanao, Santo Domingo	7.782	125.397	G
S333	*Belisana bohorok*	Indonesia, Sumatra, Tangkahan, forest S of village	3.677	98.071	A
S125	*Belisana Bor121*	Malaysia, Sarawak, Bako National Park, along Lintang Trail	1.718	110.452	A/N
S243	*Belisana Bor121*	Singapore, MacRitchie Reservoir Park	1.355	103.813	A/N
S126	*Belisana Bor147*	Malaysia, Sabah, Mt. Kinabalu, forest above Kinabalu Mountain Lodge	6.013	116.534	J
S127	*Belisana Bor152*	Malaysia, Sabah, Gaya Island	6.016	116.02	A

code	species	locality	latitude	longitude	biome
S128	*Belisana Bor160*	Malaysia, Sabah, Crocker Range between Kota Kinabalu and Tambuan, N-slope	5.834	116.336	A
S129	*Belisana Bor85*	Malaysia, Sarawak, near Kapit	1.941	112.907	A
S334	*Belisana Ind15*	Indonesia, Sumatra, forested doline near Baso Cave	-0.252	100.482	A
S237	*Belisana junkoae*	Taiwan, Pingtung, near Chouqia	22.241	120.855	A
S239	*Belisana ketambe?*	Thailand, Narathiwat, Hala Bala Wildlife Sanctuary, 'site 1', forest at river near headquarters	5.797	101.832	A
S025	*Belisana Benj44*	Sri Lanka, Central Prov., Badulla Distr., Ohiya	6.82	80.844	A
S241	*Belisana khaoyai*	Thailand, Krabi, Khao Phanom Bencha National Park, trails near headquarters	8.235	98.918	A/B
S242	*Belisana leuser*	Thailand, Narathiwat, Hala Bala Wildlife Sanctuary, 'site 1', forest at river near headquarters	5.797	101.832	A
S244	*Belisana Mal30*	Malaysia, Johor, Gunung Ledang, forest near Puteri Falls	2.355	102.635	A
S416	*Belisana Mal35*	Malaysia, Johor, Gunung Ledang, forest near Puteri Falls	2.355	102.635	A
S246	*Belisana Mal55*	Malaysia, Pahang, Ulu Dong, forest near river	3.942	102.027	A
S247	*Belisana Mal76*	Malaysia, Pahang, Cameron Highlands, near Tanah Rata, forest along 'trail 9'	4.462	101.39	A
S248	*Belisana Mal77*	Malaysia, Pahang, Cameron Highlands, near Tanah Rata, forest along 'trail 9'	4.462	101.39	A
S249	*Belisana Mal91*	Malaysia, Kedah, Gunung Jerai, forest near Sri Perigi Waterfall	5.808	100.408	A
S336	*Belisana nahtanoj*	Indonesia, Sulawesi, between Bantimurung and Kappang	-5.049	119.738	A
S219	*Belisana Phi80*	Philippines, Dinagat, Paragua Forest, 'site 1'	10.222	125.553	A
P0282	*Belisana Raff32*	Singapore, Bukit Timah Nature Reserve	1.343	103.775	A
S417	*Belisana ranong*	Thailand, Ranong, Klong Nakha Wildlife Sanctuary	9.46	98.512	A
S386	*Belisana sabah*	Malaysia, Sabah, Mt. Kinabalu, forest above Kinabalu Mountain Lodge	6.013	116.534	J
S337	*Belisana sandakan*	Indonesia, Sumatra, forested doline near Baso Cave	-0.252	100.482	A
S498	*Belisana SL37*	Sri Lanka, North Central Prov., Minneriya Forest	8.047	80.832	B
S103	*Belisana Tai4*	Taiwan, Tainan, Shinhua National Forest	23.024	120.358	A
S338	*Belisana tambligan*	Indonesia, Bali, Lake Tambligan, forest along SE side of lake	-8.265	115.098	A
P0267	*Buitinga batwa*	Uganda, Kisoro Distr., Mgahinga Gorilla National Park, bamboo forest	-1.368	29.61	A
S339	*Buitinga buhoma*	Uganda, Kanungu Distr., Bwindi Impenetrable Forest National Park	-1	29.618	A
P0259	*Buitinga griswoldi*	Uganda, Bushenyi Distr., Kasyoha-Kitomi Forest Reserve	-0.268	30.152	G
P0191	*Buitinga nigrescens?*	Kenya, Eastern Prov., Ol Donyo Sabuk National Park	-1.135	37.257	G
S340	*Buitinga ruhiza*	Uganda, Kanungu Distr., Bwindi Impenetrable Forest National Park	-1	29.618	A/G
S341	*Buitinga ruwenzori*	Uganda, Kasese Distr., Ruwenzori Mts, near Nyabitaba Hut	0.358	29.978	J
S483	*Buitinga Uga5*	Uganda, Kasese Distr., Ruwenzori Mts, between Nyabitaba Hut and Guy Yeoman Hut	0.35	29.95	J
S484	*Buitinga Uga78*	Uganda, Kabarole Distr., Kibale Forest National Park, forest near Makerere Univ. Research Station	0.553	30.357	A
P0156	*Buitinga wataita*	Kenya, Coast Prov., Taita Hills, Ngangao Forest	-3.37	38.34	A
S251	*Calapnita anai*	Singapore, MacRitchie Reservoir Park	1.355	103.813	A
S418	*Calapnita bankirai*	Indonesia, East Kalimantan, Bukit Bankirai	-1.029	116.867	A
S419	*Calapnita bario*	Malaysia, Sarawak, Bario, forest along river N of town	3.768	114.446	A
S420	*Calapnita bidayuh*	Malaysia, Sarawak, near Kapit	1.941	112.907	A
S421	*Calapnita bohol*	Philippines, Bohol, near Loboc, at Loboc River	9.651	124.022	A
S133	*Calapnita deelemanae*	Malaysia, Sabah, Poring Hot Springs, forest near beginning of Kipungit Trail	6.048	116.706	A
S485	*Calapnita dinagat*	Philippines, Dinagat, Paragua Forest, 'site 1'	10.222	125.553	A
S184	*Calapnita kubah*	Malaysia, Sarawak, Kubah National Park, near entrance to Waterfall Trail	1.606	110.187	A
S136	*Calapnita lehi*	Malaysia, Sarawak, Lambir Hills National Park	4.203	114.04	A
S422	*Calapnita loksado*	Indonesia, South Kalimantan, Loksado	-2.796	115.503	A
S227	*Calapnita nunezae*	Philippines, Mindanao, Mt. Matutum, Kawit Forest, 'site 1'	6.338	125.104	A
S134	*Calapnita phyllicola*	Malaysia, Sarawak, Bako National Park, along Lintang Trail	1.718	110.452	A
S252	*Calapnita saluang*	Thailand, Krabi, Khao Phanom Bencha National Park, trails near headquarters	8.235	98.918	A
GB33	*Calapnita semengoh*	Malaysia, Sarawak, Semengoh NP	1.398	110.32	A
S135	*Calapnita semengoh*	Malaysia, Sarawak, Semengoh NP	1.398	110.32	A
S229	*Calapnita subphyllicola*	Philippines, Mindanao, near Santo Domingo, Penolohan	7.769	125.42	A
S076	*Calapnita vermiformis*	Philippines, Luzon, Mt. Banahaw, forest near Taytay Falls	14.11	121.507	A
S424	*Calapnita vermiformis?*	Philippines, Negros, Negros Oriental Prov., Twin Lakes National Park	9.366	123.181	A
OG37	*Callobius sp*	–			na
S344	*Carapoia alagoas*	Brazil, Alagoas, near Murici, degraded forest near road	-9.327	-35.91	A
S352	*Carapoia Br15-15*	Brazil, Bahia, Fazenda Morro de Pedra	-12.528	-40.604	B
S455	*Carapoia Br15-18*	Brazil, Bahia, Fazenda Morro de Pedra	-12.528	-40.604	B
S392	*Carapoia Br15-48*	Brazil, Alagoas, Reserva Biológica de Pedra Talhada	-9.238	-36.448	A
S354	*Carapoia Br15-62*	Brazil, Alagoas, Reserva Biológica de Pedra Talhada	-9.238	-36.448	A
S394	*Carapoia Br15-78*	Brazil, Pernambuco, near Bonito, forest near Cachoeira da Gruta	-8.547	-35.712	A
S393	*Carapoia Br15-79*	Brazil, Pernambuco, near Bonito, forest near Cachoeira da Gruta	-8.547	-35.712	A
S428	*Carapoia Br16-58*	Brazil, Pará, Floresta Nacional de Tapajós, km 67, 'site 1'	-2.847	-54.972	A
S429	*Carapoia Br16-67*	Brazil, Pará, Floresta Nacional de Tapajós, km 67, 'site 1'	-2.847	-54.972	A
S425	*Carapoia Br16-73*	Brazil, Pará, Floresta Nacional de Tapajós, km 67, 'site 2'	-2.875	-54.941	A
S035	*Carapoia brescoviti*	Brazil, Bahia, Res. Biol. de Una	-15.175	-39.058	A
GB34	*Carapoia capixaba*	Brazil, Espirito Santo, Res. Biol. de Sooretama, site 1	-19.055	-40.147	A

code	species	locality	latitude	longitude	biome
S027	*Carapoia capixaba*	Brazil, Espirito Santo, Res. Biol. de Sooretama, site 1	-19.055	-40.147	A
S345	*Carapoia carvalhoi*	Brazil, Bahia, near Santa Teresinha, Serra da Jibóia, near hilltop	-12.856	-39.477	A
S040	*Carapoia carybei*	Brazil, Bahia, Mata de São João	-12.467	-38.233	A
S346	*Carapoia crasto*	Brazil, Sergipe, near Santa Luzia do Itanhy, Mata do Crasto	-11.378	-37.418	A
GB05	*Carapoia dandarae*	Brazil, Bahia, Reserva Particular do Patrimônio Natural Serra Bonita	-15.388	-39.563	A
S033	*Carapoia dandarae*	Brazil, Bahia, Reserva Particular do Patrimônio Natural Serra Bonita	-15.388	-39.563	A
P0209	*Carapoia divisa*	Brazil, Rio de Janeiro, Santa Maria Madalena, forest fragment	-21.983	-41.957	A
S426	*Carapoia fowleri*	Brazil, Amazonas, Manaus, Reserva Ducke	-2.932	-59.97	A
P0205	*Carapoia genitalis*	Brazil, São Paulo, Paranapiacaba, Estação Biologica do Alto da Serra	-23.778	-46.31	A
GB35	*Carapoia gracilis*	Brazil, Bahia, Parque Nacional do Pau Brasil, site 2	-16.428	-39.352	A
S030	*Carapoia gracilis*	Brazil, Bahia, Parque Nacional do Pau Brasil, site 2	-16.428	-39.352	A
S343	*Carapoia jiboja*	Brazil, Bahia, near Santa Teresinha, Serra da Jibóia	-12.845	-39.489	A
P0206	*Carapoia macacu*	Brazil, Rio de Janeiro, ~3.5 km NW Parati	-23.192	-44.732	A
P0207	*Carapoia macacu*	Brazil, Rio de Janeiro, Cachoeiras de Macacu, Res. Ecol. Guapiaçú	-22.415	-42.745	A
S037	*Carapoia marceloi*	Brazil, Bahia, Res. Ecol. da Michelin, 'site 1'	-13.822	-39.198	A
S026	*Carapoia mirim*	Brazil, Espirito Santo, Res. Biol. de Sooretama, 'site 1'	-19.055	-40.147	A
P0211	*Carapoia nairae*	Brazil, Espirito Santo, Vargem Alta, Fazenda Monte Verde	-20.465	-40.995	A
S427	*Carapoia ocaina*	Brazil, Amazonas, forest near Tabatinga	-4.244	-69.925	A
BB02	*Carapoia paraguaensis*	Venezuela, Bolívar, km 44 from El Dorado	6.417	-61.642	A
JA97	*Carapoia paraguaensis*	Venezuela, Bolívar, km 44 from El Dorado	6.417	-61.642	A
S028	*Carapoia patafina*	Brazil, Espirito Santo, Res. Biol. Córrego do Veado, 'site 1'	-18.368	-40.138	A
S029	*Carapoia pau*	Brazil, Bahia, Parque Nacional do Pau Brasil, 'site 2'	-16.428	-39.352	A
S031	*Carapoia rheimsae*	Brazil, Bahia, Reserva Particular do Patrimônio Natural Serra Bonita	-15.388	-39.563	A
S288	*Carapoia RJ16*	Brazil, Rio de Janeiro, Macaé, Mata da Atalaia	-22.31	-42.003	A
S342	*Carapoia septentrionalis*	Brazil, Pernambuco, near São Vicente Ferrer, 'site 1'	-7.622	-35.465	A
JA102	*Carapoia ubatuba*	Brazil, São Paulo, Ubatuba, Fazenda Angelim	-22.615	-52.298	A
S036	*Carapoia una*	Brazil, Bahia, Res. Biol. de Una	-15.175	-39.058	A
S038	*Carapoia viridis*	Brazil, Bahia, Res. Ecol. da Michelin, 'site 2'	-13.842	-39.242	A
P0208	*Carapoia voltavelha*	Brazil, Santa Catarina, Itapoá, Reserva Volta Velha	-26.097	-48.652	A
S034	*Carapoia zumbii*	Brazil, Bahia, Reserva Particular do Patrimônio Natural Serra Bonita	-15.388	-39.563	A
S060	*Chibchea mapuche?*	Chile, Curicó, 30 km E Los Queñes	-35.063	-70.513	D
S058	*Chibchea picunche?*	Chile, Coquimbo, 4 km S Los Vilos	-31.936	-71.516	L
S430	*Chisosa diluta*	USA, Texas, Big Bend National Park, Hot Springs	29.177	-103.001	M
BB03	*Ciboneya antraia*	Cuba, Pinar del Río, Ceja de Francisco	22.45	-84	A
S313	*Ciboneya nuriae*	Cuba, Pinar del Río, Viñales, forest at Las Maravillas de Viñales	22.562	-83.833	A
S289	*'Coryssocnemis' iviei*	Mexico, Hidalgo, 2.5 km N Zacualtipan, road to Santiago Tianguiestengo	20.675	-98.669	C
JA100	*Coryssocnemis simla*	Venezuela, Sucre, Cascada el Chorro	10.392	-63.633	A
JA107	*Coryssocnemis simla*	Trinidad, Tunapuna-Piarco, Arima-Blanchisseuse Road	10.71	-61.29	A
P0154	*Crossopriza lyoni*	Guinea, Guinée Forestière, Kissidougou	9.187	-10.093	SA
SB026	*Crossopriza lyoni*	India, Maharashtra, Amravati, Melghat Tiger Reserve	21.04	77.2	SA
S499	*Crossopriza Om16*	Oman, Ad Dhahira, Ibri, Al Kittan Cave	23.298	56.511	M
S500	*Crossopriza Om20*	Oman, Ad Dhahira, near Miskin (between Ibri and Rusdaq)	23.496	56.838	H
S290	*Crossopriza pristina*	Sudan, Kassala, New Halfa	15.33	35.6	M
OG9	*Deinopis spinosa*	–			na
OG35	*Diguetia sp*	–			na
OG20	*Dysdera erythrina-fervida*	–			na
OG19	*Dysderocrates silvestris*	–			na
OG22	*Erigone dentosa*	–			na
OG1	*Euagrus chisoseus*	–			na
S323	*Gen. Cu12-325*	Cuba, Siboney			M
S329	*Gen.n. Br15-159*	Brazil, Rio Grande do Norte, near Baraúna, Caverna Porco do Mato II	-5.047	-37.54	M
S353	*Gen.n. Br15-45*	Brazil, Alagoas, near Murici, Estação Ecológica de Murici	-9.247	-35.838	A
S433	*Gen.n. Br16-178*	Brazil, Acre, Cruzeiro do Sul, forest at Fazenda Colorado, 'site 1'	-7.525	-72.65	A
S431	*Gen.n. Br16-196*	Brazil, Amazonas, forest near Tabatinga	-4.244	-69.925	A
S464	*Gen.n. Br16-44*	Brazil, Amapá, forest SW Macapá, 'site 2'	-0.051	-51.123	G
S432	*Gen.n. Br16-50*	Brazil, Amapá, forest SW Macapá, 'site 3'	-0.138	-51.367	A/G
S006	*Gen.n. Geneve59*	Curaçao, Jan Kok Lodges	12.217	-69.051	M
S468	*Gen.n. Ind206*	Indonesia, Halmahera, degraded forest along river near Sofifi	0.715	127.586	A
S357	*Gen.n. MACN270*	Argentina, San Luis, Villa de Merlo, Parque Recr. Pasos Malos	-32.319	-64.972	G
S358	*Gen.n. MACN273*	Bolivia, Santa Cruz, Yabaré	-16.442	-62.173	B
S501	*Gen.n. Om6*	Oman, Ash Sharqiyah South, between Sur and Al Kamil	22.462	59.388	M
JA83	*Gen.n. Ven01*	Venezuela, Bolívar, Canaima, Salto el Sapo	6.242	-62.85	G
S059	*Gertschiola macrostyla*	Argentina, San Juan, Valle Fértil, Chucuma	-31.037	-67.286	J
S434	*Gertschiola macrostyla*	Argentina, San Luis, Sierra de las Quijadas National Park	-32.494	-66.963	J
S347	*Guaranita yaculica?*	Argentina, Jujuy, Calilegua National Park	-23.762	-64.851	A
S122	*Hantu kapit*	Malaysia, Sarawak, near Kapit	1.941	112.907	A
S412	*Hantu kapit*	Malaysia, Sarawak, near Kapit	1.941	112.907	A
S322	*Hantu niah*	Malaysia, Sarawak, Niah Cave National Park, forest near cave	3.814	113.771	A
OG10	*Harpactea hombergi*	–			na
OG36	*Harpactocrates radulifer*	–			na
OG18	*Herennia multipuncta*	–			na
S348	*Holocneminus huangdi*	Indonesia, Sumatra, Ngalau Indah Cave near Payakumbuh	-0.255	100.603	A

code	species	locality	latitude	longitude	biome
S486	*Holocneminus Ind73*	Indonesia, Sulawesi, between Bantimurung and Kappang	-5.049	119.738	A
S253	*Holocneminus multiguttatus*	Singapore, Upper Selatar Reservoir Park	1.4	103.807	A
S254	*Holocneminus multiguttatus*	Malaysia, Pulau Pinang, Penang National Park near Teluk Bahang	5.462	100.202	A
S016	*Holocneminus Phi105*	Philippines, Mindanao, Iligan, NPC Nature's Park near Cristina Falls	8.186	124.192	A
S078	*Holocneminus Phi74*	Philippines, Dinagat, Paragua Forest, 'site 1'	10.222	125.553	A
S068	*Holocneminus Phi9*	Philippines, Negros, Negros Oriental Prov., Twin Lakes National Park	9.366	123.181	A
S435	*Holocnemus caudatus*	Morocco, Souss-Massa-Draa, Imouzzer, N Agadir	30.678	-9.483	L
P0233	*Holocnemus hispanicus*	Spain, Larrache			L
S436	*Hoplopholcus asiaeminoris*	Turkey, Konya Prov., Hadim District, Su Ç?kt??? Ma?aras?	36.978	32.401	L
S009	*Hoplopholcus cecconii*	Israel, Haifa Distr., Bét She'arim	32.703	35.129	D
S437	*Hoplopholcus Del5*	Turkey, Antalya Prov., Gazipa?a District, Yalan Dünya Ma?aras?	36.221	32.402	L
S057	*Hoplopholcus forskali*	Hungary, Csongrád, Szeged	46.25	20.15	D
S316	*Hoplopholcus labyrinthi*	Greece, Crete, Melidoni Cave	35.385	24.73	L
S438	*Hoplopholcus Mar66*	Turkey, Antalya Prov., Alanya District, Dim Ma?aras?	36.539	32.11	L
S315	*Hoplopholcus minotaurinus*	Greece, Crete, Milatos Cave	35.308	25.578	L
S314	*Hoplopholcus minous*	Greece, Crete, Exo Mouliana, along path to Richtis Waterfall	35.182	25.988	L
S439	*Hoplopholcus patrizii*	Turkey, Antalya Prov., Dö?emealt? District, Kocain Ma?aras?	37.233	30.712	L
S440	*Hoplopholcus Tur21*	Turkey, Mersin Prov., Ayd?nc?k District, Aynal?göl (Gilindere) Ma?aras?	36.131	33.403	L
S441	*Hoplopholcus Tur32*	Turkey, Antalya Prov., Finike District, Sulu ?n Ma?aras? (Gök Ma?ara)	36.282	30.15	L
S442	*Hoplopholcus Tur7*	Turkey, Konya Prov., Derebucak District, Körükini Ma?aras?	37.348	31.628	L
OG27	*Hyptiotes gertschi*	–			na
S443	*Ibotyporanga Br16-149*	Brazil, Rondônia, Floresta Nacional de Jamari, Gran Piedra	-9.198	-63.082	A
JA123	*Ibotyporanga naideae*	Brazil, São Paulo, Campinas	-22.9	-47.07	A
Ixc29	*Ixchela abernathyi*	Mexico, San Luis Potosí, Km 227 Carretera Federal No. 70 Ciudad San Luis Potosí-Río Verde	22.088	-100.65	C
Ixc51	*Ixchela santibanezi*	Mexico, Chiapas, Municipio San Fernando, Cueva de las Abejas, outside cave	16.849	-93.243	A
Ixc39	*Ixchela taxco*	Mexico, Guerrero, "Rancho La Soñadora", 6 km W Taxco	18.561	-99.636	C
Ixc56	*Ixchela viquezi*	Honduras, Francisco Morazán, Parque Nacional La Tigra, 10 km NE Tegucigalpa	14.212	-87.095	A
S328	*Kambiwa neotropica*	Brazil, Rio Grande do Norte, near Felipe Guerra, Lajedo do Arapuá	-5.529	-37.614	M/N
S318	*Khorata circularis*	Laos, Vientiane, Lom Cave, 4.01 km N Vang Vieng	18.958	102.437	A
S319	*Khorata dupla*	Laos, Champasak, Tad Fane Resort	15.182	106.127	A
P0223	*Khorata khammouan*	Laos, Khammouane, Thakek	17.43	104.87	A
OG24	*Kukulcania hibernalis*	–			na
S255	*Leptopholcus borneensis*	Thailand, Kanchanaburi, Erawan National Park, forest along stream	14.37	99.146	A
GB36	*Leptopholcus budongo*	Uganda, Masindi Distr., Budongo Forest Reserve	1.717	31.535	A
P0260	*Leptopholcus budongo*	Uganda, Masindi Distr., Budongo Forest Reserve	1.717	31.535	A
P0237	*Leptopholcus cf griswoldi*	Madagascar, Toamasina, Station Forestier Analamazaotra, 0.75 km N Andasibe	-18.93	48.412	A
P0176	*Leptopholcus dschang*	Cameroon, Southwest Region, near Dschang, Attuleh, site 2	5.465	9.942	A
P0192	*Leptopholcus dschang*	Cameroon, Southwest Region, near Dschang, Attuleh, site 2	5.465	9.942	A
GB07	*Leptopholcus gabonicus*	Gabon, Ogooué-Ivindo, Monts de Belinga, forest near Mayebout	1.112	13.11	A
P0160	*Leptopholcus gracilis*	Kenya, Coast Prov., Gedi Forest near Gedi ruins	-3.308	40.018	A
P0220	*Leptopholcus gracilis?*	South Africa, KwaZulu Natal, Bonamanzi Reserve	-28.067	32.3	A
P0150	*Leptopholcus guineensis*	Guinea, Moyenne-Guinée, SE Mamou	10.293	-11.942	A
SB070	*Leptopholcus kandy*	Sri Lanka, Central Prov., Matale distric, Bowatenna Reservoir area	7.66	80.688	A
SB071	*Leptopholcus kandy*	Sri Lanka, Southern Prov., Galle District, Hiyare, Kombala-Kottawa FR, 252m	6.06	80.32	A
GB08	*Leptopholcus kintampo*	Ghana, Brong-Ahafo Region, Kintampo Falls	8.088	-1.698	G
P0235	*Leptopholcus MRAC530*	Congo DR, Equator, Mbangi, old secondary forest	2.117	21.733	A
P0279	*Leptopholcus podophthalmus*	Singapore, Clementi Rd (near Clementi Neighbourhood Park)	1.326	103.775	A
S349	*Leptopholcus podophthalmus*	Malaysia, Pahang, Cameron Highlands, forest along 'trail 9'	4.46	101.392	A
SB068	*Leptopholcus podophthalmus*	Sri Lanka, Central Prov., Matale District, Bowatenna Reservoir area	7.66	80.688	A
P0146	*Leptopholcus tipula*	Guinea, Guinée Forestière, Forêt Classée de Ziama	8.403	-9.322	A
P0169	*Leptopholcus tipula*	Cameroon, South Region, between Kribi and Campo, 'site 1'	2.703	9.863	A
P0236	*Leptopholcus tipula*	Congo DR, Orientale, Monzé (Engengele) sec. forest	2.033	22.733	A
P0261	*Leptopholcus tipula*	Uganda, Bushenyi Distr., Kyambura River Gorge	-0.178	30.097	A
OG11	*Leucauge venusta_B*	–			na
OG30	*Linyphia triangularis_A*	–			na
S351	*Litoporus Br15-107*	Brazil, Paraíba, near Queimadas, rocky outcrop near road	-7.351	-35.902	M
S444	*Litoporus Br16-153*	Brazil, Acre, Rio Branco, Fazenda Experimental Catuaba	-10.08	-67.625	A
S445	*Litoporus Br16-62*	Brazil, Pará, Floresta Nacional de Tapajós, km 67, 'site 1'	-2.847	-54.972	A
S446	*Litoporus dimona*	Brazil, Amazonas, Manaus, Reserva Ducke	-2.932	-59.97	A
P0212	*Litoporus iguassuensis*	Brazil, Espirito Santo, Vargem Alta, Fazenda Monte Verde	-20.465	-40.995	A

code	species	locality	latitude	longitude	biome
P0213	*Litoporus iguassuensis*	Brazil, Rio de Janeiro, Santa Maria Madalena, forest fragment	-21.983	-41.957	A
S447	*Litoporus manu*	Brazil, Amazonas, forest near Humaitá, 'site 3'	-7.512	-63.393	A
OG17	*Loxosceles laeta*	–			na
S448	*Mecolaesthus arima*	Trinidad, Couva-Tabaquite-Talparo, Arena Forest	10.56	-61.23	A
JA89	*Mecolaesthus longissimus*	Venezuela, Aragua, Colonia Tovar	10.417	-67.3	A
S294	*Mecolaesthus nigrifrons?*	Martinique, Saint Pierre, Mome Rose Trail	14.673	-61.123	A
S449	*Mecolaesthus taino?*	Dominica, St. Paul Parish, Middleham Falls Trail	15.341	-61.334	A
S295	*Mecolaesthus Ven02/80-12*	Venezuela, Lara, Parque Nacional Yacambú	9.708	-69.583	A
S450	*Mecolaesthus yawaperi*	Brazil, Amazonas, Manaus, Reserva Ducke	-2.932	-59.97	A
BB09a	*Mesabolivar aurantiacus*	Trinidad, Couva-Tabaquite-Talparo, Arena Forest	10.56	-61.23	A
S388	*Mesabolivar azureus*	Brazil, Rio de Janeiro, Cachoeiras de Macacu, Res. Ecol. Guapiaçú	-22.415	-42.745	A
GB09	*Mesabolivar bonita*	Brazil, Bahia, Reserva Particular do Patrimônio Natural Serra Bonita	-15.388	-39.563	A
S487	*Mesabolivar Br11-10*	Brazil, Espirito Santo, Res. Biol. de Sooretama, 'site 1'	-19.055	-40.147	A
S350	*Mesabolivar Br14-25*	Brazil, Parana, Iratí Forest Reserve	-25.362	-50.584	A
S390	*Mesabolivar Br14-26*	Brazil, Santa Catarina, Três Barras National Forest	-26.242	-50.301	A
S391	*Mesabolivar Br14-30*	Brazil, Santa Catarina, Serra do Itajaí National Park	-27.058	-49.084	A
S389	*Mesabolivar Br14-4*	Brazil, Parana, Saint-Hilaire/Lange National Park, above Fazenda Niteroi	-25.657	-48.601	A
S305	*Mesabolivar Br14-55*	Brazil, Santa Catarina, Serra Geral National Park, Churriado Canyon	-29.137	-49.953	A
S451	*Mesabolivar Br16-138*	Brazil, Rondônia, Floresta Nacional de Jamari, 'sites 2 & 3'	-9.24	-62.925	A
S452	*Mesabolivar Br16-32*	Brazil, Pará, Marabá, forest near road, 'site 2'	-5.36	-49.058	A
S453	*Mesabolivar Br16-60*	Brazil, Pará, Floresta Nacional de Tapajós, km 83	-3.049	-54.928	A
BB10	*Mesabolivar brasiliensis*	Brazil, São Paulo, São Paulo, Parque Estadual de Cantareira	-23.4	-46.58	A
S456	*Mesabolivar cambridgei*	Brazil, Pará, Belém, Parque Estadual do Utinga, 'site 1'	-1.424	-48.429	A
JA50	*Mesabolivar camussi*	Brazil, São Paulo, São Paulo, Parque Estadual de Cantareira	-23.4	-46.58	A
JA10	*Mesabolivar cavicelatus*	Brazil, São Paulo, Ubatuba, Fazenda Angelim	-22.615	-52.298	A
S395	*Mesabolivar charrua*	Brazil, Rio Grande do Sul, São Francisco de Paula National Forest	-29.43	-50.392	G
JA52	*Mesabolivar cyaneomaculatus*	Brazil, São Paulo, Paranapiacaba, Estação Biologica do Alto da Serra	-23.778	-46.31	A
BB11	*Mesabolivar cyaneotaeniatus*	Brazil, Rio de Janeiro, Itatiaia	-22.496	-44.561	A
JA57	*Mesabolivar cyaneotaeniatus*	Brazil, São Paulo, São Paulo, Jardim Zoologico	-23.651	-46.621	A
JA34	*Mesabolivar eberhardi*	Venezuela, Dist. Federal, Parque Nacional El Ávila	10.517	-66.85	A
JA93	*Mesabolivar eberhardi*	Venezuela, Bolívar, Canaima, near Salto Ara	6.242	-62.85	A
JA53	*Mesabolivar gabettae*	Brazil, São Paulo, Paranapiacaba, Estação Biologica do Alto da Serra	-23.778	-46.31	A
S410	*Mesabolivar goitaca*	Brazil, Rio de Janeiro, ~4 km NW Penedo	-22.408	-44.553	A
S454	*Mesabolivar huambisa?*	Brazil, Pará, Belém, Parque Estadual do Utinga, 'site 1'	-1.424	-48.429	A
S396	*Mesabolivar huberi*	Brazil, Santa Catarina, Itajaí National Park, 3a Vargem Trail	-27.065	-49.098	A
P0215	*Mesabolivar itapoa*	Brazil, Santa Catarina, Itapoá, Reserva Volta Velha	-26.097	-48.652	A
S387	*Mesabolivar kathrinae*	Brazil, Alagoas, near Murici, Estação Ecológica de Murici	-9.247	-35.838	A
JA41	*Mesabolivar luteus*	Brazil, Minas Gerais, Catas Altas, Serra do Caraca	-20.08	-43.42	A
JA19	*Mesabolivar mairyara*	Brazil, São Paulo, São Paulo, Jardim Zoologico	-23.651	-46.621	A/G
GB10	*Mesabolivar pau*	Brazil, Bahia, Parque Nacional do Pau Brasil, 'site 2'	-16.428	-39.352	A
GB11	*Mesabolivar sai*	Brazil, Rio de Janeiro, Res. Ecol. Rio das Pedras	-22.992	-44.107	A
S397	*Mesabolivar spinulosus*	Brazil, Rio Grande do Norte, Caatinga near João Câmara	-5.572	-35.921	M
JA29	*Mesabolivar tamoio*	Brazil, São Paulo, Ubatuba, Fazenda Angelim	-22.615	-52.298	A
JA54	*Mesabolivar togatus*	Brazil, São Paulo, São Paulo, Jardim Zoologico	-23.651	-46.621	A
P0217	*Mesabolivar unicornis*	Brazil, São Paulo, Paranapiacaba, Estação Biologica do Alto da Serra	-23.778	-46.31	A
BB07	*Mesabolivar yuruani*	Venezuela, Bolívar, km 109 from El Dorado	6.017	-61.392	H
JA101	*Mesabolivar yuruani*	Venezuela, Bolívar, km 109 from El Dorado	6.017	-61.392	H
S398	*Metagonia argentinensis*	Brazil, Rio Grande do Sul, Passo Fundo National Forest	-28.289	-52.187	A
JA78	*Metagonia belize*	Belize, Stann Creek, Cockcomb Basin Wildlife Refuge	16.833	-88.5	A
S457	*Metagonia beni*	Brazil, Acre, Cruzeiro do Sul, forest near airport	-7.596	-72.767	A
P0198	*Metagonia bifida?*	Brazil, Rio de Janeiro, ~4 km NW Penedo	-22.408	-44.553	A
JA12	*Metagonia Br03/80-25*	Brazil, São Paulo, São Paulo, Parque Estadual de Cantareira	-23.4	-46.58	A
BB13	*Metagonia Br03/80-25*	Brazil, São Paulo, São Paulo, Parque Estadual de Cantareira	-23.4	-46.58	A
P0199	*Metagonia Br07/80-1*	Brazil, Rio de Janeiro, ~4 km NW Penedo	-22.408	-44.553	A
P0277	*Metagonia Br09-2*	Brazil, Rio de Janeiro, Cachoeiras de Macacu, Res. Ecol. Guapiaçú	-22.415	-42.745	A
P0197	*Metagonia Br09-4*	Brazil, Rio de Janeiro, Cachoeiras de Macacu, Res. Ecol. Guapiaçú	-22.415	-42.745	A
JA27	*Metagonia Br09-52*	Brazil, São Paulo, Ubatuba, Fazenda Angelim	-22.615	-52.298	A
JA11	*Metagonia Br09-55*	Brazil, São Paulo, Ubatuba, Fazenda Angelim	-22.615	-52.298	N
S488	*Metagonia Br10-38*	Brazil, Espirito Santo, Vargem Alta, Fazenda Monte Verde	-20.465	-40.995	A
S489	*Metagonia Br11-11*	Brazil, Bahia, Parque Nacional do Pau Brasil, 'site 2'	-16.428	-39.352	A
S458	*Metagonia Br15-101*	Brazil, Pernambuco, near São Vicente Ferrer, 'site 1'	-7.622	-35.465	M
S459	*Metagonia Br16-125*	Brazil, Rondônia, Floresta Nacional de Jamari, 'site 1'	-9.193	-63.107	A
S460	*Metagonia Br16-180*	Brazil, Amazonas, forest near Tabatinga	-4.244	-69.925	A
S490	*Metagonia Br16-205*	Brazil, Amazonas, forest near Tabatinga	-4.244	-69.925	A
S461	*Metagonia Br16-216*	Brazil, Amazonas, Manaus, Reserva Ducke	-2.932	-59.97	A
JA117	*Metagonia delicata*	Belize, Stann Creek, Cockcomb Basin Wildlife Refuge	16.833	-88.5	A
P0216	*Metagonia furcata*	Brazil, Espirito Santo, Mimoso do Sul, Finca Tacutinga	-21.023	-41.39	A

code	species	locality	latitude	longitude	biome
BB14	*Metagonia G062*	Brazil, São Paulo, Iporanga, PETAR	-24.552	-48.68	A
S462	*Metagonia globulosa*	Brazil, Pará, Marabá, forest near road, 'site 1'	-5.414	-49.074	A
S399	*Metagonia jamaica*	Jamaica, Clarendon Parish, Jackson Bay, 3rd Cave	17.734	-77.222	B
JA23	*Metagonia mariguitarensis*	Venezuela, Sucre, Mariguitar	10.442	-63.908	G
JA15	*Metagonia paranapiacaba*	Brazil, São Paulo, Paranapiacaba, Estação Biologica do Alto da Serra	-23.778	-46.31	A
S400	*Metagonia potiguar*	Brazil, Rio Grande do Norte, near Felipe Guerra, Lajedo do Arapuá, Caverna do Sabonete	-5.527	-37.624	M
S401	*Metagonia rica?*	Costa Rica, Heredia, La Selva Biol. Stn, 2014	10.423	-84.022	A
S039	*Metagonia taruma*	Brazil, Bahia, Mata de São João	-12.467	-38.233	A
S463	*Metagonia USA2*	USA, Texas, Big Bend National Park, Cat Tail Fall	29.273	-103.336	M
JA85	*Metagonia Ven02*	Venezuela, Bolívar, Canaima, village forest	6.242	-62.85	G
JA8	*Metagonia Ven54*	Venezuela, Bolívar, km 109 from El Dorado	6.017	-61.392	A
S355	*Micromerys baiteta*	Indonesia, West Papua, Manokwari, Gunung Meja	-0.86	134.084	A
S296	*Micromerys raveni*	Australia, New South Wales, Bunjalung National Park, Woody Head	-29.368	153.37	D
S297	*Micromerys yidin*	Australia, Queensland, Kings Plains, Cooktown	-15.485	145.256	A
GB37	*Micropholcus baoruco*	Dom. Rep., Barahona, near Polo	18.113	-71.27	B
P0166	*Micropholcus baoruco*	Dom. Rep., Barahona, near Polo	18.113	-71.27	B
Mic02	*Micropholcus dalei*	Puerto Rico, Rio Grande, El Yunque, Big Trees Trail	18.309	-65.775	A
Mic16	*Micropholcus delicatulus*	Cuba, Santiago de Cuba, Estación Biológica La Platica, forest near brook	20.009	-76.894	A
P0193	*Micropholcus fauroti*	Cameroon, Centre Region, Yaoundé, hotel	3.883	11.523	SA
P0165	*Micropholcus hispaniola*	Dom. Rep., La Vega, near La Ciénaga, along river	19.05	-70.883	A
S228	*Micropholcus Phi114*	Philippines, Mindanao, Central Mindanao Univ., Faculty Hill	7.853	125.049	A
GB38	*Micropholcus piaui*	Brazil, Piauí, Castelo do Piauí, Parque Municipal da Pedra de Castelo	-5.202	-41.688	na
Mic10	*Micropholcus piaui*	Brazil, Piauí, Castelo do Piauí, Parque Municipal da Pedra de Castelo	-5.202	-41.688	na
P0167	*Micropholcus toma?*	Haiti, Dept Sud, near Le Prete	18.265	-73.945	A
GB39	*Micropholcus ubajara*	Brazil, Ceará, Parque Nacional de Ubajara, Gruta de Morcego Branco	-3.833	-40.9	A
Mic11	*Micropholcus ubajara*	Brazil, Ceará, Parque Nacional de Ubajara, Gruta de Morcego Branco	-3.833	-40.9	A
NF69	*Modisimus berac*	Haiti, Dept Sud-Est, La Visite National Park, 'Berac'	18.328	-72.295	C
S356	*Modisimus cavaticus*	Puerto Rico, Aguas Buenas, Cuevas Aguas Buenas	18.232	-66.109	A
NF13	*Modisimus cienaga*	Dom. Rep., La Vega, near La Ciénaga, along river	19.05	-70.883	C
IA03	*Modisimus Cu12-11*	Cuba, Pinar del Río, forest at km 14 from Pinar del Rio to Viñales	22.535	-83.7	C
S106	*Modisimus Cu12-11*	Cuba, Pinar del Río, forest at km 14 from Pinar del Rio to Viñales	22.535	-83.7	C
IA04	*Modisimus Cu12-136*	Cuba, Santiago de Cuba, Estación Biológica La Platica, pine forest along mountain crest	20.011	-76.884	A
NF3	*Modisimus cuadro*	Dom. Rep., San Cristóbal, near La Toma	18.458	-70.12	A
IA01	*Modisimus culicinus*	Cuba, Camagüey, Sierra de Cubitas, Estación Limones-Tuabaquey	21.591	-77.788	SA
NF43	*Modisimus culicinus*	Dom. Rep., San Pedro de Macorís, Juan Dolio	18.425	-69.427	SA
IA05	*Modisimus elongatus*	Cuba, Pinar del Río, Viñales, forest at base of mogote, 'site 1'	22.622	-83.737	A
NF101	*Modisimus enriquillo*	Dom. Rep., Barahona, NW Paraiso	18.04	-71.193	B
NF80	*Modisimus epepye*	Haiti, Dept Sud-Est, near Seguin	18.303	-72.292	C
NF66	*Modisimus fuscus*	Haiti, Dept Sud-Est, La Visite National Park, Case Dent	18.337	-72.273	A
P0228	*Modisimus G106*	Panama, Chiriqui, Parque Nacional de la Amistad	8.892	-82.618	A
NF9	*Modisimus kiskeya*	Dom. Rep., Monseñor Nouel, near Jima	19.023	-70.48	A
NF87	*Modisimus leprete*	Haiti, Dept Sud, near Le Prete	18.265	-73.945	A
IA02	*Modisimus macaya*	Haiti, Dept Sud, Macaya Biosphere Reserve	18.34	-74.015	A
NF37	*Modisimus makandal*	Dom. Rep., Santiago Rodríguez, near Cana	19.497	-71.282	A
NF89	*Modisimus mango*	Haiti, Dept Sud, near Le Prete	18.265	-73.945	A
NF24	*Modisimus miri*	Dom. Rep., Duarte, Reserva Científica Loma Quita Espuela	19.358	-70.15	A/C
NF64	*Modisimus palvet*	Haiti, Dept Sud, Macaya Biosphere Reserve	18.34	-74.015	A
NF53	*Modisimus paraiso*	Dom. Rep., Barahona, NW Paraiso	18.04	-71.193	B
S113	*Modisimus pavidus*	Cuba, Santiago de Cuba, Estación Biológica La Platica, forest along mountain crest	20.01	-76.883	A
NF71	*Modisimus roumaini*	Haiti, Dept Sud-Est, between Léogâne and Jacmel	18.357	-72.583	A
NF81	*Modisimus seguin*	Haiti, Dept Sud-Est, near Seguin	18.303	-72.292	C
S231	*Modisimus signatus*	Puerto Rico, Rio Grande, El Yunque, El Verde	18.322	-65.82	A
NF68	*Modisimus tiburon*	Haiti, Dept Sud-Est, La Visite National Park, 'Berac'	18.328	-72.295	C
NF1	*Modisimus toma*	Dom. Rep., San Cristóbal, near La Toma	18.458	-70.12	A
NF41	*Modisimus vittatus*	Dom. Rep., Barahona, near Cabral	18.243	-71.197	B
OG8	*Nephila clavipes_A*	–			na
S061	*Nerudia Mich20*	Argentina, San Juan, Valle Fértil, Parque Natural Valle Fértil	-30.638	-67.489	G
BB16	*Ninetis subtilissima*	Yemen, Ja'ar	13.223	45.305	M
P0234	*Nita elsaff*	Egypt, Cairo	30	31.25	M
P0144	*Nyikoa limbe*	Guinea, Guinée Forestière, Forêt Classée de Diéké	7.533	-8.832	A
P0179	*Nyikoa limbe*	Cameroon, South Region, near Kribi	2.9	9.907	A
OG2	*Oecobius sp*	–			na
S320	*Otavaloa cf angotero*	Ecuador, Napo, Napo-Galeras National Park, betw. Tena and Loreto	-0.733	-77.591	A
S465	*Otavaloa lisei*	Brazil, Amazonas, forest near Tabatinga	-4.244	-69.925	A
S021	*Otavaloa piro?*	Bolivia, La Paz, Rurrenabaque, Parque Nacional Madidi & surroundngs	-14.467	-67.567	A
S359	*Panjange alba*	Indonesia, Sulawesi, Tinoor Fall near Tomohon	1.382	124.836	A
S466	*Panjange camiguin*	Philippines, Camiguin, Katibawasan Falls	9.215	124.72	A
S066	*Panjange casaroro*	Philippines, Negros, Negros Oriental Prov., Casaroro Falls	9.281	123.208	A

code	species	locality	latitude	longitude	biome
S360	*Panjange cavicola*	Indonesia, Sulawesi, Bantimurung, at rocks along river	-5.015	119.686	A
S218	*Panjange dinagat*	Philippines, Dinagat, Paragua Forest, 'site 1'	10.222	125.553	A
GB40	*Panjange iban*	Malaysia, Sarawak, Semengoh NP	1.398	110.32	A
S169	*Panjange iban*	Malaysia, Sarawak, Semengoh NP	1.398	110.32	A
S361	*Panjange Ind103*	Indonesia, West Papua, Manokwari, Gunung Meja	-0.86	134.084	A
S362	*Panjange Ind109*	Indonesia, Halmahera, degraded forest along river near Sofifi	0.715	127.586	A
S173	*Panjange kapit*	Malaysia, Sarawak, near Kapit	1.941	112.907	A
GB41	*Panjange kubah*	Malaysia, Sarawak, Kubah NP	1.601	110.184	A
S171	*Panjange kubah*	Malaysia, Sarawak, Kubah NP	1.601	110.184	A
S010	*Panjange lanthana*	Philippines, Luzon, Mt. Isarog, W slope	13.664	123.34	A
S224	*Panjange malagos*	Philippines, Mindanao, Malagos Watershed	7.184	125.415	A
S222	*Panjange marilog*	Philippines, Mindanao, Marilog Distr., Baganihan	7.469	125.25	A
S172	*Panjange niah?*	Malaysia, Sarawak, Niah Cave National Park	3.818	113.768	A
S174	*Panjange pueh*	Malaysia, Sarawak, Pueh foothills	1.799	109.713	A
GB42	*Panjange seowi*	Malaysia, Sarawak, Mt. Penrissen	1.126	110.217	A
S170	*Panjange seowi*	Malaysia, Sarawak, Mt. Penrissen	1.126	110.217	A
S011	*Papiamenta MRAC639*	Curaçao, near San Juan, Manzalina beach	12.245	-69.105	M
P0245	*Paramicromerys betsileo?*	Madagascar, Fianarantsoa, Ranomafana National Park, Vatoharanana, 4 km S Ranomafana	-21.288	47.427	A
P0244	*Paramicromerys CAS11*	Madagascar, Toamasina, Station Forestier Analamazaotra, 0.75 km N Andasibe	-18.93	48.412	A
S363	*Paramicromerys CAS17*	Madagascar, Fianarantsoa, Forêt d'Antsirakambiaty	-20.594	46.564	A
P0238	*Paramicromerys CAS3*	Madagascar, Fianarantsoa, Andringitra National Park, 34 km S Ambalavao	-22.147	46.952	A
P0221	*Paramicromerys G046*	Comoros, Anjouan, Domoni, road to Tatinga bridge	-12.182	44.512	A
S383	*Paramicromerys? CAS13*	Madagascar, Toliara, Forêt Classée Tsitongambarika	-24.987	46.927	A
S467	*Paramicromerys? CAS4*	Madagascar, Fianarantsoa, Andringitra National Park, 34 km S Ambalavao	-22.157	46.952	A
P0151	*Pehrforsskalia conopyga*	Guinea, Moyenne-Guinée, near Sêbori	10.777	-12.29	A
P0168	*Pehrforsskalia conopyga*	Cameroon, Northwest Region, near Bamenda	6.008	10.302	A
JA45	*Pholcophora americana*	USA, California, Mono County, Inyo Nat. Forest	37.8	-118.38	E
JA46	*Pholcophora americana*	USA, Montana, Missoula Co., Lolo Forest	47.072	-113.384	E
S298	*Pholcophora? Car544*	Puerto Rico, Isla Monito	18.16	-67.95	B
S256	*'Pholcus gracillimus' sensu Huber 2011*	Malaysia, Pahang, Cameron Highlands, near Tanah Rata, forest along 'trail 9'	4.462	101.39	A
S257	*'Pholcus kohi' sensu Huber 2011*	Singapore, MacRitchie Reservoir Park	1.355	103.813	A
S321	*Pholcus agadir*	Morocco, Souss-Massa-Draa, Agadir	30.43	-9.619	L
S178	*Pholcus andulau*	Malaysia, Sarawak, Gunung Mulu NP	4.037	114.823	A
S469	*Pholcus andulau*	Malaysia, Sarawak, Gunung Mulu NP	4.037	114.823	A
S071	*Pholcus arayat*	Philippines, Luzon, near Olangapo, along river near Pamulaklakin Forest Trail	14.798	121.338	A
P0281	*Pholcus atrigularis*	Singapore, Bukit Timah Nature Reserve	1.343	103.775	A
P0177	*Pholcus attuleh*	Cameroon, Southwest Region, near Dschang, Attuleh, 'site 2'	5.465	9.942	A
S070	*Pholcus baguio*	Philippines, Luzon, near Baguio, Mt. Kabuyao, N slope	16.374	120.557	C
P0196	*Pholcus baka*	Cameroon, South Region, between Kribi and Campo, 'site 1'	2.703	9.863	A
GB43	*Pholcus bamboutos*	Cameroon, West Region, near Mbouda, Bamboutos	5.622	10.112	A
P0181	*Pholcus bamboutos*	Cameroon, West Region, near Mbouda, Bamboutos	5.622	10.112	A
P0195	*Pholcus bamboutos*	Cameroon, Northwest Region, near Lake Oku	6.187	10.465	A
S470	*Pholcus bario*	Malaysia, Sarawak, Bario, forest along river W of town	3.736	115.44	A
S366	*Pholcus barisan*	Indonesia, Sumatra, Bukit Barisan	-5.528	104.424	A
P0250	*Pholcus beijingensis*	China, Beijing, Fangshan, Xiayunling Town, Sihe Village, Bianfu Cave	39.73	115.75	D
S064	*Pholcus bicornutus*	Philippines, Luzon, Rizal Prov., near Antipolo, Mystical Cave	14.606	121.209	A
S012	*Pholcus bikilai*	Ethiopia, Southern Nations, Nationalities, and Peolpe's Region..., Wondo Genet	7.076	38.659	A/J
P0147	*Pholcus bourgini*	Guinea, Guinée Forestière, Forêt Classée de Ziama	8.403	-9.322	A
S266	*Pholcus buatong*	Thailand, Krabi, ~10 km N Krabi town, Tham Buatong	8.181	98.884	A
S271	*Pholcus bukittimah*	Singapore, Dairy Farm Nature Park	1.36	103.778	A
S364	*Pholcus camba*	Indonesia, Sulawesi, Bantimurung, at rocks along river	-5.015	119.686	A
P0247	*Pholcus cf jaegeri*	Laos, Khammouane, Nam Kading National Protected Area	18.18	104.475	A
P0162	*Pholcus chappuisi*	Kenya, Central Prov., Mt. Kenya, Mau Mau Cave	-0.217	37.137	A
P0201	*Pholcus chappuisi*	Kenya, Rift Valley, Mt. Elgon National Park, Kitum Cave	1.03	34.757	A
P0148	*Pholcus chattoni*	Guinea, Guinée Forestière, Mount Nimba	7.692	-8.408	A
S101	*Pholcus chengpoi*	Taiwan, Pingtung, near Chouqia	22.241	120.855	A
S482	*Pholcus cibodas*	Indonesia, Java, Cidahu, Javana Spa	-6.737	106.714	A
S324	*Pholcus creticus*	Greece, Crete, Cave near Kritsa	35.16	25.618	L
P0182	*Pholcus crypticolens*	Japan, Hiroshima Pref., Hiroshima-shi, Asakita-ku, Fukawa	34.48	132.53	D
P0222	*Pholcus dade*	USA, Georgia, Dade Co., Sitton's Cave	34.85	-85.467	D
P0172	*Pholcus debilis*	Cameroon, Southwest Region, Mt. Koupé above Nyasoso	4.827	9.685	A
P0225	*Pholcus dentatus*	Portugal, Madeira, Ponta do Sol, Tunel do Lugar de Baixo	32.681	-17.098	D
S258	*Pholcus diopsis*	Thailand, Yala, Wat Kuhapimuk, Tham Meud	6.528	101.225	A
GB13	*Pholcus domingo*	Philippines, Mindanao, Santo Domingo	7.782	125.397	A
S013	*Pholcus domingo*	Philippines, Mindanao, Santo Domingo	7.782	125.397	A

code	species	locality	latitude	longitude	biome
P0149	*Pholcus doucki*	Guinea, Moyenne-Guinée, near Doucki, canyon	10.993	-12.588	A
S259	*Pholcus erawan*	Thailand, Kanchanaburi, Erawan National Park, forest along stream	14.37	99.146	A
S260	*Pholcus erawan*	Malaysia, Pulau Pinang, Penang National Park near Teluk Bahang	5.462	100.202	A
S024	*Pholcus ethagala*	Sri Lanka, Uva Prov., Badulla Distr., Bet. Diyaluma Falls & Wellawaya	6.725	81.029	B
SB017	*Pholcus ethagala?*	Sri Lanka, Western Prov., Gampaha Distric, Alawala	7.123	80.186	B
P0158	*Pholcus fagei*	Kenya, Coast Prov., Arabuko-Sokoke National Park	-3.283	39.967	A
P0159	*Pholcus fagei*	Kenya, Coast Prov., Shimba Hills, near road Kwale-Kinango	-4.203	39.418	A
P0287	*Pholcus fengcheng*	China, Liaoning, Fengcheng City, Mt. Fenghuang	40.426	124.071	D
S105	*Pholcus fragillimus*	Taiwan, Taichung, Tunghai University campus	24.176	120.6	A
S047	*Pholcus G044*	Cape Verde, Fogo, Cueva de Gancho	14.924	-24.334	B
S261	*Pholcus gombak*	Malaysia, Selangor, Kemensah, forest along stream	3.222	101.793	A
P0202	*Pholcus Gui32*	Guinea, Basse-Guinée, near Koumbaya	10.172	-12.888	G
S262	*Pholcus halabala*	Thailand, Narathiwat, Hala Bala Wildlife Sanctuary, 'site 1', forest at river near headquarters	5.797	101.832	A
S365	*Pholcus hurau*	Indonesia, Sumatra, Gua Limpa (Cave) above Harau Valley	-0.104	100.68	A
S299	*Pholcus jinwum*	Australia, Queensland, Kings Plains, Cooktown	-15.485	145.256	G
P0288	*Pholcus jiuwei*	China, Liaoning, Benxi City, Xing'an Village	42.29	123.76	D
P0145	*Pholcus kakum*	Guinea, Guinée Forestière, Forêt Classée de Ziama	8.403	-9.322	A
GB44	*Pholcus kawit*	Philippines, Mindanao, Mt. Matutum, Kawit Forest, 'site 1'	6.338	125.104	A
S225	*Pholcus kawit*	Philippines, Mindanao, Mt. Matutum, Kawit Forest, 'site 1'	6.338	125.104	A
S367	*Pholcus kerinci*	Indonesia, Sumatra, Bukit Barisan	-5.528	104.424	A
S268	*Pholcus khaolek*	Thailand, Nakhon Si Thammarat, Khao Nan National Park, Tham Khao Lek	8.768	98.728	A
P0248	*Pholcus kimi*	Laos, Oudomxay, ca 3 km E Tad Lak 11	20.605	102.022	A
P0203	*Pholcus kindia*	Guinea, Basse-Guinée, near Kindia	10.013	-12.81	G
S186	*Pholcus kipungit*	Malaysia, Sabah, Poring Hot Springs, forest near beginning of Kipungit Trail	6.048	116.706	A/J
S502	*Pholcus kottawagamaensis*	Sri Lanka, Southern Prov., Kottawa Forest	6.097	80.308	A/B
S267	*Pholcus krabi*	Thailand, Krabi, Khao Phanom Bencha National Park, trails near headquarters	8.235	98.918	A
P0171	*Pholcus kribi*	Cameroon, South Region, between Kribi and Campo, 'site 2'	2.673	9.857	A
S265	*Pholcus kuhapimuk*	Thailand, Yala, Wat Kuhapimuk, Tham Meud	6.528	101.225	A
S183	*Pholcus lambir*	Malaysia, Sarawak, Lambir Hills National Park	4.203	114.04	A
S263	*Pholcus ledang*	Malaysia, Johor, Gunung Ledang, forest near Puteri Falls	2.355	102.635	A
P0163	*Pholcus leruthi*	Kenya, Western Prov., Kakamega Forest National Park, Arboretum trail	0.355	34.858	G
S185	*Pholcus lintang*	Malaysia, Sarawak, Bako National Park, along Lintang Trail	1.718	110.452	A
JA42	*Pholcus manueli*	USA, Pennsylvania, ~20 mi NE Stroudsburg	41.217	-74.867	D
BB19	*Pholcus manueli (sp1 Bruvo)*	USA, Pennsylvania, ~20 mi NE Stroudsburg	41.217	-74.867	D
S056	*Pholcus maronita?*	Israel, Judea and Samaria Distr., 'En Perat, Nahal Perat	31.833	35.311	L
S503	*Pholcus maturata*	Sri Lanka, Central Prov., Hakgala Forest	6.93	80.814	A
S226	*Pholcus matutum*	Philippines, Mindanao, Mt. Matutum, Kawit Forest, 'site 1'	6.338	125.104	A
S368	*Pholcus mentawir*	Indonesia, Borneo, East Kalimantan, Bukit Bankirai	-1.029	116.867	A
S053	*Pholcus moca*	Gabon, Ogooué-Ivindo, near Djidji	0.213	11.822	A
P0230	*Pholcus MRAC553*	Congo DR, Orientale, Basoko (Bokungu), old palm plantation	1.233	23.6	A
S188	*Pholcus mulu*	Malaysia, Sarawak, Gunung Mulu National Park, forest near Deer Cave	4.027	114.818	A
S264	*Pholcus narathiwat*	Thailand, Narathiwat, Hala Bala Wildlife Sanctuary, 'site 2', forest near river	5.807	101.81	A
S273	*Pholcus negara*	Thailand, Narathiwat, Hala Bala Wildlife Sanctuary, 'site 2', forest near river	5.807	101.81	A
GB45	*Pholcus nkoetye*	Cameroon, South Region, near Ebolowa, Nkoetye	2.857	11.362	A
P0270	*Pholcus nkoetye*	Cameroon, South Region, near Ebolowa, Nkoetye	2.857	11.362	A
GB46	*Pholcus olangapo*	Philippines, Luzon, near Olangapo, along river near Pamulaklakin Forest Trail	14.798	121.338	A
S014	*Pholcus olangapo*	Philippines, Luzon, near Olangapo, along river near Pamulaklakin Forest Trail	14.798	121.338	A
BB17	*Pholcus opilionoides*	Austria, Upper Austria, Reitpoidl near Linz	48.38	14.277	SA
P0232	*Pholcus ornatus*	Spain, Canary Isl., La Palma, Barranco de Agua (Los Tilos)	28.79	-17.8	L
S065	*Pholcus pagbilao*	Philippines, Negros, Negros Oriental Prov., Casaroro Falls	9.281	123.208	A
S072	*Pholcus pagbilao*	Philippines, Luzon, between Lucban and Tayabas	14.063	121.567	A
BB18a	*Pholcus phalangioides*	Austria, Vienna, Vienna Ciy, house	48.228	16.371	SA
JA43	*Pholcus phalangioides*	Germany, Nordrhein-Westfalen, Bonn, Museum Koenig	50.722	7.113	SA
P0286	*Pholcus phoenixus*	China, Liaoning, Zhangjiagon Village, cave	40.507	124.176	D
S274	*Pholcus phui*	Thailand, Narathiwat, Hala Bala Wildlife Sanctuary, 'site 2', forest near river	5.807	101.81	A
S236	*Pholcus pingtung*	Taiwan, Pingtung, Sheding Nature Park near Kenting	21.957	120.821	A
S051	*Pholcus punu*	Gabon, Ogooué-Ivindo, near Mohoba Mozeye	0.28	13.348	A
S369	*Pholcus quinquenotatus*	Indonesia, Bali, Munduk	-8.267	115.055	A
S052	*Pholcus rawiriae*	Gabon, Ogooué-Ivindo, Monts de Belinga, forest near Mayebout	1.112	13.11	A
S182	*Pholcus sabah*	Malaysia, Sabah, Sepilok, Rainforest Discovery Centre, forest along Pitta Trail	5.877	117.94	A

code	species	locality	latitude	longitude	biome
S275	*Pholcus satun*	Thailand, Satun, Thaleban National Park, forest near headquarters	6.71	100.17	A
GB47	*Pholcus schwendingeri*	Thailand, Ranong, Klong Nakha Wildlife Sanctuary, 40 m a.s.l., forest	9.46	98.512	A
S276	*Pholcus schwendingeri*	Thailand, Ranong, Klong Nakha Wildlife Sanctuary, 40 m a.s.l., forest	9.46	98.512	A
S370	*Pholcus sepaku*	Indonesia, East Kalimantan, Bukit Bankirai	-1.029	116.867	A
S371	*Pholcus singalang*	Indonesia, Sumatra, Lembah Anai Forest Reserve, forest along stream	-0.472	100.363	A
S234	*Pholcus SMF422*	Laos, Bolikhamsay, Nam Kading National Protected Area	18.357	104.155	A
S104	*Pholcus spilis*	Taiwan, Taichung, ~12 air km E Dongshi, near river	24.288	120.946	A
GB48	*Pholcus sudhami*	Thailand, Kanchanaburi, Erawan N.P., 85 m a.s.l., forest along stream	14.37	99.146	A
S277	*Pholcus sudhami*	Thailand, Kanchanaburi, Erawan N.P., 85 m a.s.l., forest along stream	14.37	99.146	A
S471	*Pholcus taarab*	Tanzania, Morogoro, Udzungwa Mts National Park, Sanje chini	-7.774	36.897	A/G
P0155	*Pholcus taita*	Kenya, Coast Prov., Taita Hills, Ngangao Forest	-3.37	38.34	A
S472	*Pholcus tambunan*	Malaysia, Sabah, Crocker Range between Kota Kinabalu and Tambuan, N-slope	5.834	116.336	J
S272	*Pholcus tanahrata*	Malaysia, Pahang, Cameron Highlands, forest along 'trail 9'	4.46	101.392	A
P0258	*Pholcus twa*	Uganda, Kisoro Distr., Mgahinga Gorilla National Park, at building	-1.353	29.618	A
S269	*Pholcus ubin*	Singapore, Pulau Ubin, degraded forest near park headquarters	1.403	103.97	A
GB49	*Pholcus uludong*	Malaysia, Pahang, Ulu Dong, 190 m a.s.l., forest near river	3.942	102.027	A
S270	*Pholcus uludong*	Malaysia, Pahang, Ulu Dong, 190 m a.s.l., forest near river	3.942	102.027	A
S473	*Pholcus wahehe*	Tanzania, Morogoro, Udzungwa Mts National Park, Sanje chini	-7.774	36.897	I
JA71	*Physocyclus dugesi*	Costa Rica, San José, San Pedro de Montes de Oca	9.94	-84.05	C
S474	*Physocyclus enaulus*	USA, Texas, Big Bend National Park, Santa Elena, Terlingua Creek	29.167	-103.611	M
JA70	*Physocyclus franckei*	Mexico, Hidalgo, Reserva de Meztitlán	20.6	-98.766	M
BB21	*Physocyclus globosus*	Cuba, La Habana	23.12	-82.42	SA
OG29	*Pimoa breuili*	–			na
OG32	*Pimoa sp*	–			na
S475	*Pisaboa silvae*	Brazil, Amazonas, forest near Tabatinga	-4.244	-69.925	A
S372	*Platnicknia Cu12-100*	Cuba, Guantanamo, Baracoa, El Yunque, forest on eastern slope	20.345	-74.566	A
S115	*Platnicknia Cu12-2*	Cuba, Pinar del Río, Viñales, forest at base of mogote, 'site 2'	22.627	-83.735	A
S373	*Platnicknia Cu12-61*	Cuba, Camagüey, Sierra de Cubitas, Estación Limones-Tuabaquey	21.597	-77.786	A
S374	*Platnicknia Cu12-99*	Cuba, Guantanamo, Baracoa, forest along Duaba river	20.332	-74.569	A
IA07	*Platnicknia incerta*	Cuba, Santiago de Cuba, Estación Biológica La Platica, forest above station	20.01	-76.889	A
S114	*Platnicknia incerta*	Cuba, Santiago de Cuba, Estación Biológica La Platica, forest above station	20.01	-76.889	A
OG15	*Plectreurys tristis*	–			na
JA80	*Priscula Astr07*	Peru, Ayacucho, Ciudad de Ayacucho, Wari ruins	-13.15	-74.217	A
JA114	*Priscula binghamae*	Bolivia, La Paz, La Paz	-16.5	-68.167	A
JA22	*Priscula Ven02/80-85*	Venezuela, Monagas, Cueva del Guacharo to Salto la Palia	10.175	-63.558	A
S307	*Priscula Ven02/80-85*	Venezuela, Monagas, Cueva del Guacharo to Salto la Palia	10.175	-63.558	A
S476	*Priscula venezuelana*	Venezuela, Aragua, Parque Nacional Henri Pittier, Rancho Grande	10.35	-67.683	A
S477	*Psilochorus concolor*	USA, Texas, Big Bend National Park, Cat Tail Fall	29.273	-103.336	C
S046	*Psilochorus imitatus*	USA, Colorado, Clear Creek Co.	39.75	-105.567	na
BB24	*Psilochorus imitatus=sp.1 Bruvo*	USA, Colorado, El Paso Co., Garden of the Gods	38.88	-104.87	na
S478	*Psilochorus pallidulus*	USA, Texas, Big Bend National Park, Tuff Canyon	29.15	-103.488	M
P0210	*Psilochorus SA Br10-36*	Brazil, Espirito Santo, Vargem Alta, Fazenda Monte Verde	-20.465	-40.995	A
S032	*Psilochorus SA Br11-40*	Brazil, Bahia, Reserva Particular do Patrimônio Natural Serra Bonita	-15.388	-39.563	A
BB12a	*Psilochorus SA itaguyrussu*	Brazil, São Paulo, São Paulo, Parque Estadual de Cantareira	-23.4	-46.58	A
P0219	*Psilochorus SA itaguyrussu*	Brazil, São Paulo, São Paulo, Parque Estadual de Cantareira	-23.4	-46.58	A
BB23	*Psilochorus simoni*	Germany, Nordrhein-Westfalen, Bonn, Museum Koenig	50.722	7.113	SA
BB25	*Quamtana bonamanzi*	South Africa, KwaZulu Natal, Bonamanzi Reserve	-28.067	32.3	A
GB50	*Quamtana bonamanzi*	South Africa, KwaZulu Natal, Bonamanzi Reserve	-28.067	32.3	A
P0183	*Quamtana Cam117*	Cameroon, Northwest Region, near Oku village	6.25	10.5	A
S291	*Quamtana CAS5*	Madagascar, Fianarantsoa, Andringitra National Park, 34 km S Ambalavao	-22.157	46.952	A
P0241	*Quamtana CAS7*	South Africa, Eastern Cape, Colchester, Schelm Hoek, W of Sundays River	-33.701	25.835	L
BB26	*Quamtana embuleni*	South Africa, Mpumalanga, Badplaas, Embuleni Reserve	-25.953	30.553	G/J
P0285	*Quamtana filmeri*	South Africa, KwaZulu Natal, Royal Natal National Park, Cascade falls pathway	-28.69	28.937	J
P0262	*Quamtana kabale*	Uganda, Kasese Distr., Ruwenzori Mts, near National Park gate	0.355	30.027	A/J
S063	*Quamtana kitahurira*	Guinea, Guinée Forestière, Foret de Zié near Gouan	7.6	-8.46	A/G
P0204	*Quamtana nyahururu*	Kenya, Rift Valley, Thompson Falls near Nyahururu	0.045	36.368	A
P0243	*Quamtana nylsvley?*	South Africa, Eastern Cape, Kai Mouth, 58 km NE East London	-32.687	28.377	A/G
P0170	*Quamtana oku*	Cameroon, Northwest Region, near Oku village	6.237	10.525	A
P0284	*Quamtana umzinto*	South Africa, KwaZulu Natal, Oribi Gorge Nature Reserve, Samango Waterfall trail	-30.71	30.27	A
BB27	*Quamtana vidal*	South Africa, KwaZulu Natal, Cape Vidal	-28.13	32.55	A
OG3	*Rhode scutiventris*	–			na
S279	*Savarna kaeo*	Thailand, Prachuap Khiri Khan, Khao Sam Roi Yot National Park, Tham Sai	12.18	100.007	A
S278	*Savarna kraburiensis*	Thailand, Ranong, Tham Phra Kayang	10.326	98.765	A
S306	*Savarna miser*	Malaysia, Selangor, Batu Caves	3.239	101.683	A

code	species	locality	latitude	longitude	biome
S280	*Savarna tessellata*	Thailand, Yala, Wat Kuhapimuk, Tham Meud	6.528	101.225	A
S281	*Savarna thaleban*	Thailand, Satun, Thaleban National Park, forest and cave entrance	6.726	100.162	A
OG25	*Segestria bavarica*	–			na
OG21	*Sicarius terrosus*	–			na
S504	*Sihala ceylonica*	Sri Lanka, North Western Prov., Kurunegala, at base of Ethagala (Athugala) Mtn	7.49	80.369	A
S505	*Sihala SL43*	Sri Lanka, Uva Prov., Inginiyagala	7.225	81.535	B
S506	*Sihala SL63*	Sri Lanka, Sabaragamuwa Prov., above Dematagala	6.451	80.751	A/B
GB51	*Smeringopina ankasa*	Ghana, Western Region, Ankasa NP	5.217	-2.651	A
S054	*Smeringopina ankasa*	Ghana, Western Region, Ankasa NP	5.217	-2.651	A
P0173	*Smeringopina attuleh*	Cameroon, Southwest Region, near Dschang, Essotah	5.425	9.912	A
S491	*Smeringopina bamenda*	Cameroon, Northwest Region, near Bamenda	6.008	10.302	A
GB14	*Smeringopina bayaka*	Gabon, Moyen-Ogooué, near Ndjolé, 'site 2'	-0.098	10.873	A
GB15	*Smeringopina belinga*	Gabon, Ogooué-Ivindo, Monts de Belinga	0.978	13.192	A
P0152	*Smeringopina bineti*	Guinea, Moyenne-Guinée, Dalaba	10.677	-12.267	A
S300	*Smeringopina bomfobiri*	Ghana, Ashanti Region, Bomfobiri Wildlife Sanctuary	6.962	-1.191	A
S492	*Smeringopina bwiti*	Gabon, Ngounié, near Moulandoufouala	-1.635	10.708	A/G
S042	*Smeringopina camerunensis*	Cameroon, Littoral Region, near Douala	4.01	9.798	A/N
GB16	*Smeringopina chaillu*	Gabon, Ngounié, Massif du Chaillu, 'site 4', between Yéno and Mouila	-1.728	11.307	A
S404	*Smeringopina djidji*	Gabon, Ogooué-Ivindo, near Djidji	0.213	11.822	A
GB52	*Smeringopina ebolowa*	Cameroon, South Region, near Ebolowa	2.915	11.138	A
P0175	*Smeringopina ebolowa*	Cameroon, South Region, near Ebolowa	2.915	11.138	A
GB17	*Smeringopina essotah*	Cameroon, Southwest Region, near Dschang, Essotah	5.425	9.912	A
GB18	*Smeringopina fang*	Gabon, Ogooué-Ivindo, near Djidji	0.213	11.822	A
P0153	*Smeringopina guineensis*	Guinea, Guinée Forestière, Forêt Classée de Diéké	7.533	-8.832	A
GB19	*Smeringopina iboga*	Gabon, Ngounié, near Moulandoufouala	-1.635	10.708	G
S041	*Smeringopina kala*	Cameroon, Centre Region, near Yaoundé, Mt. Kala	3.85	11.338	A
S402	*Smeringopina kinguele*	Gabon, Estuaire, Monts de Cristal, between Tchimbélé and Kinguélé	0.563	10.313	A
S044	*Smeringopina kribi*	Cameroon, South Region, between Kribi and Campo, 'site 1'	2.703	9.863	A
GB20	*Smeringopina lekoni*	Gabon, Haut Ogooué, forest at Lékoni River	-1.178	13.538	G
S043	*Smeringopina mbouda*	Cameroon, West Region, near Mbouda, Bamboutos	5.622	10.112	A
S403	*Smeringopina mohoba*	Gabon, Ogooué-Ivindo, near Mohoba Mozeye	0.28	13.348	A
S049	*Smeringopina moudouma*	Gabon, Ogooué-Lolo, near Moudouma	-1.392	12.16	A
GB21	*Smeringopina ndjole*	Gabon, Moyen-Ogooué, near Ndjolé, 'site 1'	-0.15	10.667	A
P0180	*Smeringopina nyasoso*	Cameroon, Southwest Region, Mt. Koupé above Nyasoso	4.827	9.685	A
S405	*Smeringopina ogooue*	Gabon, Ogooué-Ivindo, Monts de Belinga, NE Makokou	0.783	13.138	A
GB22	*Smeringopina sahoue*	Gabon, Estuaire, forest near Sahoué, N Libreville	0.577	9.34	N
S493	*Smeringopina simintang*	Gabon, Ogooué-Ivindo, Ivindo National Park near Simintang	0.537	12.688	A
S301	*Smeringopina tchimbele*	Gabon, Estuaire, Monts de Cristal, between Tchimbélé and Kinguélé	0.563	10.313	A
GB54	*Smeringopina tebe*	Gabon, Ogooué-Ivindo, N Tébé	-0.038	13.682	A
S050	*Smeringopina tebe*	Gabon, Ogooué-Ivindo, N Tébé	-0.038	13.682	A
S017	*Smeringopus arambourgi*	Ethiopia, Oromia, Sof Omar village	6.903	40.851	A
P0224	*Smeringopus atomarius?*	Namibia, Hardap, ~30 km W Betta	-25.32	16.16	M
P0266	*Smeringopus bujongolo*	Uganda, Kasese Distr., Ruwenzori Mts, near Nyabitaba Hut	0.358	29.978	J
P0161	*Smeringopus chogoria*	Kenya, Eastern Prov., Mt. Kenya National Park, Chogoria Forest, 'site 1'	-0.202	37.49	A
P0143	*Smeringopus cylindrogaster*	Guinea, Guinée Forestière, Forêt Classée de Diéké	7.533	-8.832	A
P0178	*Smeringopus cylindrogaster*	Cameroon, South Region, near Kribi	2.9	9.907	A
GB24	*Smeringopus isangi*	Congo DR, Orientale, Yaekama (Isangi)	0.767	24.3	A
S407	*Smeringopus lesserti*	Gabon, Ogooué-Ivindo, Monts de Belinga, Mayebout	1.115	13.107	A
S408	*Smeringopus lotzi*	South Africa, Free State, Erfenis Dam Nature Reserve	-28.498	26.806	M
P0257	*Smeringopus mgahinga*	Uganda, Kisoro Distr., Mgahinga Gorilla National Park, Garama Cave	-1.357	29.632	A
P0265	*Smeringopus mpanga*	Uganda, Bundibugyo Distr., between Sempaya and Itajo	0.838	30.173	A/G
BB28	*Smeringopus natalensis*	South Africa, Mpumalanga, Badplaas, Embuleni Reserve	-25.953	30.553	J
P0157	*Smeringopus ngangao*	Kenya, Coast Prov., Taita Hills, Ngangao Forest	-3.37	38.34	A
P0264	*Smeringopus pallidus*	Uganda, Bushenyi Distr., near Ryeru, in hotel	-0.262	30.105	SA
S406	*Smeringopus peregrinoides*	Uganda, Kanungu Distr., Buhoma	-0.982	29.617	G
P0227	*Smeringopus similis?*	Namibia, Otjozondjupa, Otavi town, camp	-19.633	17.333	G
GB55	*Spermophora akwamu*	Ghana, Central Region, Kakum Forest	5.348	-1.384	A
S018	*Spermophora akwamu*	Ghana, Central Region, Kakum Forest	5.348	-1.384	A
P0187	*Spermophora awalai*	Cameroon, Southwest Region, Mt. Koupé above Nyasoso	4.827	9.685	A
GB56	*Spermophora bukusu*	Kenya, Rift Valley, Mt. Elgon N.P., along Kimothon River	1.077	34.725	A
P0273	*Spermophora bukusu*	Kenya, Rift Valley, Mt. Elgon N.P., along Kimothon River	1.077	34.725	A
S376	*Spermophora cf akwamu*	Gabon, Ogooué-Ivindo, Monts de Belinga, NE Makokou	0.783	13.138	A
GB27	*Spermophora dieke*	Guinea, Guinée Forestière, Forêt Classée de Diéké	7.533	-8.832	A

code	species	locality	latitude	longitude	biome
S055	*Spermophora dieke*	Guinea, Guinée Forestière, Forêt Classée de Diéké	7.533	-8.832	A
S019	*Spermophora estebani*	Philippines, Luzon, between Lucban and Tayabas	14.063	121.567	A
S375	*Spermophora Ind27*	Indonesia, Sumatra, Bukittinggi, degraded forest in canyon, 'site 1'	-0.301	100.344	A
S335	*Spermophora Ind90*	Indonesia, Sulawesi, Susuripen Cave on Mt. Mahawu near Tomohon	1.341	124.864	A
P0189	*Spermophora kirinyaga*	Kenya, Eastern Prov., Mt. Kenya National Park, Chogoria Forest, 'site 2'	-0.237	37.567	G
GB57	*Spermophora kyambura*	Uganda, Bushenyi Distr., Kyambura River Gorge	-0.178	30.097	A
P0174	*Spermophora kyambura*	Cameroon, South Region, near Ebolowa	2.915	11.138	A
P0263	*Spermophora kyambura*	Uganda, Bushenyi Distr., Kyambura River Gorge	-0.178	30.097	A
P0184	*Spermophora maathaiae*	Kenya, Eastern Prov., Mt. Kenya National Park, Chogoria Forest, 'site 1'	-0.202	37.49	J
S282	*Spermophora Mal2*	Singapore, Dairy Farm Nature Park	1.36	103.778	A
GB58	*Spermophora mau*	Kenya, Rift Valley, Mau Escarpment near Elburgon	-0.422	35.788	A
P0275	*Spermophora mau*	Kenya, Rift Valley, Mau Escarpment near Elburgon	-0.422	35.788	A
P0164	*Spermophora minotaura*	Kenya, Rift Valley, Thompson Falls near Nyahururu	0.045	36.368	A
P0186	*Spermophora minotaura*	Kenya, Central Prov., Mt. Kenya National Park, Naro Moru Forest	-0.173	37.21	A
P0272	*Spermophora minotaura*	Kenya, Eastern Prov., Mt. Kenya National Park, Chogoria Forest, 'site 1'	-0.202	37.49	A
P0278	*Spermophora minotaura*	Kenya, Central Prov., Mt. Kenya National Park, above Naro Moru Forest, small cave	-0.167	37.233	A
P0226	*Spermophora MRAC525*	Congo DR, Equator, Mbangi, old secondary forest	2.117	21.733	A
S020	*Spermophora Phi109*	Philippines, Mindanao, CEDAR (Center for Ecological Development and Recreation)	8.251	125.034	A
BB29	*Spermophora senoculata*	USA, New York, New York City, house	40.716	-73.776	SA
S311	*Spermophora senoculata*	Greece, Crete, Praisos	35.123	26.09	SA
S479	*Spermophora tonkoui*	Guinea, Guinée Forestière, Mt. Nimba, Pompage Zié	7.6	-8.46	A
GB59	*Spermophora ziama*	Guinea, Guinée Forestière, Forêt Classée de Ziama	8.403	-9.322	A
P0185	*Spermophora ziama*	Guinea, Guinée Forestière, Forêt Classée de Ziama	8.403	-9.322	A
P0256	*Spermophorides cuneata*	Spain, Canary Isl., Gran Canaria, Rinar de Tamadaba	28.058	-15.688	L
NA001	*Spermophorides fuerteventurensis*	Spain, Canary Isl., La Palma, Lomas de Doña María	28.64	-17.834	L
NA002	*Spermophorides mercedes*	Spain, Canary Isl., Fuerteventura, Jandía, Barranco de los Canarios	28.109	-14.303	L
S308	*Stenosfemuraia cuadrata*	Venezuela, Aragua, Colonia Tovar	10.417	-67.3	A
S108	*Stygopholcus absoloni?*	Bosnia-Herzegovina, Trebinje, Ilijina pecina	42.719	18.303	L
S109	*Stygopholcus absoloni?*	Montenegro, near Nikši?, Studena?ka pecina	42.768	18.931	L
S112	*Stygopholcus absoloni?*	Montenegro, near Zve?ava, Pe?ina u Ivici (cave)	42.538	18.621	L
S110	*Stygopholcus Bal3*	Bosnia-Herzegovina, Vjetrenica pe?ina	42.847	17.984	L
S317	*Stygopholcus photophilus*	Greece, Crete, Episkopi	35.342	24.765	L
BB31	*Systenita prasina*	Venezuela, Aragua, Colonia Tovar	10.417	-67.3	A
JA88	*Systenita prasina*	Venezuela, Aragua, Colonia Tovar	10.417	-67.3	A
JA131	*Tainonia samana*	Dom. Rep., Samaná, S Las Galeras	19.213	-69.218	A
S480	*Tainonia serripes*	Dom. Rep., San Cristóbal, near Medina	18.513	-70.123	A
JA134	*Tainonia visite*	Haiti, Dept Sud-Est, La Visite National Park, Case Dent	18.337	-72.273	C
OG31	*Tetragnatha versicolor_A*	–			na
S302	*Trichocyclus balladong*	Australia, Western Australia, Murchison, Mid West Region, Mt. Forrest	-28.745	119.94	L
BB32	*Trichocyclus cf balladong*	Australia, Western Australia, Gundaring Nature Reserve	-31.853	117.632	L
S303	*Trichocyclus cf warianga*	Australia, Western Australia, Fortescue, Pilbara Region, Kutayi, drill hole ML0701	-22.54	120.032	M
S384	*Tupigea ale*	Brazil, Rio de Janeiro, ~3.5 km NW Parati	-23.192	-44.732	A
JA48	*Tupigea angelim*	Brazil, São Paulo, Ubatuba, Fazenda Angelim	-22.615	-52.298	A
S481	*Tupigea angelim*	Brazil, São Paulo, Ubatuba, Fazenda Angelim	-22.615	-52.298	A
S494	*Tupigea Br10-32*	Brazil, Espirito Santo, Vargem Alta, Fazenda Monte Verde	-20.465	-40.995	A
S411	*Tupigea Br10-40*	Brazil, Espirito Santo, Vargem Alta, Fazenda Monte Verde	-20.465	-40.995	A
S409	*Tupigea Br14-47*	Brazil, Santa Catarina, Serra Geral National Park, Churriado Canyon	-29.137	-49.953	A
JA13	*Tupigea cantareira*	Brazil, São Paulo, São Paulo, Parque Estadual de Cantareira	-23.4	-46.58	A
P0218	*Tupigea maza*	Brazil, Rio de Janeiro, Cachoeiras de Macacu, Res. Ecol. Guapiaçú	-22.415	-42.745	A
P0214	*Tupigea nadleri*	Brazil, Rio de Janeiro, Santa Maria Madalena, forest fragment	-21.983	-41.957	A
GB30	*Tupigea paula*	Brazil, Rio Grande do Sul, São Francisco de Paula National Forest	-29.43	-50.392	A
S377	*Tupigea penedo*	Brazil, Rio de Janeiro, ~4 km NW Penedo	-22.408	-44.553	G
S385	*Tupigea teresopolis?*	Brazil, Rio de Janeiro, ~4 km NW Penedo	-22.408	-44.553	A/G
OG12	*Uloborus glomosus*	–			na
OG13	*Uroctea durandi*	–			na
S283	*Uthina huifengi?*	Thailand, Surat Thani, Khao Sok National Park, forest along nature trail	8.913	98.498	A
S378	*Uthina Ind119*	Indonesia, Bali, Lake Tambligan, forest along SE side of lake	-8.265	115.098	A
S379	*Uthina Ind121*	Indonesia, Bali, Lake Tambligan, forest along SE side of lake	-8.265	115.098	A
S380	*Uthina Ind67*	Indonesia, Sulawesi, Bantimurung, Gua Mimpi (cave)	-5.019	119.686	A
S284	*Uthina khaosokensis*	Thailand, Surat Thani, Khao Sok National Park, Tham Panthurat	8.901	98.525	A

code	species	locality	latitude	longitude	biome
S102	*Uthina luzonica*	Taiwan, Pingtung, near Danlu	22.21	120.76	A
S217	*Uthina luzonica*	Malaysia, Sarawak, Niah Cave National Park, forest near headquarters	3.82	113.763	A
S285	*Uthina ratchaburi*	Thailand, Ratchaburi, Wat Huai Takaeng	13.587	99.759	A
SB052	*Wanniyala agrabopath*	Sri Lanka, Central Prov., Nuwara Elliya District, Hakgala Strict Natural Reserve	6.911	80.793	A
S022	*Wanniyala SL71*	Sri Lanka, Southern Prov., Galle Distr., Kottawa	6.098	80.313	A
SB063	*Wanniyala SL61*	Sri Lanka, Uva Prov., Badulla District, along Passara-Ella road (B113), Namunukula	6.867	81.117	A
S507	*Wanniyala SL38*	Sri Lanka, Uva Prov., near Okkampitiya	6.728	81.336	B
S508	*Wanniyala SL55*	Sri Lanka, Sabaragamuwa Prov., above Dematagala	6.451	80.751	A/B
S509	*Wanniyala SL61*	Sri Lanka, Uva Prov., near Ohiya	6.807	80.848	A
S304	*Waunana modesta?*	Colombia, Choco, Jardin Botanico del Pacifico, Trail to Mirador	6.266	-77.375	A
S293	*Wugigarra kalamai*	Australia, Western Australia, Murchison, Mid West Region, Mt. Forrest	-28.673	119.96	L/M
S233	*Wugigarra QMB5*	Australia, Queensland, Boombana National Park	-27.402	152.79	D
P0251	*Wugigarra undanbi*	Australia, Queensland, Boombana National Park	-27.402	152.79	D
S232	*Wugigarra yawai*	Australia, Queensland, Redlands, site 3			D
S381	*Wugigarra? Ind107*	Indonesia, West Papua, Manokwari, Gunung Meja	-0.86	134.084	A
S495	*Wugigarra? Ind117*	Indonesia, Ternate, at Danau Tolire (lake)	0.83	127.311	A
S382	*Wugigarra? Ind82*	Indonesia, Sulawesi, Tinoor Fall near Tomohon	1.382	124.836	A
S496	*Wugigarra? Ind96*	Indonesia, West Papua, Arfak Mountains, forest above Mokwam	-1.112	133.911	A
P0240	*Zatavua analalava?*	Madagascar, Toamasina, Masoala National Patk	-15.708	49.965	A
S045	*Zatavua CAS8*	Madagascar, Toamasina, Masoala National Patk	-15.57	50.003	A
OG33	*Zygiella x-notata*	–			na

Table S3: Forward and reverse primers used in the study.

locus	sequence	reference
12S	12S-ai 5'-AAACTAGGATTAGATACCCTATTAT-3'<br>12S-bi 5'-AAGAGCGACGGGCGATGTGT-3'	Simon et al. (1994)
16S	16s1471-mod: 5'-GCCTGTTTAWCAAAAACAT-3'<br>16sbr-H-mod: 5'-CCGGTYTGAACTCARATCAYGT-3'	Astrin et al. (2006)
18S	18S-7F 5'-GCAATAACAGGTCTGTGATGCCC-3'<br>18S-9R 5'-GATCCTTCCGCAGGTTCACCTAC-3'	Giribet et al. (1996) /<br>Whiting et al. (1997)
28S	28S-B1 5'-GACCGATAGCAAACAAGTACCG-3'<br>28S-B2 5'-GATTAGTCTTTCGCCCCTATA-3'	Bruvo-Madarić et al. (2005)
CO1	LCO1490-JJ2 5'-CHACWAAYCAYAARGAYATYGG-3'<br>HCO2198-JJ2 5'-ANACTTCNGGRTGNCCAAARAATCA-3'	Astrin et al. (2016)
H3	H3aF 5'-ATGGCTCGTACCAAGCAGACVGC-3'<br>H3aR 5'-ATATCCTTRGGCATRATRGTGAC-3'	Blackledge et al. (2009)

Table S4: Codes of the specimens contributing to the respective chimera. Chimera codes are used in the trees.

code	genus	species	12S	16S	18S	28S	CO1	H3
CH01	*Carapoia*	*paraguaensis*	BB02	JA97		JA97	JA97	
CH02	*Leptopholcus*	*dschang*	P0192	P0192	P0192	P0192	P0176	P0192
CH03	*Mesabolivar*	*yuruani*	BB07	JA101		JA101	JA101	
CH04	*Pholcus*	*manueli*	BB19	JA42		BB19	BB19	
CH05	*Priscula*	*Ven02/80-85=sp.1 Astrin*		S307			JA22	S307
CH06	*Systenita*	*prasina*	BB31			BB31	JA88	
CH07	*Modisimus*	*Cu12-11*		IA03		S106	IA03	
CH08	*Platnicknia*	*incerta*		IA07			S114	S114
CH09	*Psilochorus SA*	*itaguyrussu*	BB12a	BB12a	P0219	P0219	P0219	P0219
CH10	*Aetana*	*omayan*	S001	S001	S001	S001	GB02	S001
CH11	*Aetana*	*lambir*			S121	S121	GB04	
CH12	*Carapoia*	*dandarae*		S033	S033	S033	GB05	S033
CH13	*Pholcus*	*domingo*	S013				GB13	
CH14	*Spermophora Afr*	*dieke*	S055	S055	S055		GB27	
CH15	*Aetana*	*loboc*		S069	S069	S069	GB31	S069
CH16	*Aetana*	*manansalai*		S073	S073	S073	GB32	S073
CH17	*Calapnita*	*semengoh*	S135				GB33	S135
CH18	*Carapoia*	*capixaba*	S027	S027	S027	S027	GB34	S027
CH19	*Carapoia*	*gracilis*		S030	S030		GB35	S030
CH20	*Leptopholcus*	*budongo*			P0260	P0260	GB36	P0260
CH21	*Micropholcus*	*baoruco*	P0166		P0166		GB37	P0166
CH22	*Micropholcus*	*piaui*		Mic10	Mic10	Mic10	GB38	Mic10
CH23	*Micropholcus*	*ubajara*			Mic11		GB39	Mic11
CH24	*Panjange*	*iban*	S169			S169	GB40	
CH25	*Panjange*	*kubah*	S171			S171	GB41	
CH26	*Panjange*	*seowi*	S170			S170	GB42	
CH27	*Pholcus*	*bamboutos*	P0181	P0181			GB43	P0181
CH28	*Pholcus*	*kawit*	S225	S225			GB44	
CH29	*Pholcus*	*nkoetye*	P0270	P0270			GB45	P0270
CH30	*Pholcus*	*olangapo*	S014	S014	S014	S014	GB46	
CH31	*Pholcus*	*schwendingeri*	S276	S276		S276	GB47	S276
CH32	*Pholcus*	*sudhami*	S277	S277	S277		GB48	S277
CH33	*Pholcus*	*uludong*	S270		S270	S270	GB49	
CH34	*Quamtana*	*bonamanzi*	BB25			BB25	GB50	
CH35	*Smeringopina*	*ankasa*		S054	S054		GB51	
CH36	*Smeringopina*	*ebolowa*	P0175	P0175			GB52	P0175
CH37	*Smeringopina*	*tebe*	S050	S050	S050		GB54	
CH38	*Spermophora Afr*	*akwamu*	S018		S018		GB55	S018
CH39	*Spermophora Afr*	*bukusu*	P0273	P0273	P0273		GB56	
CH40	*Spermophora Afr*	*kyambura*	P0263	P0263	P0263		GB57	P0263
CH41	*Spermophora Afr*	*mau*	P0275		P0275	P0275	GB58	P0275
CH42	*Spermophora Afr*	*ziama*	P0185		P0185		GB59	P0185
CH43	*Belisana*	*aninaj*	S415		S238	S415	S415	
CH44	*Pholcus*	*andulau*	S469				S469	S178
CH45	*Tupigea*	*angelim*	S481	JA48	S481	S481	JA48	S481
CH46	*Aetana*	*ocampoi*	S007		S007	S007	GB70	S007
CH47	*Artema*	*nephilit*		S325	S325		GB03	
CH48	*Hantu*	*kapit*	S412		S412	S122	S412	S412
CH49	*Psilochorus*	*imitatus*	BB24	S046	S046	BB24	BB24	
CH50	*Carapoia*	*macacu*		P0207	P0207	P0207	P0206	P0207
CH51	*Pholcus*	*phalangioides*	BB18a	BB18a			JA43	
CH52	*Pholcus*	*fagei*	P0158	P0158	P0158		P0159	P0158

Table S5: Calibration points for divergence time estimation used in the employed methods. Units are Million years except for MCMCtree (100 My). BEAST lognormal means are in real space.

	RelTime/treePL	MCMCtree (heavy tailed cauchy)						BEAST (lognormal)		
		tL	p	c	2.5%	mode	97.5%	mean	s.d.	offset
Dysderidae	min 35/125*	0.35	2.57	0.4	0.35	1.25	3.07	0	1	35
Segestriidae	min 125	1.25	0.04	0.1	1.25	1.3	3.8	0	1.5	125
Araneidae	min 115	1.15	0.03	0.1	1.15	1.18	3.59	0	1	115
Pimoidae	min 35	0.35	2.29	0.4	0.35	1.15	2.99	0	1	35
Micropholcus	min 20	0.2	0.63	0.5	0.2	0.33	1.9	0	1.96	20
Macaron. Pholcus	max 14	0.01	6	0.5	0.01	0.07	0.13	6	0.6	0
root	max 400**	max 400								

* treePL: failure to initialize analysis with older nested clade; ** for treePL only

Table S6: Monophyletic groups used for species number estimates (compare Fig. S3). Currently described, misplaced, and unpublished species are listed and sum up to an improved number of currently known species (C-M+U; updated in October 2017). These numbers were multiplied with factor 2 or 3 to obtain an estimate of total species number (see main text for more detailed explanation).

Node	Current	Mispl.	Unpubl.	C-M+U	Mult. by	Estimate
1. Artema	8		3	11	2	22
2. Ninetinae	31		13	44	3	132
3. Priscula	17		7	24	3	72
4. Geneve59 + Chisosa	2		1	3	3	9
5. 'Holocneminus' huangdi + Nita	2			2	3	6
6. Physocyclus	34	-2		32	2	64
7. 'Wugigarra' Indonesia			9	9	3	27
8. Trichocyclus	23		5	28	2	56
9. Wugigarra Australia	22		6	28	3	84
10. Holocneminus (exc. huangdi)	2		6	8	3	24
11. MACN270 + Br16-44			2	2	3	8
12. Br16-50 + Br16-178			3	3	3	9
13. Tupigea	12		9	21	3	63
14. Chibchea	16		12	28	3	84
15. Br16-196			1	1	3	3
16. Br15-45			1	1	3	3
17. Waunana + Pisaboa	8			8	3	24
18. Ciboneya	4			4	2	8
19. Ixchela	20			20	2	40
20. 'Coryssocnemis' Centr. Amer.	4			4	3	12
21. Psilochorus	43	-7		36	2	72
22. 'Psilochorus' South Amer.	2		4	6	3	18
23. Tainonia	5			5	2	10
24. Modisimus + Anopsicus + Platnicknia	147		95	238	2	476
25. Otavaloa + Mesabolivar + Teuia + Litoporus	81	-2	28	107	3	321
26. Stenosfemuraia + Systenita + Mecolaestus + Coryssocnemis	36	-4	6	38	3	114
27. Aymaria	7		2	9	3	27
29. Hoplopholcus	10		8	18	2	36
30. 'Holocnemus' hispanicus	1			1	2	2
31. Stygopholcus	3			3	2	6
32. 'Holocnemus' caudatus	1			1	2	2
33. Crossopriza	7		19	26	2	52
34. Smeringopus (exc. rubrotinctus & chogoria groups)	48		20	68	2	136
35. Smeringopus rubrotinctus + chogoria groups	7			7	2	14
36. Smeringopina guineensis group	3			3	2	6
37. Smeringopina (exc. guineensis group)	41		5	46	3	138
38. 'Spermophora' kyambura	1			1	3	3
39. Spermophora (true)	3			3	2	6
40. 'Spermophora' akwamu etc.	3		1	4	3	12
41. Belisana + Hantu	114		36	150	3	450
42. 'Spermophora' ziama + tonkoui	2			2	3	6
43. Wanniyala	2		6	8	2	16
44. 'Paramicromerys CAS13'			1	1	3	3
45. 'Spermophora' awalai etc.	6		1	7	3	21
46. Spermophorides	34			34	2	68
47. Paramicromerys	14	2	11	27	3	81
48. Savarna	5			5	3	15
49. 'Spermophora' mau etc.	6		2	8	3	24
50. Buitinga	22		4	26	3	78
51. Ind206			1	1	3	3
52. 'Spermophora' SE Asia + Australia	13		7	20	3	60
53. Aetana	18		6	24	3	72

Node	Current	Mispl.	Unpubl.	C-M+U	Mult. by	Estimate
54. Zatavua	17		11	28	3	84
55. Nyikoa	1			1	2	2
56. Anansus	5		1	6	3	18
57. Metagonia	84		35	119	3	357
58. Khorata	33			33	3	99
59. Quamtana	26		10	36	3	108
60. 'Calapnita' phyllicola group	10			10	2	20
61. Pholcus minang group	6	-1	1	6	3	18
62. Pholcus diopsis	1			1	3	3
63. Pholcus ethagala group SE Asia	8			8	3	24
64. Pholcus domingo group	2			2	3	6
65. Pholcus kerinci group	2		1	3	3	9
66. Pholcus ethagala group Sri Lanka	3		1	4	2	8
67. Panjange lanthana group + cavicola group	14		2	16	3	48
68. Pholcus chengpoi group	2			2	3	6
69. Pholcus tambunan group	2			2	3	6
70. Pholcus krabi group	6		1	7	3	21
71. Calapnita (true)	15			15	2	30
72. Pholcus andulau group	2			2	3	6
73. 'Panjange' nigrifrons group	9			9	3	27
74. Pholcus buatong group	3			3	3	9
75. Uthina	14		3	17	2	34
76. Pholcus phungiformes group + yichengicus group + taishan group	112		6	118	2	236
77. Pholcus mentawir	1			1	3	3
78. Pholcus bidentatus group	32		1	33	2	66
79. Micropholcus	15	1	3	19	2	38
80. Pholcus halabala group (incl. quinquenotatus group)	24		2	26	3	78
81. 'Leptopholcus' podophthalmus	2			2	2	4
82. Micromerys	9			9	3	27
83. Pehrforsskalia	3			3	2	6
84. Leptopholcus +	22	-2	3	23	2	46
85. Pholcus opilionoides group + kingi group + crypticolens group	24	-1		23	2	46
86. Pholcus Macaronesia + Cape Verde	25		1	26	2	52
87. Pholcus taarab group	3			3	2	6
88. Pholcus calligaster group	5			5	2	10
89. Pholcus ancoralis group	6			6	3	18
90. Pholcus gracillimus group	9	-1		8	2	16
91. Pholcus bicornutus group	8		2	10	2	20
92. Pholcus kakum + MRAC553 ('bamboutos group' part 2)	1		1	2	3	6
93. Pholcus lamperti group	8			8	3	24
94. Pholcus Gui32	1			1	3	3
95. Pholcus debilis group	4	2		6	3	18
96. Pholcus moca + punu ('bamboutos group' part 1)	2			2	3	6
97. Sihala	2		4	6	2	12
98. Pholcus phalangioides group	11			11	2	22
99. Pholcus chappuisi group	8			8	3	24
100. Pholcus bamboutos ('bamboutos group' part 3)	1			1	3	3
101. Pholcus rawiriae + leruthi ('circularis group' part 1)	3			3	3	9
102. Pholcus guineensis group	5			5	2	10
Totals	1501	-15	430	1912		4850

Table S7: Models of diversification rates that were used with HiSSE. All models were evaluated for all dated trees (MCMCtree, treePL, and RelTime), choosing the best fitting one by the AIC value.

model	hidden states	turnover rates	extinction fractions
1a	no	equal	equal
1b	no	varying	equal
1c	no	equal	varying
1d	no	varying	varying
2a	yes	equal	equal
2b	yes	varying	equal
2c	yes	equal	varying
2d	yes	varying	varying

Table S8: Correlation of tibia to metatarsus ratio of leg 1 among microhabitats inferred with PGLS. (G) = ground; (S) = space; (L) = leave

time tree	G – S	G – L	S – L	p-value
treePL	-0.050	-0.031	0.472	<0.001
MCMCtree	-0.129	-0.098	0.423	<0.001
RelTime	-0.039	-0.029	0.538	<0.001

Table S9: Phylogenetic signal in the tibia to metatarsus ratio of leg 1. Given are Blomberg's K and Pagel's lambda along with the respective p-values.

time tree	K	p_{rand}	lambda	p_{lrt}
treePL	1.9	0.001	0.96	6.9e-164
MCMCtree	2.4	0.001	0.99	1.4e-159
RelTime	1.8	0.001	0.97	5.1e-150

Table S10: Speciation (λ) and extinction (μ) rates estimated with MuSSE for all available pholcid species.

time tree	species numbers	λ_{ground}	λ_{leaf}	λ_{space}	μ_{ground}	μ_{leaf}	μ_{space}
treePL	estimated	0.031	0.129	0.086	0.013	0.112	0.073
MCMCtree	estimated	0.026	0.032[a]	0.032[a]	0.0012[b]	0.0012[b]	0.0012[b]
RelTime	estimated	0.113	0.314	0.118	0.036	0.205	0.012
treePL	curr. descr.	0.016	0.053	0.036	0.000	0.038	0.023
MCMCtree	curr. descr.	0.018	0.022[a]	0.022[a]	0.000[b]	0.000[b]	0.000[b]
RelTime	curr. descr.	0.064	0.143	0.078	0.000	0.047	0.000

[a/b] values marked with the same letter were estimated as one parameter.

Table S11: Speciation (λ) and extinction (μ) rates estimated with MuSSE for tropical species only.

time tree	species numbers	λ_{ground}	λ_{leaf}	λ_{space}	μ_{ground}	μ_{leaf}	μ_{space}
treePL	estimated	0.037	0.095	0.076	0.022	0.076	0.056
MCMCtree	estimated	0.027	0.033[a]	0.033[a]	0.0036[b]	0.0036[b]	0.0036[b]
RelTime	estimated	0.103	0.244	0.164	0.022	0.136	0.053
treePL	curr. descr.	0.015	0.039	0.031	0.000	0.021	0.012
MCMCtree	curr. descr.	0.018	0.022[a]	0.022[a]	0.000[b]	0.000[b]	0.000[b]
RelTime	curr. descr.	0.064	0.096[a]	0.096[a]	0.000[b]	0.000[b]	0.000[b]

[a/b] values marked with the same letter were estimated as one parameter

Table S12: Net diversification rates estimated with MuSSE (all species / tropical species).

time tree	species numbers	ground	leaf	space
treePL	estimated	0.018/0.015	0.017/0.019	0.013/0.020
MCMCtree	estimated	0.025/0.023	0.031/0.029	0.031/0.029
RelTime	estimated	0.077/0.081	0.110/0.110	0.110/0.110
treePL	curr. descr.	0.016/0.015	0.015/0.018	0.013/0.019
MCMCtree	curr. descr.	0.018/0.018	0.022/0.022	0.022/0.022
RelTime	curr. descr.	0.064/0.064	0.096/0.096	0.078/0.096

Table S13: Speciation (λ) and extinction (μ) rates estimated with HiSSE.

tree	species numbers	λ_{ground}	$\lambda_{leaf+space}$	λ_{hidden}	μ_{ground}	$\mu_{leaf+space}$	μ_{hidden}
MCMCtree	curr. descr.	0.0205	0.0205	0.0000	0.0000	0.0000	0.0000
MCMCtree	estimated	0.0313	0.0313	0.0122	0.0018	0.0018	0.0000
RelTime	curr. descr.	0.2228	0.2247	0.0445	0.1197	0.1178	0.0096
RelTime	estimated	0.5697	0.5716	0.0820	0.4707	0.4688	0.0271
treePL	curr. descr.	0.0085	0.0578	0.0457	0.0044	0.0300	0.0241
treePL	estimated	0.1447	0.1514	0.0163	0.1281	0.1214	0.0000

Table S14: Net diversification rates and best fit models estimated with HiSSE.

tree	species numbers	ground	leaf+space	hidden	best fit model
MCMCtree	curr. descr.	0.0205	0.0205	0.0000	no hidden states, equal turnover rates and extinction fractions
MCMCtree	estimated	0.0295	0.0295	0.0122	hidden states, equal turnover rates and extinction fractions
RelTime	curr. descr.	0.1031	0.1069	0.0348	hidden states, equal turnover rates and varying extinction fractions
RelTime	estimated	0.0990	0.1028	0.0549	hidden states, equal turnover rates and varying extinction fractions
treePL	curr. descr.	0.0041	0.0278	0.0217	hidden states, varying turnover rates and equal extinction fractions
treePL	estimated	0.0165	0.0300	0.0163	hidden states, equal turnover rates and varying extinction fractions
